# Multimodal Intervention and Child Passenger Safety Guideline Adherence in Young Children: A Sequential, Multiple-Assignment, Randomized Clinical Trial

**DOI:** 10.1001/jamanetworkopen.2025.33912

**Published:** 2025-09-29

**Authors:** Michelle L. Macy, Bethany Pollock, Sadiqa Kendi, Jason Goldstick, Kristine Cieslak, Patrick M. Carter, Ken Resnicow

**Affiliations:** 1 Mary Ann & J. Milburn Smith Child Health Outcomes, Research and Evaluation Center, Stanley Manne Children’s Research Institute, Ann & Robert H. Lurie Children’s Hospital of Chicago, Chicago, Illinois; 2 Division of Emergency Medicine, Department of Pediatrics, Northwestern University Feinberg School of Medicine, Chicago, Illinois; 3 Division of Emergency Medicine, Children’s National Hospital, Department of Pediatrics, George Washington School of Medicine & Health Sciences, Safe Kids Worldwide, Washington, DC; 4 Department of Emergency Medicine, University of Michigan Medical School, Ann Arbor; 5 Department of Health Behavior & Health Education, University of Michigan School of Public Health, Ann Arbor; 6 Injury Prevention Center, University of Michigan, Ann Arbor; 7 Department of Health Behavior & Health Education, University of Michigan School of Public Health, Ann Arbor; 8 Now with: Division of Epidemiology & Community Health, School of Public Health, University of Minnesota, Minneapolis

## Abstract

**Importance:**

Suboptimal child passenger safety behaviors are common despite the availability of effective child restraint systems (CRS), with national guidelines promoting—and state laws requiring—their use.

**Objective:**

To compare 6-month guideline adherence following the Tiny Cargo, Big Deal, Abróchame Bien, Cuídame Bien (TCBD/ABCB) intervention vs enhanced usual care (EUC); assess 12-month maintenance effect; and evaluate sequential, multiple-assignment, randomized trial (SMART) phase 2 outcomes by intervention intensity.

**Design, Setting, and Participants:**

This randomized clinical trial using a SMART study design was conducted at 1 pediatric emergency department (ED), 1 community ED, and 2 urgent care centers in metropolitan Chicago, Illinois, from February 2020 to August 2022. Eligible caregivers spoke English or Spanish, lived in Illinois, owned a smartphone, traveled weekly with their child (aged 6 months to younger than 11 years), and reported suboptimal child passenger safety behaviors. Of 5416 caregivers who were screened, 1517 were potentially eligible. Data analysis occurred from October 2024 to March 2025.

**Interventions:**

Phase 1 included basic TCBD/ABCB (remote motivational interviewing [MI] plus tailored mobile health [mHealth]) vs EUC (text message photograph requests, follow-up activity reminders, and corrective feedback only for predefined critical errors). In phase 2, participants exposed to TCBD/ABCB but who were nonadherent at 6 months received high-intensity (second remote MI session plus extra text messages) or low-intensity (mHealth alone) interventions.

**Main Outcome and Measures:**

The primary outcome was guideline adherence, a dichotomous measure of all 3 criteria: (1) appropriate CRS, (2) back seat location, and (3) never unrestrained. Outcomes were assessed at 6 and 12 months using logistic regression with prevalence differences, adjusted for randomization strata, caregiver gender, race, and ethnicity.

**Results:**

In phase 1, there were 474 participants (mean [SD] caregiver age, 36.1 [6.2] years; 443 mothers [93.5%]; 52 [11.0%] preferred Spanish]) of whom 342 were randomized to basic TCBD/ABCB and 132 to EUC. Among participants with 6-month follow-up data, 131 of 278 caregivers receiving TCBD/ABCB (47.1%; 95% CI, 41.3% to 53.0%) vs 38 of 118 receiving EUC (32.2%; 95% CI, 23.8% to 40.6%) were guideline adherent (absolute change in treatment response, 13.1%; 95% CI, 3.6% to 22.6%; *P* = .007) with 12-month maintenance effects (adjusted prevalence difference, 39.2%; 95% CI, 26.5% to 51.9%; *P* < .001). In phase 2, 185 participants who received TCBD/ABCB and were not guideline adherent were rerandomized to high-intensity (87 participants) or low-intensity (84 participants) intervention. High-intensity TCBD/ABCB had no effect compared with low-intensity TCBD/ABCB (adjusted prevalence difference, −3.9%; 95% CI −17.9% to 10.1%; *P* = .59).

**Conclusions and Relevance:**

In this randomized clinical trial of caregivers, TCBD/ABCB was associated with improved adherence to child passenger safety guidelines with lasting effects among those caregivers who were adherent at 6 months. These findings suggest that this remote precision prevention intervention against a leading cause of death may be adaptable to other settings.

**Trial Registration:**

ClinicalTrials.gov Identifier: NCT04238247

## Key Points

**Question:**

Is motivational interviewing (MI) with tailored mobile health (mHealth) associated with increased child passenger safety guideline adherence?

**Findings:**

In this sequential, multiple-assignment, randomized clinical trial of 474 caregivers of children aged 6 months to younger than 11 years, guideline adherence was 13.1 percentage points higher for basic intervention than for enhanced usual care at 6 months and 39.2 percentage points higher at 12 months among those who were guideline adherent at 6 months. In phase 2, an additional remote MI session plus additional text messages for intervention participants who were not adherent had no effect.

**Meaning:**

This trial found that the basic intervention changed child passenger safety behaviors by 6 months and was associated with maintenance of guideline adherence to 12 months, suggesting that this intervention may protect against a leading cause of death among children.

## Teaser

This sequential, multiple-assignment randomized clinical trial compares 6- and 12-month child passenger restraint system guideline adherence following a multimodal intervention vs usual care.

## Introduction

Child restraint systems (CRS), including car seats and booster seats, are designed to protect child passengers until they are large enough, and developmentally mature enough, to effectively use adult seat belts.^1,2,3,4^ In 1996, the American Academy of Pediatrics first published child passenger safety guidelines.^5^ Their 2011 update,^6^ reaffirmed in 2018,^7^ emphasized size-based transitions through CRS stages. Child passenger laws, of varying strengths, are present nationally.^8,9^ American Academy of Pediatrics guidelines are promoted during well child examinations^10^ and disseminated nationally through AdCouncil campaigns.

In 2022, 450 child passengers younger than 10 years were killed and approximately 80 000 were injured in motor vehicle collisions (MVCs) in the US.^11^ Despite CRS reducing crash-related injury and fatality by 45% to 90% in 2023,^12,13,14,15,16,17^ 14% of children aged 1 to 3 years and 45% of children aged 6 to 7 years were prematurely transitioned to less protective CRSs or traveled unrestrained.^18^

To address pervasive suboptimal child passenger safety behaviors^18,19^ that contribute to preventable MVC-related deaths and injuries,^20,21,22,23,24^ we created Tiny Cargo, Big Deal, Abróchame Bien, Cuídame Bien (TCBD/ABCB), a precision prevention intervention grounded in self-determination theory,^25,26^ and tested it using a sequential, multiple-assignment, randomized trial (SMART) design.^27,28^ We hypothesized that caregivers randomized to basic TCBD/ABCB in phase 1 would be more likely to adopt guideline adherent child passenger safety behaviors than caregivers receiving enhanced usual care (EUC) and that a higher-intensity phase 2 intervention would be needed among caregivers who were nonadherent in phase 1.^29^

## Methods

### Trial Design

We conducted a multisite, randomized clinical trial using a SMART design to test the efficacy of TCBD/ABCB intervention components. The study was approved by the institutional review board at Ann & Robert H. Lurie Children’s Hospital of Chicago and followed the Consolidated Standards of Reporting Trials (CONSORT) reporting guideline. The trial protocol is available in Supplement 1.

### Setting

Caregivers were invited to complete a screening survey during or after their child’s visit to 2 emergency departments (EDs) or 2 urgent care centers affiliated with Lurie Children’s from February 2020 through September 2022. Lurie Children’s is a free-standing children’s hospital located in downtown Chicago, Illinois. Central DuPage Hospital, in suburban Winfield, Illinois, has a general ED and pediatric treatment area. Lurie Children’s urgent care centers are located within Chicago’s Lincoln Park neighborhood and suburban Northbrook, IL. We recruited from acute care settings because these families may have less access to primary care, where injury prevention anticipatory guidance is traditionally provided.

### Participants

English- or Spanish-speaking adult parents or legal guardians (hereafter referred to as caregivers) were eligible if their child (aged 6 months to younger than 11 years) was less than 55 inches tall, used a conventional CRS (eg, not a travel vest or wheelchair), and reported current child passenger safety behaviors that were not guideline adherent (ie, not using an age- and weight-appropriate CRS, using the front seat, or traveling unrestrained) or a planned premature transition within 6 months (ie, will stop using a recommended CRS). We did not contact caregivers of children involved in MVCs, with critical illness or injury (Emergency Severity Index triage category 1), under evaluation for suspected child abuse, or in protective custody. We excluded caregivers who (1) traveled less than 1 time per week with their child in a passenger vehicle, including taxis and ride-share services; (2) enrolled for another child; (3) resided outside of Illinois because child passenger safety laws vary by state; and (4) did not have a smartphone, needed for mobile health (mHealth) intervention components. Participants were enrolled by trained research coordinators (RCs) who shared consent documents via email, reviewed study details by phone, and obtained informed consent with electronic documentation.

### Phase 1

#### Intervention

Basic TCBD/ABCB consisted of a brief (<20-minute) motivational interviewing (MI) counseling session delivered remotely by trained, MI-proficient RCs and counselors (who achieved OnePass^30^ scores of 5 or greater on the 7-point scale) and 2 mHealth components: (1) the Car Seat Compass, a tailored, mobile-friendly web-application and (2) personalized text messages. Intervention development and fidelity monitoring were previously published.^27^ We tailored web-application content and 2 informational or motivational text messages per month based on baseline and 6-month survey responses. Every 4 to 6 weeks, we asked caregivers to submit photographs depicting their child as they usually travel. Two study team members (X.Y and X.Y) reviewed photographs using a checklist. We returned personalized feedback within 3 business days to reinforce correct use or guide corrective action for the most serious error observed, drawing from a message library written in the spirit of MI. Only intervention recipients who were nonadherent at 6-month follow-up were eligible for rerandomization in phase 2 of the SMART design.

#### EUC

We distributed a study-developed electronic information sheet about Illinois child passenger safety law. We sent text message photograph requests and follow-up activity reminders. We provided corrective feedback only for predefined critical errors. We granted access to the Car Seat Compass for 1 month after study completion.

### Phase 2

#### High-Intensity TCBD/ABCB

We scheduled a second brief, remote MI session, to occur within 6 weeks of rerandomization and programmed 3 tailored text messages each month. We continued to provide personalized feedback on submitted photographs.

#### Low-Intensity TCBD/ABCB

We maintained Car Seat Compass access and sent 2 tailored text messages per month. We also returned personalized photograph feedback.

### Data Collection

In March 2020, after enrolling the first 9 participants, study activities paused due to COVID-19 restrictions. In June 2020, we resumed an adapted protocol with fully remote study activities (see Protocol documents in Supplement 1). Recruitment was lower than anticipated because the COVID-19 pandemic suppressed pediatric acute care visits. We stopped recruitment in September 2022, before reaching intended recruitment targets,^27^ to ensure completion of 12-month assessments within the funding period.

We collected data via online surveys. The screener was programmed in Qualtrics XM (Qualtrics; version accessed from February 2020 to August 2022) and the baseline, 6-month, and 12-month assessments were conducted using the Michigan Tailoring System.^31^ Data were also collected via text messages (photographs) and brief phone or video chat interviews (6- and 12-month assessments). We used interview data, when available, to determine usual CRS; otherwise, we relied on survey responses. Because of COVID-19 study modifications, we completed no in-person assessments. Eleven photograph requests were programmed and sent during caregiver-approved, but unscheduled, time windows (ie, caregivers did not know when requests would be sent). Caregivers submitted views of their child as they usually travel in a vehicle. Caregivers received electronic gift card incentives for survey completion ($20 baseline, $30 at 6 months, and $45 at 12 months) and photograph submissions ($5 per submission plus up to two $5 bonuses for streaks of 4 submissions).

In the baseline survey, caregivers provided demographic information, including their self-reported race and ethnicity and the race and ethnicity of their child. Response categories included Black, not Hispanic or Latine; Hispanic or Latine, any racial identity; White, not Hispanic or Latine, and multiple or other races, not Hispanic or Latine (defined as American Indian or Alaska Native, Asian, Middle Eastern or North African, Native Hawaiian or Other Pacific Islander, and any race or ethnicity not otherwise specified). We obtained baseline weight and height from the electronic health record (EHR) for children presenting to Lurie Children’s sites. Caregiver report was required for Central DuPage Hospital, which is on a separate EHR, and for 6- and 12-month assessments if there was no recent Lurie Children’s visit. We projected child size using their baseline growth percentiles from US Centers for Disease Control and Prevention growth curves^32^ if time between size measurement and study assessment was more than 1 month. When no EHR data were available and caregivers reported implausible weights or heights, we used the 3rd percentile for age for low reported values and the 97th percentile for age for high reported values. If no size data were available, we used the 50th percentile for age.

### Outcome

Our primary outcome was child passenger safety guideline adherence, a dichotomous composite measure comprised of all 3 criteria: (1) usual travel with an age- and size-appropriate CRS, (2) in the vehicle back seat, and (3) never traveling unrestrained. We defined age- and size-appropriateness using weight and height limits for typical CRS on the US market in 2024^33^ (eMethods and eTable 1 in Supplement 2). To provide current context for the weight limits used for our outcome definition, we present counts of CRS on the US market in 2025 (eTables 2-5 in Supplement 2). We determined the primary outcome for phase 1 using data from 6-month assessments and for phase 2 using data from 12-month assessments. Our secondary outcome was age- and size-appropriate CRS use alone (without considering seating location or ever traveling unrestrained).

### Potential Harm

We considered potential harms. These included possible delayed transition, and use of a CRS beyond typical CRS upper weight or height limits.

### Randomization Sequence

Random allocation sequences were generated by study biostatistician (J.G.) and programmed into the study dashboard. Initial randomization was stratified by (1) recommended CRS (rear-facing, forward-facing, or booster seat) and (2) eligibility reason (not guideline adherent or planning premature transition) in blocks of 4 to 8 in a 3:1 ratio to TCBD/ABCB or EUC. We randomized 3:1 at baseline to ensure sufficient sample size for phase 2 rerandomization. Initial allocation was revealed in the study dashboard after participants completed baseline surveys. The 3:1 balance was altered by screen failures and creation of generic EUC profiles for access to the web-application after study completion. Rerandomization of caregivers who were exposed to basic TCBD/ABCB and were nonadherent at 6 months occurred in phase 2 at a 1:1 ratio to high- or low-intensity intervention. Rerandomization was revealed in the study dashboard after the RC categorized 6-month behaviors.

### Blinding

Caregivers, RCs, and counselors engaged in MI sessions and could not be blinded to group assignment. The determination of guideline adherence was blinded by using a file that did not contain randomization group information.

### Statistical Analysis

Power calculations used a 2-sided α = .05. We sought to enroll 900 participants with 80% retention to reliably distinguish (>80% power) between 28% and 17% guideline adherence for intervention and EUC, respectively. We performed intention-to-treat analysis, first comparing 6-month guideline adherence for phase 1: basic TCBD/ABCB vs EUC. We limited 12-month outcome analyses to participants who completed 6-month assessments required to determine rerandomization eligibility. We compared 12-month guideline adherence for the SMART design among participants rerandomized in phase 2. In parallel, we assessed maintenance effects by comparing 12-month guideline adherence among basic TCBD/ABCB participants from phase 1 who were guideline adherent at 6 months vs EUC.

We used unadjusted logistic regression (glm logit models) and logistic regression adjusted for 3 a priori covariates: randomization strata (accounting for child age and weight), caregiver gender, and caregiver race and ethnicity for the 6-month outcome, the 12-month SMART outcome, and 12-month maintenance effects. Caregiver race and ethnicity was a planned covariate given historically observed differences in CRS use.^18,20,34,35,36^ In the eMethods in Supplement 2, we provide additional details of the analyses within the context of the SMART design for primary and secondary outcomes as well as a sensitivity analysis considering participants who withdrew and were lost to follow-up as not guideline adherent at 6- and 12-month outcome assessments.

To produce more easily interpreted covariate effects, we present absolute change in likelihood of treatment response by casting the estimates from the logistic regression models as marginal effects using the margins command in Stata 18.0 (StataCorp). Thus, covariate effects are presented as absolute percentage differences. Odds ratios from these models and analyses considering participants without outcome data as not guideline adherent are presented in eTables 6 to 10 in Supplement 2. We graphed guideline adherence across intervention groups stratified by the CRS recommended at baseline. We describe possible delayed transitions by intervention group. We tested for interaction effects between the randomization strata and intervention group and accounted for children who crossed a weight or height threshold during the study (eMethods in Supplement 2). All analyses were conducted from October 2024 to March 2025 in Stata. *P* values <.05 were considered statistically significant.

## Results

### Baseline Data

**Figure 1.**
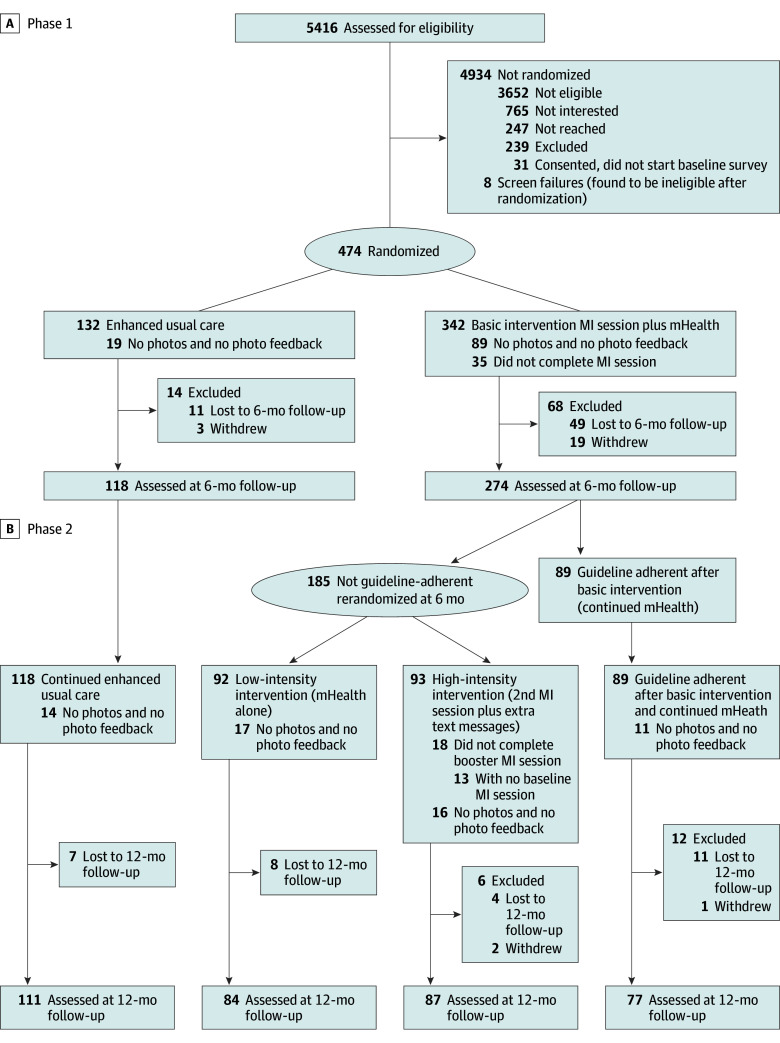
Enrollment, Randomization, and Follow-Up of Participants in the Trial mHealth indicates mobile health; MI, motivational interviewing.

Participant flow is depicted in Figure 1. Screening survey invitations were sent via email, text, and EHR patient portal message to families following 48 235 Lurie Children’s ED and urgent care visits. Screening was completed by 5415 caregivers (11.2%), of whom 1282 were potentially eligible. Of these 765 (59.7%) were not interested, 247 (18.3%) were not reached, and 513 (40.0%) were enrolled. Of the enrolled population, 31 did not complete baseline surveys and 8 were deemed ineligible during their MI session, leaving 474 participants randomized in phase 1: 342 to basic TCBD/ABCB (mean [SD] caregiver age, 36.1 [6.2] years) and 132 to EUC (mean [SD] caregiver age, 35.7 [6.5] years). Median (IQR) duration for baseline MI sessions was 15 (11-18) minutes. Six-month assessments were completed by 274 TCBD/ABCB participants (80.1%) and 118 EUC participants (89.4%) and were included in the phase 1 outcome analysis.

Nearly two-thirds of participants who received basic TCBD/ABCB (185 of 274 participants [67.5%]) were not guideline adherent at 6 months and were rerandomized in phase 2: 93 to high-intensity TCBD/ABCB (second MI plus extra text messages) and 92 to low-intensity TCBD/ABCB (mHealth alone). Median (IQR) duration for second MI sessions was 10 (8-12) minutes. Twelve-month assessments were completed by 359 of 392 participants (91.6%) who had 6-month assessments, 77 of 89 participants (86.5%) who were guideline adherent after basic TCBD/ABCB, 111 of 118 participants (94.1%) who received EUC, 87 of 93 participants (93.5%) rerandomized to high-intensity TCBD/ABCB, and 84 of 92 participants (91.3%) rerandomized to low-intensity TCBD/ABCB. There were 14 participants who were not rerandomized but found at outcome assessment to not be using the appropriate CRS

Study population characteristics are presented in Table 1. Of the 474 caregivers, 443 (93.5%) were mothers; 264 (55.7%) were 30 to 39 years old; 52 (11.0%) preferred Spanish; 65 (13.7%) self-identified as Black, not Hispanic or Latine; 188 (39.7%) as Hispanic or Latine, any racial identity; 177 (37.3%) as White, not Hispanic or Latine; and 44 (9.3%) as multiple or other races, not Hispanic or Latine (including 1 American Indian and Alaska Native caregiver, 25 Asian caregivers, 3 Middle Eastern or North African caregivers, and 15 caregivers who identified with multiple racial groups). Of the children, 194 (40.9%) were female, 198 (41.8%) were aged 6 months to less than 3 years, 97 (20.5%) were aged 3 to younger than 5 years, and 179 (37.8%) were aged 5 to less than 11 years. Most participants were not guideline adherent at baseline, inclusive of prior front seat and unrestrained travel (247 of 342 participants [72.2%] receiving basic TCBD/ABCB and 96 of 132 participants [71.2%] receiving EUC); the remainder were planning a premature transition. Front seat travel was reported by 20 caregivers, 15 of whom reported using a nonrecommended CRS. Unrestrained travel was reported by 46 caregivers, 20 of whom reported using a nonrecommended CRS. Characteristics of the 185 participants who were rerandomized at 6-month are presented in Table 2. One family receiving EUC and 4 families receiving high-intensity TCBC/ABCB received study-provided CRSs after expressing financial need.

**Table 1.** Study Population Characteristics

Characteristics	Participants, No. (%) (N = 474)	
	Intervention (n = 342)	Enhanced usual care (n = 132)
Caregiver relationship		
Mother	322 (94.1)	121(91.7)
Father	20 (5.9)	10 (7.6)
Grandparent	0	1 (0.8)
Caregiver age, mean (SD), y	36.1 (6.2)	35.7 (6.5)
Language preference		
English	303 (88.6)	119 (90.2)
Spanish	39 (11.4)	13 (9.9)
Caregiver race and ethnicity (self-reported)		
Black, not Hispanic or Latine	53 (15.5)	12 (9.1)
Hispanic or Latine, any racial identity	128 (37.5)	60 (45.5)
White, not Hispanic or Latine	136 (39.8)	41 (31.1)
Multiple or other races, not Hispanic or Latine^a^	25 (7.3)	19 (14.4)
Highest level of education		
High school or less	76 (22.2)	33 (25.0)
Technical school or associate’s degree	56 (16.4)	20 (15.2)
Bachelor’s degree	85 (24.9)	43 (32.6)
Graduate or professional school	125 (36.6)	36 (27.3)
Lives with other parent	259 (76.2)	103 (79.2)
Household income, $		
<25 000	60 (17.5)	25 (18.9)
25 000-49 999	69 (20.2)	26 (19.7)
50 000-149 999	92 (26.9)	40 (30.3)
≥150 000	104 (30.4)	38 (28.8)
Do not know	17 (5.0)	3 (2.3)
Family receives food assistance	117 (34.3)	43 (32.6)
Child has Medicaid coverage	157 (45.9)	57 (43.2)
Child age		
6 mo to <36 mo	140 (40.9)	58 (43.9)
3 y to <5 y	72 (21.1)	25 (18.9)
5 y to <11 y	130 (38.0)	49 (37.1)
Child sex		
Male	202 (59.1)	78 (59.1)
Female	140 (40.9)	54 (40.9)
Child race and ethnicity (caregiver reported)		
Black, not Hispanic or Latine	50 (14.6)	11 (8.3)
Hispanic or Latine, any racial identity	141 (41.2)	60 (45.5)
White, not Hispanic or Latine	117 (34.2)	37 (28.0)
Multiple or other races, not Hispanic or Latine^b^	34 (9.9)	24 (18.2)
No. of children in family		
1	96 (28.1)	36 (27.3)
2	132 (38.6)	50 (37.9)
3	86 (25.2)	33 (25.0)
≥4	28 (8.2)	13 (9.8)
Vehicle type^c^		
Car	112 (32.8)	40 (30.3)
Truck	22 (6.4)	8 (6.1)
Van	13 (3.8)	6 (4.6)
Sport utility vehicle	168 (49.1)	65 (49.2)
Minivan	27 (7.9)	13 (9.9)
Initial randomization strata		
Rear-facing recommended, not guideline adherent	79 (23.1)	36 (27.3)
Rear-facing recommended, plans premature transition	48 (14.0)	18 (13.6)
Forward-facing recommended, not guideline adherent	116 (33.9)	40 (30.3)
Forward-facing recommended, plans premature transition	37 (10.8)	15 (11.4)
Booster seat recommended, not guideline adherent	52 (15.2)	18 (13.6)
Booster seat recommended, plans premature transition	10 (2.9)	5 (3.8)

^a^ Caregiver other race includes American Indian or Alaska Native (1 participant), Asian (25 participants), Middle Eastern or North African (3 participants), and 15 caregivers identified with multiple racial groups. No caregivers identified as Native Hawaiian or other Pacific Islander.

^b^ Child other race includes American Indian or Alaska Native (2 participants), Asian (17 participants), Middle Eastern or North African (3 participants), and other (defined as any race or ethnicity not otherwise specified [7 participants]), and 29 children identified with multiple racial groups. No children identified as Native Hawaiian or Other Pacific Islander.

^c^ Two participants had a vehicle with no back seat; neither were using the recommended child restraint system for their child’s age and weight.

**Table 2.** Phase 2 Rerandomized Population Characteristics

Characteristics	Intervention, No. (%) (N = 185)	
	High-intensity (n = 92)	Low-intensity (n = 93)
Caregiver relationship		
Mother	89 (95.7)	85 (92.4)
Father	4 (4.3)	7 (7.6)
Caregiver age, mean (SD), y	34.7 (6.46)	36.8 (5.96)
Language preference		
English	82 (88.2)	78 (84.8)
Spanish	11 (11.8)	14 (15.2)
Caregiver race and ethnicity (self-reported)		
Black, not Hispanic or Latine	12 (12.9)	17 (18.5)
Hispanic or Latine, any racial identity	32 (34.4)	37 (40.2)
White, not Hispanic or Latine	41 (44.1)	32 (34.8)
Multiple or other races, not Hispanic or Latine^a^	8 (8.6)	6 (6.5)
Highest level of education		
High school or less	14 (15.1)	25 (26.1)
Technical school or associate’s degree	16 (17.2)	13 (14.1)
Bachelor’s degree	26 (27.7)	20 (21.7)
Graduate or professional school	37 (39.8)	35 (38.0)
Lives with other parent	73 (78.5)	69 (76.7)
Household income, $		
<25 000	12 (12.9)	21 (22.8)
25 000-49 999	16 (17.2)	17 (18.5)
50 000-149 999	29 (31.2)	28 (30.4)
≥150 000	30 (32.3)	22 (23.9)
Do not know	6 (6.5)	4 (4.4)
Family receives food assistance	28 (30.1)	36 (39.1)
Child has Medicaid coverage	38 (40.9)	50 (54.3)
Child age		
6 mo to <36 mo	39 (41.9)	38 (41.3)
3 y to <5 y	29 (21.5)	14 (15.2)
5 y to <11 y	34 (36.6)	40 (43.5)
Child sex		
Boy	57 (61.3)	58 (63.0)
Girl	36 (38.7)	34 (37.0)
Child race and ethnicity (caregiver reported)		
Black, not Hispanic or Latine	11 (11.8)	16 (17.4)
Hispanic or Latine, any racial identity	38 (40.9)	37 (40.2)
White, not Hispanic or Latine	35 (37.6)	33 (35.9)
Multiple or other races, not Hispanic or Latine^a^	9 (9.7)	6 (6.5)
No. of children in family		
1	27 (29.0)	22 (23.9)
2	37 (39.8)	37 (40.2)
3	21 (22.6)	25 (27.2)
≥4	8 (8.6)	8 (8.7)
Vehicle type		
Car	19 (20.4)	34 (37.0)
Truck	10 (10.7)	6 (6.5)
Van	5 (5.4)	3 (3.3)
Sport utility vehicle	50 (53.8)	42 (45.7)
Minivan	9 (9.7)	7 (7.6)
Initial randomization strata		
Rear-facing recommended, not guideline adherent	27 (29.0)	26 (28.3)
Rear-facing recommended, plans premature transition	12 (12.9)	9 (9.8)
Forward-facing recommended, not guideline adherent	36 (38.7)	33 (35.9)
Forward-facing recommended, plans premature transition	5 (5.4)	7 (7.6)
Booster seat recommended, not guideline adherent	11 (11.8)	15 (16.3)
Booster seat recommended, plans premature transition	2 (2.2)	2 (2.2)

^a^ Other race includes American Indian or Alaska Native (1 participant), Asian (5 participants), and Middle Eastern or North African (2 participants), and 6 caregivers identified with multiple racial groups. No caregivers identified as Native Hawaiian or other Pacific Islander.

### Outcomes and Estimation

**Figure 2.**
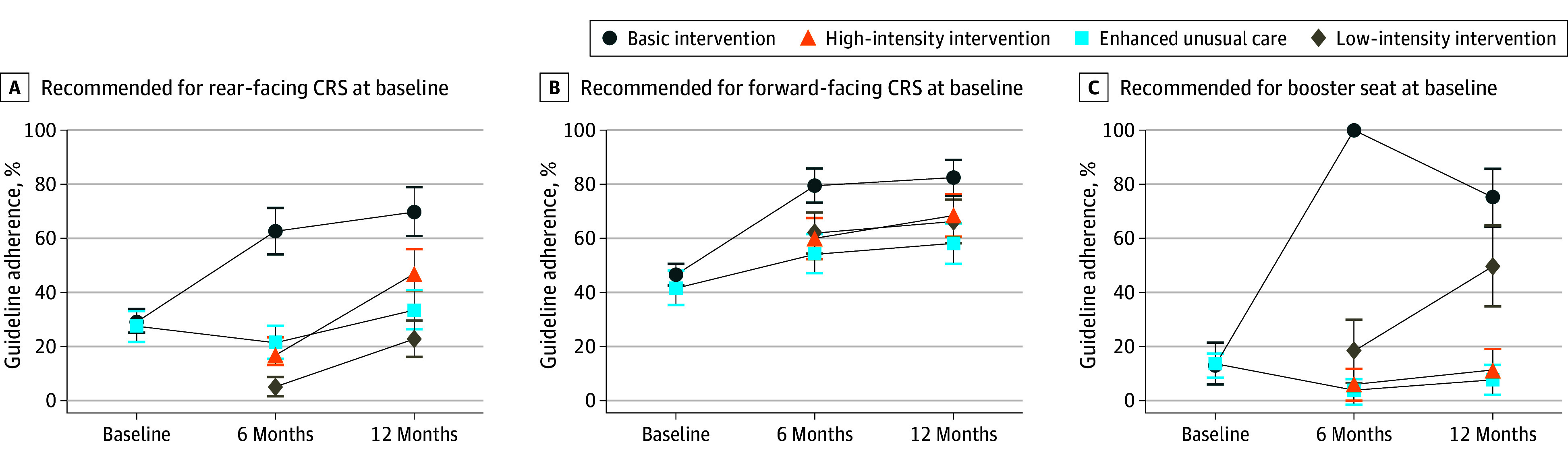
Adjusted Percent Guideline Adherence Across Intervention Groups by Recommended Child Restraint System (CRS) at Baseline Graphs show the percentage of guideline adherence with standard errors, adjusted for randomization strata, caregiver gender, and caregiver race and ethnicity. Basic intervention included a motivational interviewing session plus mobile health. High-intensity intervention included a second motivational interviewing session and extra mobile health. Low-intensity intervention included continued mobile health.

At 6 months, 131 of 278 caregivers receiving basic TCDB/ABCB (47.1%; 95% CI, 41.3% to 53.0%) and 38 of 118 caregivers receiving EUC (32.2%; 95% CI, 23.8% to 40.6%) were guideline adherent. When accounting for randomization strata, caregiver gender, and caregiver race and ethnicity, those who received basic TCBD/ABCB had a 13.1% (95% CI, 3.6% to 22.6%; *P* = .007) greater absolute change in treatment response in phase 1 compared with EUC. At 12 months, there was a significant adjusted maintenance effect among caregivers receiving basic TCBD/ABCB who were guideline adherent at 6 months, with a 39.2% (95% CI, 26.5% to 51.9%; *P* < .001) greater absolute change vs EUC. In phase 2 of the SMART design, there was no benefit of high-intensity vs low-intensity TCBD/ABCB (−3.9%; 95% CI, −17.9% to 10.1% adjusted prevalence difference; *P* = .59) among those exposed to basic TCBD and not guideline adherent at 6 months. Unadjusted and adjusted results with marginal effects are presented in Table 3. Odds ratios are provided in eTables 6 to 8 in Supplement 2. Figure 2 presents guideline adherence by recommended CRS and intervention groups across the study period. eTable 9 in Supplement 2 presents results of our secondary outcome, age- and size-appropriate CRS use alone. eTable 10 in Supplement 2 provides results of our sensitivity analysis, examining participants who withdrew or were lost to follow-up as not being guideline adherent at 6- and 12-month outcome assessments.

**Table 3.** Child Passenger Safety Guideline Adherence at 6- and 12-Month Follow-Up

Intervention group follow-up time frame	Unadjusted			Adjusted^a^		
	Guideline adherent, % (95% CI)	Marginal effects, % difference (95% CI)	*P* value	Guideline adherent, % (95% CI)	Marginal effects, % difference (95% CI)	*P* value
**6-mo Outcome**						
Enhanced usual care	32.2 (23.8 to 40.6)	Reference	NA	33.4 (25.6 to 41.3)	Reference	NA
Basic TCBD/ABCB intervention^b^	47.1 (41.3 to 53.0)	14.9 (4.6 to 25.2)	.004	46.5 (41.3 to 51.7)	13.1 (3.6 to 22.6)	.007
**12-mo SMART outcomes after rerandomization in phase 2**						
Low-intensity intervention^c^	47.6 (36.9 to 58.3)	Reference	NA	48.2 (38.3 to 58.1)	Reference	NA
High-intensity intervention^d^	44.8 (34.4 to 55.3)	−2.8 (−17.7 to 12.2)	.71	44.3 (34.6 to 54.0)	−3.9 (−17.9 to 10.1)	.59
**12-mo Maintenance effects for groups not rerandomized**						
Enhanced usual care	38.7 (29.7 to 47.8)	Reference	NA	38.5 (30.0 to 47.1)	Reference	NA
Basic TCBD/ABCB intervention^e^	76.6 (67.2 to 86.1)	37.9 (24.8 to 51.0)	<.001	77.8 (68.9 to 86.6)	39.2 (26.5 to 51.9)	<.001

Abbreviations: NA, not applicable; SMART, sequential, multiple-assignment, randomized trial; TCBD/ABCB, Tiny Cargo, Big Deal; Abróchame Bien, Cuídame Bien.

^a^ Adjusted for the following covariates: randomization strata, caregiver gender, and caregiver race and ethnicity.

^b^ Motivational interviewing session plus mobile health.

^c^ Continued mobile health.

^d^ Second motivational interviewing session and extra mobile health.

^e^ Adherent at 6 mos; continued mobile health.

### Potential Harm

At 6 months, 7 (2.5%) phase 1 basic TCBD/ABCB and 2 (1.7%) EUC group participants had possible delayed transitions. At 12 months, 3 of 77 caregivers (3.9%) who were guideline adherent after basic TCBD/ABCB, 1 of 118 caregivers (0.9%) who received phase 1 EUC, 4 of 93 caregivers (4.6%) rerandomized in phase 2 to high-intensity TCBD/ABCB, and 2 of 92 caregivers (2.4%) rerandomized to low-intensity TCBD/ABCB had possible delayed transitions.

## Discussion

This SMART study found that TCBD/ABCB intervention yielded more guideline adherent child passenger safety behaviors than EUC. Maintenance effects were demonstrated at 12 months for the guideline adherent group who were not rerandomized at 6 months yet continued to receive supportive text messages and photograph feedback. Our phase 2 hypothesis, that a second MI session plus extra text messages (high-intensity intervention) would result in behavior change among caregivers who were not guideline adherent at 6 months, was not supported. Although recruitment was slower than anticipated, and we did not reach planned recruitment targets, sample size is unlikely the explanation for phase 2 null effects given that the raw rates showed no difference. Our findings suggest that there was a group of caregivers primed for early and lasting child passenger safety behavior change. We also found a group of caregivers who were resistant to adopting child passenger safety guidelines. For these caregivers, a more intensive MI intervention or different intervention modalities may be needed.

TCBD/ABCB is unique in its use of tailored MI-based and mHealth approaches, its longitudinal nature, and inclusion of children across CRS modes. Effects of prior child passenger safety interventions have been mixed.^37^ Our 6-month findings align with an ED-based single MI session for seat belt use in adults.^38,39^ Other ED-based mHealth interventions have improved CRS knowledge and behaviors^40,41^ and young adult seat belt use.^42^ Most prior child passenger safety interventions have used a single point of education within community and clinical settings, some including CRS distribution.^40,43,44,45,46,47,48^ Few studies have tracked knowledge or CRS use to 6 months or beyond.^40,49,50,51,52,53^ Many have narrowly focused on specific age groups and CRS modes, often children aged 4 to 8 years and booster seats,^54,55,56^ a group positively impacted by TCBD/ABCB.

While anticipatory guidance,^10^ laws,^8^ and social marketing^49,52,57^ have contributed to population-level CRS uptake, precision prevention interventions, such as TCBD/ABCB, are needed because passenger safety behaviors remain suboptimal for up to 45% of children nationally.^18,19^ Nonadherence to the behaviors targeted by TCBD/ABCB puts children at greater risk for injuries in a crash.^11,20,21,22,23,24^ The fully remote adaptations, required in response to the COVID-19 pandemic, increase TCBD/ABCB’s portability to other clinical and community contexts. Centralized telehealth models to promote health behaviors are being tested in integrated care networks.^58^ Similar health system–level implementation of TCBD/ABCB following acute and primary care visits could be advantageous because child passenger safety resources are geographically limited^59^ and virtual programs have resulted in increased CRS knowledge, confidence, and correct installation.^60,61,62,63^

### Limitations

The main limitation of our study relates to generalizability beyond a major Midwestern metropolitan area, with a robust public transit system, and outside of the COVID-19 pandemic, which likely reduced travel frequency and may have reduced participation and contributed to withdrawals. Second, while we maximized recruitment efforts by distributing screening invitations to all potentially eligible families after an ED or urgent care visit, regardless of visit type, day, and time, there is potential response bias, which may have skewed toward caregivers interested in child passenger safety or more open to behavior change. Screening rates were around 11% (5416 of 48 235 individuals) and among the 1282 respondents who appeared eligible, 765 (60%) were not interested and 247 (19%) were not reached. Third, there is potential for social desirability bias that was minimized through photograph submissions. These participants may have diluted the maintenance effect at 12 months.

## Conclusions

In this randomized clinical trial of caregivers of young children with suboptimal child passenger safety behaviors, the TCBD/ABCB intervention was associated with improved guideline adherence with lasting effects. This remote precision prevention intervention to protect against a leading cause of death may be adaptable to other clinical settings and geographic areas. Different interventions, varying in approach or dose, are needed for families resistant to changing their child passenger safety behaviors.

## Notes

Supplement 1.
Trial Protocol

Supplement 2.
eMethods.
**eTable 1.** Child Passenger Restraint System Age and Size Criteria for Dichotomous Outcome Determination **eTable 2.** Counts of Child Passenger Restraint Systems by Lower Weight Limit **eTable 3.** Counts of Child Passenger Restraint Systems by Upper Weight Limit **eTable 4.** Counts of Child Passenger Restraint Systems by Lower Height Limit **eTable 5.** Counts of Child Passenger Restraint Systems by Upper Height Limit **eTable 6.** Phase I: Unadjusted and Adjusted Odds of Child Passenger Safety Guideline Adherence at 6-Month Follow-Up **eTable 7.** Phase II: Unadjusted and Adjusted Odds of Child Passenger Safety Guideline Adherence at 12-Month Follow-Up **eTable 8.** Maintenance Effects: Unadjusted and Adjusted Odds of Child Passenger Safety Guideline Adherence at 12-Month Follow-Up **eTable 9.** Unadjusted and Adjusted Odds of Age- and Size-Appropriate Child Restraint System Use **eTable 10.** Guideline Adherence Outcome Including Participants Who Withdrew or Were Lost to Follow-Up Considering All Not Guideline Adherent

Supplement 3.
Data Sharing Statement
