# Supplementary material for: Multimodal Intervention and Child Passenger Safety Guideline Adherence in Young Children: A Sequential, Multiple-Assignment, Randomized Clinical Trial
Source: JAMA Netw Open. 2025 Sep 29;8(9):e2533912. doi: 10.1001/jamanetworkopen.2025.33912 (PMC12481228; doi:10.1001/jamanetworkopen.2025.33912)
Supplement: Supplement 1. — Trial Protocol [file jamanetwopen-e2533912-s001.pdf]

Emergency Medicine Prospective Protocol

Project Title: Tiny Cargo, Big Deal! An Adaptive ED-Based eHealth Intervention to Promote Correct and Consistent Size-Appropriate Child Passenger Safety Behaviors and Reduce Disparities

Principal Investigator: Michelle Macy, MD, MS, Attending Physician, Division of Emergency Medicine, Ann & Robert H. Lurie Children's Hospital of Chicago

Name of Federal Funding Agency: National Institutes of Health, National Institute of Child Health and Human Development (NICHD)

Version #:1. Date of approval: 11/7/2019

Background and Study Rationale

This document serves as the study's research protocol and the described study will be conducted in compliance with the provisions set forth in the protocol as well as, Good Clinical Practice standards, associated federal regulations, and all applicable Lurie research requirements.

This study will be conducted in full accordance with all applicable Lurie Children's Hospital IRB Policies and Procedures and all applicable Federal and state laws and regulations.

1 Introduction

Motor vehicle collisions (MVCs) remain the leading cause of unintentional injury deaths among children in the United States (U.S.) and racial/ethnic minority children are disproportionately impacted as suboptimal child passenger safety behaviors are more prevalent in some communities. Existing universal approaches to promote child passenger safety have fallen short of ensuring that all child passengers are correctly using size-appropriate child passenger restraints according to guidelines published by the American Academy of Pediatrics and the National Highway Traffic Safety Administration. Precision prevention programs are urgently needed to improve child passenger safety behaviors among caregivers who have not been responsive to guidelines, laws, and public education campaigns. The proposed research will test the efficacy of Tiny Cargo, Big Deal (TCBD), an emergency department (ED)-based precision prevention intervention grounded in Self-Determination Theory. TCBD integrates personalized counseling based on principles of motivational interviewing (MI) and eHealth components including a tailored educational mobile friendly website "site" and short message service (SMS) communications with the goal of improving child passenger safety. We hypothesize that by providing tailored child passenger safety education and personalized skills for restraint use in a manner that supports autonomous motivation the TCBD intervention will be more efficacious than universal approaches (laws/guidelines) for realizing correct use of size-appropriate child passenger restraints.

1.1 Background and Relevant Literature

The overarching scientific premise of the proposed study is derived from the published literature demonstrating that the emergency department (ED) can be a site of screening to identify suboptimal child passenger safety behaviors[1-3] and can serve as a site of intervention,[4-10] that tailored child passenger safety information can increase parental knowledge,[6,11] and that eHealth holds promise for child passenger safety education.[6,12-16] Additionally, our own pilot work in the ED suggests that child passenger safety behaviors can be improved and disparities reduced through a *brief counseling session* and *tailored educational brochures*. Despite this

collective evidence, suboptimal child passenger safety behaviors are prevalent and a key contributor to injuries sustained in motor vehicle collisions (MVCs).

Motor vehicle collisions (MVCs) remain a significant and costly public health problem in the United States (U.S.). MVCs are *the #1 cause* of unintentional injury-related deaths among 4-12 year olds and a leading cause of unintentional injury-related deaths among younger children.[17,18] **In fact, more U.S. children die from MVCs than die of heart disease, chronic lower respiratory diseases, influenza/pneumonia, or sepsis each year.**[18,19] Child passenger fatality rates have been stagnant around 650 per year from 2010 and 2014.[18,20] In 2015, crash fatality rates in the U.S. rose by the largest percent in 50 years.[21,22] Non-fatal injuries also represent an enormous burden. In 2013, more than 131,000 children received emergency treatment for non-fatal injuries sustained in MVCs,[23] and more than half of these children required hospital admission or transfer to another ED.[23] Childhood survivors of MCVs can experience lifelong sequelae, including mobility impairments, irreversible brain or spinal cord injuries, and post-traumatic stress disorder.[24-26]

Universal strategies[27] to promote child passenger safety provide recommendations for the population as a whole and are disseminated in national guidelines, state laws, and public education campaigns.[28-35] These strategies have reached many caregivers but **current recommendations are not adopted for all child passengers.**[36-38] Precision prevention strategies,[27] targeted to those who have not responded to universal strategies, are urgently needed to reduce unintentional injuries from MVCs.[16,39] Suboptimal child restraint system (CRS) use is lowest for infants, with about 10% prematurely using a forward-facing car seat with a harness or booster seat. Suboptimal CRS use increases considerably after a child's first birthday; roughly 30% of toddlers use a booster seat or seat belt alone and >50% of elementary school age children use a seat belt alone when they would benefit from a more protective restraint.[36-38,40] The greatest risk for injury is among children who travel unrestrained. This is permitted by caregivers in increasing proportions as children progress from toddlers (5%) to teens (15%).[38] Proportions of fatally injured unrestrained children increase from 27% among <4 year olds to 45% for 8-12 year olds.[41]

Prior child passenger safety research has generally encouraged use of car seats or booster seats for children in traditional age categories.[6,9,13,14,42-44] We propose to move beyond one-size-fits-all age-based approaches with an innovative precision prevention intervention that: 1) *targets caregivers* with suboptimal child passenger safety behavhiors; 2) integrates child size, anticipated growth trajectory, and current CRS into *personalized recommendation*s for *correct use* of a *size-appropriate restraint*; 3) *tailors messages* on caregiver race/ethnicity and Self-Determination Theory constructs (autonomy, relatedness, competence); and 4) has a second level of *increased intensity* to address caregivers who do not change their child passenger safety behavior by Month 6 of the study.

This proposal aligns with the NICHD Pediatric Trauma Research Program goal to reduce morbidity and mortality in childhood through injury prevention. The national strategy on highway safety has become focused on the goal of moving "Toward Zero Deaths" on U.S. roadways.[45] In addition, the CDC has declared **child passenger safety is a "Winnable Battle"** based on the large potential impact and known effective strategies to address transportation-related injuries.[46] Healthy People 2020 Objectives for Unintentional Injury Prevention include increasing age-appropriate child restraint system (CRS) use in children with age-group specific targets that are far from optimal due to limited adoption of current recommendations.[47]

The fact that seat belt use reduces the risk of injury is indisputable.[48] However, **vehicle seats and seat belts are designed for adults not children.**[49-51] Children experience dramatic physical and biomechanical changes before reaching the stature (around a height of 57”) and maturity necessary to achieve proper fit with an adult seat belt in a vehicle seat.[52-60] To accommodate the range of child growth and development, distinct CRS modes (rear-facing car seats, forward-facing car seats, and booster seats), have been designed to provide greater levels of protection than an adult seat belt alone can provide to a child in a crash.[61] CRS, used correctly, prevent injury and save lives. Rear-facing CRS use until age 2 lowers the odds of serious injury in a frontal crash 5-fold compared with forward facing.[62] Forward-facing car seats reduce risk of serious injury for toddlers by 70% and risk of death by 25% compared with seat belts.[48] Booster seats cut the risk of non-fatal injury among 4 to 7-year-old children in half compared with seat belts alone.[63,64] This evidence provides the foundation for the 2011 updates to child passenger safety recommendations from the American Academy of Pediatrics (AAP),[32,61] which state a child should: 1) remain rear-facing as long as possible, until at least age 2 or reaching the weight or height limits of their rear-facing CRS; 2) then use a forward-facing harness restraint until reaching the weight or height limits of that CRS; 3) then use a booster until proper seat belt fit can be achieved usually between ages 8 and 12; and 4) always ride in a back seat.

Addressing suboptimal child passenger safety in minority populations is crucial to eliminate disparities. Death rates among passengers <13 years are significantly higher for black compared with white children (0.73 vs. 0.57 per 100,000) and fatally injured black children are almost twice as likely to have been unrestrained compared with white children (45% vs. 26%).[41] In national observational data, we found suboptimal child passenger safety behaviors, including premature transitions to a less protective restraint and travelling unrestrained, were more common among minority children.[65] In survey data we obtained from caregivers in two Michigan EDs, racial differences in child passenger safety behaviors persisted after adjusting for caregiver education, income, and sources of child passenger safety information.[1]

Research shows that ED visits are a window of opportunity for behavior change. We have selected the ED as the location to initiate our intervention because ED visits are frequent among U.S. children, totaling more than 25 million annually.[66,67] In 2014, about 20% of children visited a hospital ED at least once.[68] Children who utilize the ED may have limited access to primary care services and many come from poor and minority populations.[69-71] As a result, some families in the ED have less exposure to primary care-based injury prevention programs. Public health challenges, including child passenger safety,[4,9] adult seat belt use,[5,72] smoking cessation,[73-75] substance abuse,[76-78] and violence prevention[79-81] have been addressed in the ED. Conducting our study in a community hospital ED will complement the literature from child passenger safety studies conducted in large children’s hospital EDs[6,9,11] and be more generalizable to the types of EDs that provide emergency care to most U.S. children.[82]

2 Study Objectives

The proposed randomized, adaptive trial will test the efficacy of a selective intervention, *Tiny Cargo, Big Deal* (TCBD), to improve child passenger safety among caregivers who are not adherent to current national recommendations. The TCBD intervention, grounded in Self-Determination Theory (SDT) and developed as a part of Dr. Macy’s K23 award, consisted of a brief motivational interviewing (MI) session in the ED and a mailed brochure tailored on child age, size, and restraint use. TCBD proved feasible and acceptable with *significant promise for improving child passenger safety behaviors* at 6-month follow-up. We have enriched TCBD based on pilot participant feedback to include a streamlined ED counseling session, new eHealth components, and one booster MI session delivered by phone. To replace the paper

brochures, we have designed a prototype mobile friendly web "site" that has shown positive results in usability testing. The study site will be optimized for mobile devices and provide to the intervention group with continuous access to *personalized, precise* child passenger safety information. We will augment the intervention with tailored short message service (SMS) communications (i.e., text messages) that facilitate bi-directional flow of information after the ED visit.

We hypothesize that a precision prevention intervention (TCBD) that provides tailored child passenger safety education and personalized skills for restraint use in a manner that supports autonomous motivation will be more efficacious than universal approaches (laws/national guidelines) for realizing correct use of a size-appropriate child passenger restraint. The primary outcomes, size-appropriate restraint use and the Child Passenger Safety Score (CPaSS), will be assessed with direct observation at scheduled, in-person appointments and remote observation of digital photographs submitted by caregivers in response to unscheduled text messages.

We will test our hypothesis using a randomized adaptive trial design with the following aims: **Aim 1:** To test the efficacy of the TCBD intervention compared to usual care for improving primary outcomes [i.e., size-appropriate restraint (dichotomous) and CPaSS-D (numeric)] at 6 and 12 months. **Aim 2:** To use latent growth curve models to identify characteristic trajectories for the CPaSS-R observed in digital photographs over the 12 study months and assess for correlates of the trajectory types. This innovative, theoretically grounded research will have long-standing impact on child passenger safety by informing the best methods to increase correct use of size-appropriate child restraint systems among caregivers of diverse backgrounds at the point they interact with the health care system. The work has the potential for wide-scale implementation in the ED and for translation to primary care offices, schools, and community settings with an ultimate goal of eliminating child passenger safety disparities and reducing the number of childhood injuries from MVCs.

2.1 Primary Objectives

The primary objectives of this efficacy trial are to:
1) test the efficacy of the TCBD intervention compared to usual care for improving primary outcomes [i.e., size-appropriate restraint (dichotomous) and the Child Passenger Safety Score (CPaSS) (numeric)] assessed at 12 months
2) use latent growth curve models to identify characteristic trajectories for the CPaSS observed in digital photographs submitted to the study team by caregivers over the 12 study months. Correlates of the trajectory types will be assessed

2.2 Secondary Objectives

Additionally, we will determine moderators (caregiver sex, race, child age) and mediators (Self-Determination Theory constructs of autonomy, relatedness, competence; emotional state at ED recruitment) of the intervention effects at 12-month follow-up.

Finally, we plan a cost analysis of the intervention to inform wider dissemination and implementation in settings outside of the ED. We will measure the total costs of the intervention from participant and health system perspectives by gathering estimates of intervention resource requirements, including personnel costs, implementation time, participant time, and material costs. We will calculate costs of the intervention per child. Net costs (savings) will be calculated by subtracting mean costs per participant in the control condition from mean costs per participant who receive the basic TCBD and the enhanced TCBD interventions. We will also

estimate cost-effectiveness as measured by dollars per caregiver using a size-appropriate CRS and using the CRS correctly.

3 Investigational Plan

Study Phase: Baseline Visit (study enrollment)
Study Group: All participants
Setting: Emergency Department at LCH or remote (via phone/text message)
Timing: Study procedures must be complete during ED visit or within 48 hours

- Data collected from medical chart in EPIC: Information that will be recorded from the medical record includes: MRN number, chief complaint, weight, name of child, date of birth, and age. We will also use the medical chart to make sure the child does not meet any of the exclusion criteria as specified in the protocol and study staff are able to approach the family.
- Screening survey: If a child's parent/legal guardian chooses to be screened for eligibility in the full study, they will complete a short survey (~10 min.) on a study iPad. The survey will ask about their safety behaviors for kids in and around cars. It will ask about how their child usually rides in cars too. They should answer all questions for the child that is getting care at Lurie Children's. All answers are private, meaning they will not be shared with anyone outside of the study team.
- Baseline survey: If the child's parent/legal guarding screens eligible for the full study and consents to participate, they will complete a (baseline) survey on an iPad. This will take about 15-20 minutes. The survey will have questions about their use of car seats, and thoughts on child safety in and around vehicles. If they cannot finish the survey in person, they will have 48 hours to do this survey over the phone with a staff member.
- After they get done with the survey, they will be randomly assigned (like flipping a coin) to one of two groups. They cannot pick the group that they will be in.
- The groups are:
    - Group 1 (Control --Usual Care): A study team member will give them an informational handout ---**see section 9 of IRB app "supporting document" for the PDF called, "Study Handout"**
    - Group 2 (TCBD Intervention): A study team member will give them an informational handout --**see section 9 of IRB app "supporting document" for the PDF called, "Study Handout".** They will also have about a 20-minute counseling session (in-person or over the phone) with a study team member. During this session, they will talk about how their child normally rides in their car, their goals for traveling safely with their child, and how they might reach these goals. To protect their privacy, we will do this session in a private place. We will give the parent/legal guardian the option to have other family members/friends with them in the ED be present during the counseling session or step out of the room during the session. Also, to make sure that we've recorded their answers correctly, we ask for their consent to audio tape this session. This recording will only be used for research and the audio tapes will not include info that someone could use to recognize them. They can give consent to audio tape within the informed consent.

We will also show participants randomized to the intervention group how to use the study's website called, the "Car Seat Compass" (about 10 minutes). This website will give them custom feedback based on their answers within the baseline survey. This website will give them answers to common questions about traveling with children.

Two test cases have been built out on the development website, so that the IRB is able to log in and review content that may be similar to two common occurrences we expect to see within the

study. To view these test cases, please follow the instructions listed below:

Test case 1 (study ID 400): Parent (Mother) who has a 12 month old child, currently using a rear-facing car seat, but plans to prematurely transition their child to a forward-facing car seat within the next 6 months. We are recommending that the child stay rear-facing until at least 2 years of age.

1. Using google chrome, go to website: http://macytcbd.miserver.it.umich.edu.
2. Log in with the email: bpollock@luriechildrens.org and the password: TCBDstudy
3. Review content within website based on this parent's situation.
4. The home page illustrates a road map of child restraints and provides the family with a green light message since the family is currently using the recommended car seat (rear-facing car seat) for that child. However, because they are planning to transition their child to a forward-facing car seat in the next 6 months, they will be eligible for the study and receive additional information on this in the "just for me" tab of website.
5. The tab labeled "just for me" surfaces information based on that parent's specific responses to the baseline survey and goes into more detail about the recommended car seat for the child of the caregiver.

Test case 2 (Study ID 300): Parent (Father) who has a 6 year old child, currently using a seat belt to restrain child in vehicle. We are recommending that they child is restrained with a booster seat.

1. Using google chrome, go to website: http://macytcbd.miserver.it.umich.edu.
2. Log in with the email: bethpollock822@gmail.com and the password: TCBDstudy
3. Review content within website based on this parent's situation
4. The home page illustrates a road map of child restraints and for this family, provides a caution (yellow light message) since the family is using a non-recommended child restraint for the child. You will see the booster seat is highlighted in green to illustrate the recommended restraint for this family.
5. The tab labeled "just for me" surfaces information based on that parent's specific responses to the baseline survey and goes into more detail about the recommended car seat for the child of the caregiver.

During the baseline visit or within 48 hours, both groups (control and intervention) will also:

- Fill out a form that'll be used to get in touch with them for future follow-up visits --**see section 9 of IRB app "supporting document" for a document called, "Study Locator Form"**
- Complete a Tiny Cargo check-in. During this check-in, a study staff member will view how their child normally rides within their car. If they cannot do this check-in in-person, they will have 48 hours to send us photos. These photos will show us a few different views of their child as they normally ride in their car.
- ****See section 9 of IRB app, "supporting documents" for a document called, "TCBD text library and error messages" and refer to the sheet labeled, "Response to errors."** This document provides scripted responses that the RA will use when major misuse and/or errors are visible during the Tiny Cargo check-ins. The TCBD intervention

messages are referred to as "Scripted MI response" while the control group's responses are referred to as "scripted control response."

Study Phase: Monthly Photo Requests (During Months 1-5 and Months 7-11)
Study Group: All participants
Setting: Remote (text message via caregiver's mobile device)
Timing: Study procedures must be complete within each month (within 4 weeks)

Monthly photo requests**:**

- ****See section 9 of IRB app, "supporting documents" for a document called, "Tips & tricks for taking your monthly photo"** --this document will be provided to all participants enrolled in part 2 of the study. The RA will review this document during the ED baseline visit with the participant.
- No matter which group caregivers are assigned into at the baseline visit, we will contact the study participant again starting in 1 month. A member of our study team will send them a photo request via text message. This text message will ask them to share photos of their child as they normally ride in their car.
- These photo requests will be sent to their best phone number. All photos of their child will be sent back to us by the parent/legal guardian.
- They will get requests to submit photos a total of 8 times while taking part in this study (over the next 12 months). We will be asked for these photos during months 1- 5, and Months 7-11 of the study.
- They will get these photo requests only on the days and during the times that they tell us they are able to get messages from us. To update the days and times that they are free to be contacted, caregivers can reach out to our team at by phone or email.
- The participant will have a 4 hour window of time to submit their photo requests each month with an additional 4 hour grace period. If a participant submits their photos during their window of time each month and the photos meet the necessary requirements (meaning the RA is able to visibly see all elements of the child sitting in the car seat, then the photos will be approved and the participant will earn $5 that month. Participants can earn a total of $40 if they complete all 8 photo requests during their specified window each month and the photos are approved. If the participant completes 4 months of photo submissions in a row, then they will hit a "streak" and will earn an additional $5. They can hit a "streak" up to 2 times over the course of the study, so they can earn up to an additional $10.
- Photo message response time (evaluate and response) from study staff will be 24-48 hours. Photo requests will not be sent if the staff are not available to respond during that time window.
- For study data collection purposes, after the research team receives photos back from the study participant, the research team will use the Child Passenger Safety Score (CPaSS) to access for correct use of the child's restraint system. See below for a document called "CPaSS data elements for IRB submission" to view the tool that will be used to access for visible errors/misuse that may be seen by the study team.
- If major misuse or errors are visible within a participant's photo submissions, they will receive a scripted text message from the study team within 24-48 hours after the photo is submitted. Participants in the TCBD intervention group will receive messages in the spirit of Motivational Interviewing style while the control group will receive similar messages without the spirit of MI.
- ****See section 9 of IRB app, "supporting documents" for a document called, "TCBD text library and error messages" and refer to the sheet labeled, "Response to errors."** This document provides scripted text messages that the RA will use when major misuse and/or errors are visible within the photo submissions each month. The TCBD intervention messages

are referred to as "MI spirit text messages" while the control group's text messages are referred to as "control text messages."

Study Phase: Study Text Messages (During Months 1-12)
Study Group: All participants
Setting: Remote (text messages via caregiver's mobile device)
Timing: Study procedures will occur each month

<u>Study text messages</u>:
No matter which group caregivers are randomly assigned to, all study subjects will receive monthly text messages from our team while they are taking part in the study (12 months).

- Group 1 (control): They will get about 3-4 text messages each month. Messages will include:
  - Reminders for upcoming follow-up visits
  - Feedback related to the photos they send us of their child riding in their car
  - General study info

- Group 2 (TCBD intervention): They will get about 5-6 custom text messages. Messages will include:
  - Reminders for upcoming follow-up visits
  - Feedback related to the photos they send us of their child riding in their car
  - Tailored text messages --Custom tips and info based on their answers to study surveys
- <u>****See section 9 of IRB app, "supporting documents" for a document called, "TCBD library and error messages" and refer to the sheet labeled, "general message library."**</u> This document provides a library of custom tips/general supportive and informational text messages that the TCBD intervention group may receive over the course of the study (12 months). Participants will receive these text messages as applicable based on their answers within the surveys, based on the time of year as well as priority of message content.

Study Phase: Follow-ups --Month 6 and Month 12
Study Group: All participants
Setting: In-person or remote (via photo submission/video chat)
Timing: Within 1 month

<u>6- and 12-month follow-up visits</u>:

- All subjects will be asked to complete a 6- and 12-month (follow-up) visit.
- The follow-up visits include:
  - Taking an <u>online follow-up survey (about 20-25 minutes)</u> --the 6 month follow-up survey will also include a health literacy assessment called the Newest Vital Sign. These questions will not be asked during the 12 month survey.
  - Completing an in-person <u>Tiny Cargo check-in (about 10 minutes)</u>
  - <u>At the 6 month follow-up ONLY: Receiving a study handout</u> on the car seat recommendations for children as put out by the National Highway Traffic Safety Administration (NHTSA) **--see section 9 of IRB app for a copy of the handout called, "NHTSA child passenger safety overview"**
- Survey: The follow-up survey can be done in person on an iPad, on the study participant’s own device, or over the phone with a study team member. A team member will send them a link to take the survey so that they can do it before the visit. The follow-up survey will have questions that are like those asked on the baseline survey.

- Tiny Cargo Check-in: During the check-in, a study team member will look to see how their child usually rides while in their car. Every effort will be made to meet with the study participant and their child in person for the 6- and 12-month follow-up visit. This meeting can take place at Lurie Children's or at another location that is closer to them. If they are unable to meet with us in person (e.g., they move out of state), they may also send us photos of their child as they usually ride in their car via text message or via a video chat (e.g., Facetime or google hangout).
- ****See section 9 of IRB app, "supporting documents" for a document called, "TCBD text library and error messages" and refer to the sheet labeled, "Response to errors." T**his document provides scripted responses that the RA will use when major misuse and/or errors are visible during the Tiny Cargo check-ins. The TCBD intervention messages are referred to as "Scripted MI response" while the control group's responses are referred to as "scripted control response.
- After the 6-month follow-up assessment, caregivers in the intervention group only who continue suboptimal CRS use will be re-randomized to receive either the basic or enhanced intervention.
- The enhanced TCBD intervention group will include one additional motivational interviewing (MI) session by phone in Month 7/8 and one to two additional tailored text messages in Months 7-12 (total of 7 tailored tips during these months compared to 4 tailored tips for those in the basic TCBD). The extra text messages will be sent to those in the enhanced TCBD group until size-appropriate restraint use is observed. Caregivers receiving enhanced TCBD will be encouraged to obtain a free seat check with a certified child passenger safety technician (CPST) in the community.
- The basic TCBD intervention group will continue on in study without any changes.

Study Phase: Phone Counseling Session --Month 7/8
Study Group: Enhanced TCBD Intervention Group
Setting: In-person or remote (via photo submission/video chat)
Timing: Within 1 month

Phone counseling session (enhanced TCBD intervention):

After the study subject completes their 6-month follow-up visit, the study team will determine if the child is using the recommended child restraint system (car seat or booster seat) for their age and size. The intervention group subjects whose children are NOT using the recommended restraint will be randomly assigned (like flipping a coin) to one of two groups. They cannot pick the group they will be in.

The groups are:

- Group 2 (basic TCBD): They will keep taking part in the study with the basic intervention and nothing will change.
- Group 3 (enhanced TCBD): They will keep taking part in the intervention arm of the study but will be asked to talk with a Car Seat Coach (i.e., a behavioral research assistant trained for the study) by phone for about 20 minutes. The phone call will cover some of the challenges and successes they are having with traveling safely with their child. They will also get 1 or 2 additional study text messages each month. These extra text messages will include:
    - Feedback related to the photos they send us of their child riding in their car
    - Feedback related to survey answers

3.1 General Design

The Tiny Cargo, Big Deal! (TCBD) emergency department (ED)-based intervention is designed to promote correct and consistent use of a size-appropriate child passenger restraint system on every trip. We will test the precision prevention intervention with a randomized, adaptive trial design. Caregivers (parents and legal guardians) of children who seek care for non-critical illness and injury at the Ann & Robert H. Lurie Children's Hospital of Chicago Emergency Department will be screened. Eligible caregivers will have a child 6-months to 10-years-old and <55 inches tall who screen positive for suboptimal child passenger safety behavior or plans for premature transition to a less protective restraint in the coming year. Caregivers who agree to participate will be randomized to the basic TCBD intervention (an ED counseling session plus eHealth after ED discharge) or the control condition (usual ED care with discharge instructions relevant to the child's diagnosis and a car seat information sheet). At 6-months we will re-randomize caregivers in the intervention group alone who are not using a size-appropriate CRS to either continue basic TCBD or receive the enhanced TCBD, which includes one motivational interviewing (MI) session by phone in Month 7/8 and four instead of two text messages in Months 7-12.

3.2 Allocation to Interventional Group

After completing the baseline survey, all caregivers will be randomized into one of two groups: 1) TCBD intervention group, or 2) control group. Computerized, block randomization (block sizes from four to eight) will be applied to assign eligible caregivers to the TCBD intervention or control group in a 3:1 ratio. Blocks will be stratified by age groups related to child passenger safety recommendations (<2 years – rear-facing CRS, 2-4 years – forward-facing CRS, 5-10 years – booster seat) and the child passenger safety behavior that led to eligibility for study (not using the recommended restraint currently or planning a premature transition in the coming 6 months).

After the 6-month follow-up assessment, caregivers in the intervention group only who continue suboptimal CRS use will be re-randomized by computer in a 1:1 ratio to receive either the basic or enhanced intervention. The enhanced TCBD will include one additional motivational interviewing (MI) session by phone in Month 7/8 and one to two additional tailored text messages in Months 7-12 (total of 7 tailored tips during these months compared to 4 tailored tips for those in the basic TCBD). The extra text messages will be sent to those in the enhanced TCBD group until size-appropriate restraint use is observed. Caregivers receiving enhanced TCBD will be encouraged to obtain a free seat check with a certified child passenger safety technician (CPST) in the community. The primary outcomes (size-appropriate CRS use and CPaSS) will be assessed in-person at Months 6 and 12. The secondary outcome, the CPaSS trajectory, is a measure of correct and consistent restraint use in response to the intervention and will be assessed by scoring photographs submitted by caregivers (Months 1-5; 7-11).

<u>STUDY GROUPS:</u>

After participants complete the baseline survey, they will be randomized to either the control group (usual ED care with discharge instructions relevant to the child's diagnosis and a study handout) or the TCBD intervention group (an ED counseling session plus eHealth after ED discharge). Please also refer to section 3. D.1. for a complete outline of the study details for each group over the course of the study.

Main differences between intervention and control include the following:

At baseline visit (enrollment in study):

1) Control group -- after taking the baseline survey, they will be given a study handout that summarizes the IL law on child passenger safety and provides a few online resources for parents on child passenger safety.
2) TCBD Intervention group --after taking the baseline survey, they will receive a brief motivational interviewing inspired counseling session with the research coordinator in addition to the same study handout sheet that the control group will receive. During the MI counseling session, participants will talk to the RC about how their child normally rides in their car, their goals for traveling safely with their child, and how they might reach these goals. Additionally, the intervention group will receive direct access to the study's mobile friendly website "site," Car Seat Compass. The site will provide parents with custom feedback based on their answers within the baseline survey regarding how their child normally rides in their vehicle and their general child passenger safety practices with that child. The site will give them answers to common questions about traveling with children as well. The control group will not have access to this site throughout the study. However, the control group will obtain access to the study website after the conclusion of their 12 month follow-up.

Both groups will participant in all of the Tiny Cargo check-ins and the 6- and 12 month follow up visits scheduled over the course of the study. Those elements of the study do not change based on the group someone is enrolled in.

Monthly Photo Requests:
Both groups will be asked to send the study team photos of how their child normally rides in their vehicle and will be paid the same incentive amounts. However, if major misuse or errors are visible within the photos that participants submit, then the text messages sent to participants will vary slightly depending on which group you are enrolled in.

1) control group --they will receive a concise text message for any major misuse/errors that are visible and how to adjust for it. Non-critical errors will not elicit a direct text message response from the study team.
2) TCBD intervention group --they will receive a slightly longer text message that is framed within the spirit of motivational interviewing that addresses the major misuse/error and possible solutions to adjust for it. They will also receive tailored messages for any non-critical misuse/errors.

** See section 9 (Supporting documents) of the IRB application for an excel file that serves as a library of text messages that will be provided to participants when major misuse/errors are visible (in-person or via photos) based on the study group they are enrolled in. This is a sample of the messages that will be used and sent out, but not meant to be a comprehensive library of messages. The RC will use their discretion when reviewing misuse/errors and adjusting messages as needed.

Study Text Messages:
Both groups will receive study text messages from the study team over the course of their enrollment in the study. However, the TCBD intervention group will receive tailored text messages each month on top of the basic reminders for follow-up visits and feedback related to the photos they submit each month.

The tailored text messages that will be sent to the TCBD intervention group will be custom tips and info based on the participants answers to study surveys and the type of child restraint system they are recommended to be using. **Please see section 9 of the application to view a document called, TCBD library and error messages and refer to the sheet labeled, "general

message library." The text messages included within this sheet will be used to support the intervention group over the course of the study.

After the 6-month follow-up assessment:
Participants in the intervention group only who continue sub-optimal child restraint system use (not using the recommended seat) will be re-randomized to one of two groups:
2) TCBD basic intervention group
3) TCBD enhanced intervention group

The enhanced TCBD intervention group will include one additional motivational interviewing (MI) session by phone in Month 7/8 and one to two additional tailored text messages in Months 7-12 (total of 7 tailored tips during these months compared to 4 tailored tips for those in the basic TCBD). The extra text messages will be sent to those in the enhanced TCBD group until size-appropriate restraint use is observed. Caregivers receiving enhanced TCBD will be encouraged to obtain a free seat check with a certified child passenger safety technician (CPST) in the community.

The basic TCBD intervention group will continue on in study without any changes.

3.3 Study Measures

Over the course of this study, participants will complete the following surveys: screen, baseline, 6- and 12-month survey. At the 6-month follow-up, the participants will also complete a brief health literacy assessment called the Newest Vital Sign.

The selected measures for the surveys are reliable, valid, and have been pilot tested in the study population. Measures will be assessed at screening, baseline and re-assessed at the 6- and 12-month follow-up visits. Assessments will be self-administered or RC facilitated on a study iPad in the ED and at in-person follow-up assessments (Months 6 and 12). Skip patterns will reduce administration time. Our team has found baseline assessment lasting 20 minutes to be feasible and acceptable to caregivers in the ED setting during pilot testing.

Table 1 illustrates survey measures for this study along with the frequency/timing of assessments.

**Table 1: Measures**	Assessment Month(s)
**Primary Outcome**	
Size Appropriate Restraint Use (dichotomous)	6 and 12
Child Passenger Safety Score direct observation*	6 and 12
**Secondary Outcome**	
Child Passenger Safety Score remote observation	ED through 12
**Exploratory Cost Measures**	
Intervention Time (ED, Phone MI) for RA/Counselor	ED and 2
Intervention Time (ED, Phone MI, texts, web app) for caregiver	ED through 12
Material Costs - iPads, computers, maintenance	ED through 12
Intervention Costs - Photograph review, texts, web app	ED through 12
**MODERATORS/MEDIATORS**	
Caregiver Demographic Characteristics, reason for ED visit	ED
Child Size: Measured weight and length/height	ED, 6, and 12
Self-Determination Theory Constructs	
Treatment Self-Regulation Questionnaire[158-160]	ED, 6, and 12
Confident rulers and Perceived Competence Scale[161]	ED, 6, and 12
Health Care Climate Questionnaire[162]	12 months
Health Literacy and Numeracy	
The Newest Vital Sign[163,164]	6 months
Caregiver Emotional State, Stress, Coping	
State-Trait Anxiety Inventory[165]	ED
Risk Behavior Diagnosis Scale[166]	ED
Parenting stress index	ED
Marlowe-Crowen Social Desirability Scale[168]	6 months
Child Passenger Safety Hassles Scale[169]	ED, 6, and 12
**Footnote added 7/5/2025 for clarity in anticipation of publication of primary outcome: due to COVID-19 pandemic restrictions on in-person study activities, the project was converted to fully remote data collection. We were therefore unable to collect Child Passenger Safety Score data through direct observation.*	

3.4 Study Endpoints

3.4.1 Primary Study Endpoint

The primary outcomes (size-appropriate CRS use and CPaSS) will be assessed in-person at Month 12. The secondary outcome, the CPaSS trajectory, is a measure of correct and consistent restraint use in response to the intervention and will be assessed by scoring photographs submitted by caregivers (Months 1-5; 7-11) and collected by the study team (Months 6 and 12). Photographs will be scored using the CPaSS tool by research team members blinded to the treatment group.

4 Study Population and Duration of Participation

4.1 Duration of Study Participation

Caregivers that agree to enroll in the study will participate for up to 12 months. This includes the screening process, baseline visit, monthly photo requests, and two follow-up visits (6 and 12-months). Study participation by the caregiver/parent will end after the 12-month follow up visit.

4.2 Total Number of Subjects and Sites

To test the Tiny Cargo, Big Deal Intervention, we anticipate the need to screen 3,600 caregivers to recruit 900 caregivers who will be enrolled in the study and randomized to intervention or control. All recruitment will occur in the Emergency Department (ED) at Lurie Children’s Hospital of Chicago (LCH).

4.3 Inclusion Criteria

Caregivers (parents or legal guardians) meeting inclusion criteria will be approached to take the screening survey in the ED waiting room or ED treatment rooms during natural breaks in treatment/care. Research assistants will use the LCH ED electronic tracking board to look for participants with the following inclusion criteria to approach them to take the screening survey and will confirm upon approach that the child and family meet the following inclusion criteria:

- Child (patient) of caregiver is age 6 months to 10 years old at screening
- Child (patient) of caregiver is seeking care in the ED for a noncritical injury or illness
- Caregiver/parent is the legal guardian of the child seeking care in the ED
- Child of caregiver is less than 55 inches tall
- Caregiver speaks English or Spanish (NOTE: Spanish intervention components will be submitted for IRB review after initial IRB approval is granted. The research team will only recruit English-speaking caregivers until IRB approval is granted for the recruitment of Spanish-speaking caregivers)
- Caregiver is 18 years old or older

Caregiver/parents that complete the screening survey and meet the additional study inclusion criteria will have the opportunity to enroll in part 2 the study. The study inclusion criteria required for enrollment are:

- Caregiver owns a smart phone
- Caregiver travels at least once a week with their child in a car, truck, or van
- Caregiver screens positive for sub-optimal child passenger safety behaviors or who report plans for premature transition to a less protective restraint in the coming 6 months **(see section 9 for additional document, "screen eligibility sheet," that RCs will use to determine eligibility of caregiver for part 2 of the study based on their responses within the screening survey)**
- If enrolled, caregiver is willing to travel to follow-up locations
- If enrolled, caregiver is willing to receive and send text messages each month as part of the study
- Child of caregiver rides unrestrained more than once or twice in the past month
- Child of caregiver rides in the front seat more than once or twice in the past month

4.4 Exclusion Criteria

Caregivers (participants) will not be approached to be screened for this study if they meet any of the exclusion criteria shown below. Research coordinators will use LCH ED electronic tracking board and conversation with caregiver to identify for the following exclusion criteria:

- Caregiver does not understand/speak English (*or Spanish once IRB approval granted for recruitment of Spanish-speaking participants*)
- Child of caregiver is seeking care for a critical illness or injury: (ESI=1), treatment being given in ED trauma bay or a negative pressure room, or child requires a sepsis huddle.
- Child of caregiver is seeking treatment for a new long-term diagnosis (e.g., diabetes, cancer)
- Child of caregiver has anticipated need for admission
- Child of caregiver is seeking care for child abuse/neglect and/or Child Protective Services is involved in treatment
- Child of caregiver is seeking care that requires intensive psychosocial services (e.g., sexual assault, psychosis/active suicidal/homicidal ideation/attempt)

- Child of caregiver is seeking treatment for a motor vehicle related injury.
- Child of caregiver is too tall for study (height = >54 inches tall)
- Child of caregiver is classified as "break the glass"
- Caregiver has already refused the study twice
- Child of caregiver is already enrolled in another study at LCH (e.g., IMPROVE)
- Caregiver completed this study's screen for this child within the past 6 months
- Caregiver is already enrolled in this study for this child or another one of their children
- Caregiver is unable to provide consent (not legal guardian of child seeking care in ED)
- Caregiver is under 18 years of age
- Caregiver lives out of the state, out of the country, or outside of the central counties surrounding Chicago. (e.g., Cook, Lake, DuPage, Will)
- Caregiver is deaf, blind, or visually impaired and unable to provide informed consent

Participants (caregiver/parents) that complete the screening survey and meet the additional study exclusion criteria will also be excluded from enrolling in the study. The study exclusion criteria for enrollment are:

- Child of participant requires a wheelchair or other special needs seating when traveling in vehicles
- Participant is unwilling to travel to follow-up locations
- Participant is unwilling to receive and send text messages each month as part of the study

4.5 Subject Recruitment

Competing studies in ED:
The research study called IMPROVE (IRB #00117338) will be recruiting patients in the ED at LCH and will overlap with our study population in the following patient ages (4-10 years).

If a child comes into the ED at LCH between the ages of 4-10 years of age, and meets criteria for IMPROVE, then our study team will communicate with the Research Coordinators of IMPROVE and allow them to approach the child/family first. If the child becomes enrolled in IMPROVE, then our study team will exclude the child/family from our study and will not approach them. However, if the child is determined to be ineligible for IMPROVE and/or decides not to participate in IMPROVE, then our study team will approach the family to see if they would like to enroll.

If this plan causes any problems with the enrollment numbers for Tiny Cargo, Big Deal, then procedures will be put in place to revise this process as necessary.

Recruitment procedures:
Research Assistants will utilize the LCH ED electronic tracking board to continuously monitor incoming patients that meet study inclusion criteria during recruitment shifts. A waiver of informed consent will be requested for review of the ED Electronic Medical Record (EMR)/Patient Tracking board at LCH to determine which families to approach for screening. Adult caregivers of children 6 months to 10 years old, who bring their child to the LCH ED for any non-critical illness or injury, will be approached for eligibility screening.

All participants (caregivers/parents) meeting the basic list of inclusion criteria will be approached by an RA either in the ED waiting room or the child's (patient) ED treatment room to enroll in the part 1 screening survey. An informational handout sheet will be used by the RA to explain the study and obtain verbal consent to participant before data collection begins. Research staff will

inform the participant of the general nature of the study, their privacy rights, expectations for their participation, the voluntary nature of their participation, and that their participation can be withdrawn at any time during the course of the study. Participants will be assured that refusal to participate will not affect their child's medical care in any way. At all times, research staff will make explicit the voluntary nature of their participation. Confidentiality measures will be explained, including assurances that LCH ED staff and law enforcement will not be informed of involvement in the study and/or responses to survey items (see COC). In addition, limits to confidentiality will be explained (e.g., immediate harm to self/others). Before data collection begins, the RAs will also allow time for the participant to review the informational handout, will ask the participant if they have questions, and will ask for the participant to provide a summary of what was explained to them. Participants will be given a copy of the informational handout.

<u>Consent process:</u>
After obtaining verbal consent, participants will self-administer a computerized screening assessment survey with validated measures assessing for further study eligibility. RAs will stay in the room while the participant takes the survey to be available to assist with patient questions during screening. Study RAs will utilize procedures from prior work to seamlessly integrate the ED-based assessment into the patient’s ED stay to avoid interrupting their care.

After completing the screening survey, the RA will determine if the participant is eligible to enroll in the part 2 baseline phase of the study based on a summary of their responses. Participants that are eligible will be notified of the opportunity to take part in an additional phase of the study and the RA will use a written informed consent document to obtain consent for further study participation. This process will occur in either the ED waiting room (a private space) or within the child's ED treatment room. The informed consent includes 1) participation in the intervention study with baseline/follow-up assessments and 2) the collection of data using digital photographs. Prior to beginning any data collection with the participant, a trained research assistant will present and explain the comprehensive informed consent document, reviewing the study risks and participants’ rights. After all questions are answered to the satisfaction of the participant, and after he/she signs the consent document, then data collection will begin. Participants will be given a copy of the consent form(s).

<u>Waiver of informed consent:</u>
A waiver of informed consent will be requested for review of the LCH ED Electronic Medical Record (EMR)/Patient Tracking board, "EPIC," to determine which families to approach for screening. The ability to view LCH EPIC will be for pre-screening purposes only. The Research Assistants will utilize the LCH ED electronic tracking board to continuously monitor incoming patients that meet study inclusion criteria during recruitment shifts. Adult caregivers of children 6 months to 10 years old, who bring their child to the LCH ED for any non-critical illness or injury, will be approached for eligibility screening.

RA tracking logs will contain information regarding patients' age, gender and chief complaint. This information will help to streamline recruitment so that only eligible patients will be approached and will eliminate approaching non-eligible patients. Minimal clinical data (e.g., sex, race, age, chief complaint) will be collected on ED patients who refuse to participate in the screening survey and on ED patients who the research recruiters fail to approach for the study. Minimal identifiers of potential subjects (first name and birth date) will be kept in order to allow staff time to reconcile recruitment numbers and to avoid re-approaching patients, and this information will be destroyed as soon as possible. The research involves no more than minimal risk and does not adversely affect the rights and welfare of the patients.

This research study requires the use of the child's birth date in order to:
1) include the caregivers/parents of children between the ages of 6 months to 10 years in the study
2) determine if the child is currently using the child restraint system (CRS) that is optimal for their age according to the 2011 child passenger safety recommendations from the American Academy of Pediatrics (AAP). These recommendations provide the foundation for the educational information that is used in this study to provide caregivers/parents on which CRS to use for their child.

Safeguard against potential coercion or appearance of coercion:
The risk of potential coercion will be minimized by following standard procedures for obtaining verbal and/or written informed consent from participants. Study personnel will fully explain the study procedures, risks, benefits, and alternatives to participants. The remuneration amount was designed to balance adequate compensation for time without being too large of an amount to be coercive. Participants will also be reminded that study participation is voluntary and that refusing to participate or withdrawing from the study at any time will not result in any negative consequences on their child's care at LCH.

Participants will be allocated ample time to review the verbal informational handout and/or the written informed consent prior to data collection beginning and will be provided time to ask the Research Assistant questions as well. After all questions are answered to the satisfaction of the participant, and after he/she provides verbal consent and/or signs the consent document, then data collection will begin. Participants will be given a copy of the informational handout and/or the written informed consent form.

4.6 Vulnerable Populations:

Caregivers involved in the study may be pregnant. We will not inquire about pregnancy status. The
participation of pregnant women involves no more than minimal risk to the mother, with no foreseeable direct risks to the fetus. Children of caregivers enrolled in the study will be subjects to the extent that they will have information collected about their weight and height at ED recruitment and follow-up. In addition, images of the children will be captured in photographs of their child passenger safety behaviors, including seating location and passenger restraint system use. Identifying features of the child (e.g., face) will be obscured in the images before they are saved for analysis. The information captured in photographs is needed to determine if the child is using the size-appropriate CRS and to identify errors in CRS use. Other special vulnerable populations (including fetuses, prisoners, and institutionalized individuals) will not be subjects of the proposed research.

5 Study Procedures

5.1 Study Intervention or Observational Phase

5.1.1 Visit 1 (baseline visit)

Screening: Caregivers of children who are eligible to approach will complete a self-administered computerized screening survey on a study iPad to determine eligibility for the full study.

Caregivers who complete the screening survey and meet study eligibility requirements will have the option to enroll in the full study. Eligible caregivers will participate in the informed consent process before determining if they would like to enroll.

Eligible caregivers that enroll in the study will complete the following elements while in the ED or will have the option to finish any of these elements within 48 hours of enrollment.

- A self-administer a computerized baseline survey with validated items collecting information on their child passenger safety practices with their child, barriers/challenges they may experience in using the recommended child restraint, and motivations, etc.
- Tiny Cargo check-in (a car seat check) with the RA to directly observe and record how their child normally rides in a vehicle. We will use the CPaSS tool to collect data during the Tiny Cargo check-in.
- Randomization to the basic TCBD intervention (an ED counseling session plus eHealth after ED discharge) or the control condition (usual ED care with discharge instructions relevant to the child's diagnosis and an information sheet about car safety).
- Caregivers within the TCBD intervention group --Brief motivational interviewing inspired counseling session (~20 minutes).

5.1.2 Visit 2 –Monthly photo requests (over 12 months of study enrollment)

Over the course of the study, all study participants will receive photo requests in months 1, 2, 3, 5, 7, 8, 10, and 11 via text message from the study that asks for them to send us back digital photographs of how their child usually travels in their vehicle. Participants can complete a total of 8 photo requests over the course of the study and will be paid for completing each of them. The CPaSS measure will be used to assess all digital photographs and incorporates elements of seating position, mode selection, CRS fit, and CRS use/misuse drawn from AAP Guidelines[32] and standardized error severity scores for child restraints.[135]

5.1.3 Visit 3 -- (6 month follow-up visit)

A follow-up assessment will be administered at 6- months after the ED visit and will include both a self-administered computerized survey assessment (~15-20 minutes) and a Tiny Cargo check-in (a car seat check) administered by the RA (~10 minutes). The caregiver will have the option to complete the 6-month follow up survey in person at the visit or complete the survey on their own device (phone, iPad, laptop, etc.) prior to the follow-up visit. Allowing participants to complete the follow up assessment prior to the visit, will provide convenience to our participants and shorten the amount of time needed for the in person visit. Additionally, if participants move out of state, country or are unable to get to any of the study follow up site locations, then participants will have the option of completing the Tiny Cargo (TC) check-in (a car seat check) by submitting digital photographs to the study staff via text message or completing the check-in via video chat (e.g., skype, duo, google hangout).Caregivers that complete the TC check-in via photo submission or video chat will receive $5 less in study incentives. The CPaSS tool will be used to collect data during the TC check-ins.

During the 6-month follow-up, we will also plan to administer a brief health literacy assessment, called the Newest Vital Sign. This valid and reliable tool will help us identify participants at risk for low health literacy in the study. This survey assessment will be self-administered and will last about 3 minutes.

5.1.4 End of Study Visit – (12 month follow-up visit)

The last follow-up assessment will be administered at 12- months after the ED visit and will include both a self-administered computerized survey assessment (~15-20 minutes) and a TC check-in (a car seat check) administered by the RC (~10 minutes). The caregiver will have the option to complete the 12-month follow up survey in person at the visit or complete the survey on their own device (phone, iPad, laptop, etc.) prior to the follow-up visit. Allowing participants to

complete the follow up assessment prior to the visit, will provide convenience to our participants and shorten the amount of time needed for the in person visit. Additionally, if participants move out of state, country or are unable to get to any of the study follow up site locations, then participants will have the option of completing the TC check-in (a car seat check) by submitting digital photographs to the study staff via text message or completing the check-in via video chat (e.g., skype, duo, google hangout). Caregivers that complete the TC check-in via photo submission or video chat will receive $5 less in study incentives. The CPaSS tool will be used to collect data during the TC check-ins.

Follow-up protocol –retention of subjects

Retention will be maximized with established procedures from our prior work (80-90% compliance). The following protocol will be implemented to improve compliance with follow-up: 1) during the baseline assessment, the participant’s name, address, e-mail account, phone number, Facebook and other social media accounts, and telephone numbers of two contact persons will be obtained; 2) reminder letters, telephone calls, text messaging, e-mail communication, and/or Facebook/other social media private messaging will be used for participants who agree to be contacted by such methods; 3) follow-up interviews will be scheduled at the participant's convenience including evenings and weekends; and 4) participants will receive an extra $5.00 for confirming their follow up appointment or calling to reschedule it.

Additionally, we have set up two study e-mail accounts (TCBDstudy@luriechildrens.org and TCBDstudy@gmail.com) to be used for subjects to contact study staff or for study staff to send appointment reminders to subjects. Through the study email account, we will also set up a Facebook and Instagram limited profile page. The Facebook and Instagram profile page lists only the study name and affiliation with LCH. Facebook or Instagram will NOT be used as a recruitment tool. The account is set up only as a source for private messaging between study staff and enrolled subjects who agree to be contacted via such methods. The message shows up in the person’s private inbox, similar to an e-mail message. There is no ability to “friend” the study or post on the wall. Only subjects with active Facebook and Instagram accounts can be messaged. Privacy settings are checked on a monthly basis. When providing contact information, the subject will indicate if study staff can contact them through email, Facebook or Instagram.

5.2 Subject Withdrawal

Participants (caregivers/parents) can stop taking part in this study at any time. Their decision will not affect their regular care or their child’s care at LCH. If they wish to stop taking part in this study, we ask that participants call the study team at (224) 289-7748 or email us at TCBDstudy@luriechildrens.org. A study team member can speak to them about any concerns they may have. The study will then confirm their withdrawal from the study with a letter in the mail.

5.3 Early Termination Visits

Participants can stop taking part in this study at any time. Their decision will not affect their regular care or their child’s care. If they wish to stop taking part in this study, we ask that participants call the study team at (224) 289-7748 or email us at TCBDstudy@luriechildrens.org. A study team member can speak to them about any concerns they may have. The study will then confirm their withdrawal from the study with a letter in the mail.

6 Statistical Plan

6.1 Sample Size and Power Determination

Briefly, our calculations are based on two-sample comparisons between groups (e.g., control and TCBD) using results from our pilot study that assessed percent size-appropriate child passenger safety behaviors at 6-month follow-up (referred to as the “response rate” below). For analyses involving multiple time points, these calculations represent a conservative lower bound on the power that would only be achieved if the effective sample equals 1 observation per person (i.e. perfect within-individual correlation). For the calculations below we assume: a) 17% response rate among controls; b) 25% response rate among TCBD caregivers at 6 Month (with 75% of TCBD caregivers re-randomized); and c) only 80% of the target sample (n=204 controls and n=516 TCBD in the initial dichotomy) will be available for analysis at follow-up. Components (a) and (b) are based on pilot data where the control group received generic educational materials and outcomes were assessed at 6-months. Component (c) is a conservative correction for attrition. For Aim 2, we estimated power while accounting for uncertainty in the group sizes by simulating

response and re-randomization patterns according to the above probabilities and testing for significant between-group differences; this process was repeated 10,000 times and the power was estimated as the percent of replications in which a between-group difference was identified. Binary Outcome: For the binary response case, if the observed response rate is at least 29.4% among those receiving enhanced TCBD, it can be reliably (>80% power) distinguished from a control response rate of 17%. Analogously, a response rate of at least 27.6% among basic intervention would be reliably (>80% power) distinguished from control. Converted to Cohen’s h, both differences reflect small-to-medium effect sizes (h = 0.30 and h = 0.26, respectively). Numeric Outcome (CPaSS): If the standardized mean difference (i.e. Cohen’s D) is >0.25 (a small-to-medium effect size), we can reliably distinguish between control and basic TCBD conditions. Similarly, if D ≥ 0.28, a small-to-medium effect size, we will have sufficient power (80%) to distinguish the control and enhanced TCBD conditions. Aim 2: Monte Carlo studies of linear latent trajectory models indicate that a medium level of heterogeneity among the slopes and/or intercepts of the trajectories can be reliably recovered (power > 80%) with a total sample size of n=200, with power exceeding 95% when the level of heterogeneity is high;190 given that our sample size soundly exceeds 200, we expect sufficient ability to determine the underlying characteristic trajectory types. Nevertheless, we further justify our sample size with a brief simulation study. Assuming a final sample size of 720, conservatively assuming individuals are only measured at an average of 25% of the 12 monthly assessments, we simulate data from a population with four equally likely trajectory types: consistently low, consistently high, linearly decreasing, and starting high then dropping off. Because we have no basis for estimating variability in the score, we assumed mean/variance equivalence, consistent with the Poisson distribution. Due to the time required to fit such models, we only completed 200 simulation replications, where we were able to correctly conclude (based on BIC) that there were four trajectory types in 100% of replications, and the mean trajectories in those four groups were characterized correctly.

Exploratory Aim: Aligning with the NIH guidance on health economics research, we plan a cost analysis of the intervention to inform wider dissemination and implementation in settings outside of the ED. Unmeasured time costs for staff or for participants can be a key barrier to the implementation and long-term viability of a new intervention since preventive and screening recommendations can compete for implementation time from staff. We will address this potential barrier by measuring all relevant costs associated with the study interventions through primary data collection. We will measure the total costs of the intervention from participant and health system perspectives by gathering estimates of intervention resource requirements, including

personnel costs, implementation time, participant time, and material costs. Resource costs will include intervention development, training, and implementation. All costs will be adjusted to US 2017 dollars using the Gross Domestic Product Price Index. Control costs will be adjusted to reflect the proportion of time study participants spend on the app. We will calculate costs of the intervention per child. Net costs (savings) will be calculated by subtracting mean costs per participant in the control condition from mean costs per participant who receive the basic TCBD and the enhanced TCBD interventions. Given typical skewness, we will report means, medians, standard errors, minimum, maximum, and quantiles to provide information on cost measures. Tests will be employed to assess distributions of cost measures and identify appropriate methods for generating confidence intervals, such as non-parametric bootstrapping. We will also estimate cost-effectiveness as measured by dollars per caregiver using a size-appropriate CRS and using the CRS correctly. Economic data will provide key information about the relative costs and benefits associated with the intervention, inform scalability, and help prioritize future implementation strategies in EDs and other settings that serve young children (e.g., doctor's offices, schools).

6.2 Statistical Methods

All subjects, once randomized, will be included in an intention-to-treat analysis and outcomes will be collected even if the caregiver does not engage in the randomly assigned intervention. We will evaluate randomization by testing for covariate (e.g. demographic characteristics) balance between initial treatment groups, and between the re-randomized treatment groups created after the 6-month follow-up. If significant differences are observed, we will adjust for these variables or conduct stratified analyses. All statistical hypothesis tests and power calculations will use a two-sided = 0.05.

Aim 1: To test the efficacy of the TCBD intervention compared to usual care for improving primary outcomes [i.e., size-appropriate restraint (dichotomous) and CPaSS (numeric)] at 12 months. We hypothesize greater proportions of children will be using a size-appropriate restraint and higher CPaSS among caregivers in the intervention groups (basic and enhanced) compared with the control group (usual care) at the 12-month assessment. Statistical Analysis: Our primary analytic tool for estimating treatment effects will be generalized linear models (GLMs), and for analyses involving joint analysis of multiple follow-up data points, we will use generalized linear mixed models (GLMMs), which allows direct modeling of the correlations between data points (e.g. repeated measurements on individuals), and the inclusion of individuals with incomplete data (e.g. missing time points). The GLM(M) framework also allows simple controlling for confounders (if necessary) through their inclusion in the model as covariates. For the analysis of binary outcomes, we will use logistic GLM(M)s and for numeric outcomes (CPaSS-D) we will initially specify linear (Gaussian) GLM(M)s; this choice will be scrutinized by analyzing model diagnostics. The primary inferential target will be the coefficient for the treatment group indicator, making this effectively equivalent to ANCOVA. Adjustment for the baseline measurement is important for guarding against regression to the mean in RCT studies, which will be our approach when analyzing only a single follow-up (e.g. 12 months). However, when analyzing multiple follow-ups within the same model, using this baseline adjustment across the board (i.e. at every time point) can result in overestimation of the treatment effect; baseline adjustment for only the first follow-up corrects this overestimation while also guarding against regression to the mean, which will be our approach. We will also conduct exploratory tests of the interaction between time and treatment group, to see whether there is strong evidence that the treatment effect diminishes over time, which will inform future studies.

SubAim: We will determine moderators (caregiver sex, race, child age) of the intervention effects and mediators (Self-Determination Theory constructs of autonomy, relatedness, competence; emotional state at ED recruitment). We hypothesize that disparities in CRS use can be reduced through a tailored intervention that increases autonomous motivation, competence, and relatedness for correct CRS use. Statistical Analysis: Moderation: Within the GLM(M) modeling framework described above, we will evaluate moderation by including interactions between treatment group and sex-, race-, and age-based groupings. We will test whether sex as a biologic variable modifies the intervention effect, but will be underpowered to detect differences because children are infrequently with a male caregiver when seeking emergency care (10-15% in our pilot data). Mediation: We will use Imai's counterfactual mediation method, a very general framework that takes models for the outcome and mediator as inputs, to estimate mediation effects. Traditional mediation analysis can be susceptible to bias due to model misspecification and design limitations and limitations in the classes of statistical models, that can be accommodated (typically linear regressions for both the mediator and outcome) but the Imai framework can accommodate a wide variety of distributions for both the mediator and the outcome; our initial specification will be a logistic model for the binary variables and a linear (Gaussian) model for the numeric variables. We will use a quasi-Bayesian approach to draw from conditional distribution of the mediator given the "treatment" and other covariates, and that of the outcome given the exposure, mediator, and other covariates, to construct hypothesis tests for the proportion mediated; this approach is robustly implemented in the R package mediation.

Aim 2: To use latent growth curve models to identify characteristic trajectories for the CPaSS observed in digital photographs over the 12 study months and assess for correlates of the trajectory types. We hypothesize that caregivers will have different CPaSS trajectories in response to the basic intervention, the enhanced intervention, and the control condition. We expect to identify at least four patterns of response. Statistical Analysis: We will determine 12-month CPaSS trajectory types using adaptations of finite mixture models for the case where the units are trajectories. We will define trajectory shapes through methods combining semiparametric regression modeling (e.g., generalized additive models), with latent class regression, using the R package flexmix. Following convention, we will use the Bayesian Information Criteria (BIC) to determine the appropriate number of latent classes, while also making practical considerations to avoid overfitting, such as not allowing overly small groups to be created, as in previous applied work. Using the posterior probability of class membership, we place individuals into trajectory groups and treat that grouping as the outcome in a subsequent multinomial regression model to determine correlates of class membership, including treatment group, caregiver race/ethnicity, and child age group. If substantial uncertainty in class membership is found, outcomes in the multinomial model will be weighted accordingly.

7 Safety and Adverse Events

Dr. Macy will be responsible for reporting Adverse Events and Other Reportable Information and Occurrences to the Lurie Children's Hospital IRB and the Project Officer for this grant as required and as applicable to the study protocol. The timing of reporting of an adverse event by Dr. Macy will be dependent on the severity of the event and whether the adverse event was expected (included in the informed consent).

7.1.1 Data and Safety Monitoring Plan

The Principal Investigator, Dr. Macy will be responsible for the data safety and quality in this low risk trial of a behavioral intervention to promote correct and consistent size-appropriate child passenger safety behaviors. It should be noted that all research project involving human participants at Ann & Robert H. Lurie Children's Hospital of Chicago, including the proposed

one, require approvals from the Lurie Children's Hospital Institutional Review Board. In addition, because of the potential for sensitive data collection using digital photographs, a Certificate of Confidentiality will be obtained for this study from The Eunice Kennedy Shriver National Institute of Child Health and Human Development.

Dr. Macy will ensure that all relevant IRB policies, procedures and stipulations are followed. She will be responsible for ensuring that other investigators and project staff adhere to IRB policies including (1) All participants will understand, agree to and sign a written consent forms before participating; (2) strict adherence to a participant's right to withdraw or refuse to answer questions will be maintained; (3) the questionnaires will be confidential and no names will be associated with the survey data; (4) consent forms and identifying information will be kept separate from the participants' study data; (5) all identifying information in digital form (e.g., follow-up tracking data) will be encrypted and saved on password-protected devices and stored in a locked filing cabinet. All identifying information on paper (e.g., consent documents) will be stored in a locked filing cabinet. Computers using de-identified data for analysis will be password protected; and (6) participants will be informed in the consent form with information to contact the PI, the project manager, and the IRB office with any questions and/or concerns. Dr. Macy will be responsible for submitting any amendments to the IRB before making changes to the study protocol.

Dr. Macy will be responsible for providing training to all research staff conducting assessments regarding procedures for managing anticipated issues. Specifically, this training will include information regarding warning signs of distress that occur as a result of the surveys and the means for addressing such issues and minimizing distress. Staff will receive training from Dr. Macy in management procedures if a caregiver reveals child abuse/neglect or concerns about safety. Staff training will include review of the study protocol regarding the limits of confidentiality, liaison with ED staff (i.e., clinical care providers) regarding concerns, and circumstances in which it may be necessary to report suspected child abuse or neglect to the Illinois Department of Children and Family Services (DCFS). Study procedures will also require staff to immediately contact Dr. Macy in cases where these concerns arise. Dr. Resnicow will be responsible for training in Motivational Interviewing (MI) techniques. The project manager will facilitate monitoring for fidelity to the intervention throughout the course of the study by reviewing and scoring audio recordings from MI sessions. A consultant with mastery of MI techniques and teaching of MI will provide feedback and additional training as needed.

The digital photographs submitted by caregivers will be reviewed by a trained research assistant within 24-48 hours of the submission. The research assistant will score the photographs with the Child Passenger Safety Score and determine which feedback messages will be returned to the caregivers in the intervention group. For the development of the CPaSS Tool, in collaboration with Dr. Reed and child passenger safety technicians, Dr. Macy has developed a list of child passenger safety behaviors that constitute serious misuse (for example, having a car seat sitting on the vehicle seat but not attached in any way). To offer greater protection in a crash to all children involved in the study whose caregivers have not obtained a size-appropriate child restraint system at the 6- or 12-month follow-up assessment, we have budgeted to provide size-appropriate restraints at no cost to the participants at the conclusion of the study. To receive a CRS from the study, we will require caregivers to have the restraint installed with the guidance of a certified child passenger safety technician. This will ensure the restraint is installed in accordance with manufacturer specified guidelines.

Dr. Macy will be responsible for reporting Adverse Events and Other Reportable Information and Occurrences to the Lurie Children's Hospital IRB and the Project Officer for this grant as required. The timing of reporting of an adverse event by Dr. Macy will be dependent on the severity of the event and whether the adverse event was expected (included in the informed consent).

7.1.1.1 Data Safety Monitoring Board

Dr. Macy will work with 2 certified child passenger safety technicians to form a data safety monitoring board. If serious misuse is identified, caregivers will receive pre-determined corrective text messages regardless of the treatment condition. If the research assistant has any questions about the potential for a serious misuse, the photograph will be brought to the attention of the data safety monitoring board and reviewed for determination of serious misuse. Communication to caregivers about serious misuse will occur within 2 days of receipt of the photographs by the study team. Illegal behavior depicted in photographs will be protected within the legal rights provided by the Certificate of Confidentiality.

8 Study Administration, Data Handling and Record Keeping

8.1 Confidentiality

Information about study subjects will be kept confidential and managed according to the requirements of the Health Insurance Portability and Accountability Act of 1996 (HIPAA). Those regulations require a signed subject authorization informing the subject of the following:

- What protected health information (PHI) will be collected from subjects in this study
- Who will have access to that information and why
- Who will use or disclose that information
- The rights of a research subject to revoke their authorization for use of their PHI.

All research staff assisting with data collection, entry and analysis will receive training in the appropriate data handling and confidentiality procedures. Staff will be made aware that a breach of confidentiality is reason for dismissal.

8.2 Data Collection and Management

Data will be collected only after written informed consent has been obtained from the participant.

Six primary sources of data exist for this study:

1) Screening process completed by the research coordinator
The screening process will use information that can identify the child of the caregiver participating. The identifiable information that will be collected and stored in the screening/recruitment log and enrollment log is the child's name, medical record number, date of birth, and date of being screened for study. The investigators need to collect this information because it will help us to avoid approaching families who have already participated in the study or decided not to participate. These tracking logs will be stored on an encrypted hospital device and will not be shared with persons not on the study team unless a Lurie HIPAA release form has been signed.

2) Surveys (screen, baseline, 6- and 12-month follow-ups) completed by the participant
Caregivers will be approached for screening and baseline survey discretely in the ED waiting room or privately in their child's ED treatment room. We will use a private area, separate from other patients in the ED, for review of the part 1 informational handout and the part 2 informed consent document. Caregivers will be informed of the time commitment and activity on their part necessary for the completion of the research project. Data collection in the ED will be completed privately and confidentially on a study iPad and can be self-administered unless the caregiver prefers to have the research assistant administer the survey verbally. If the survey is administered verbally, the necessary measures will be taken to ensure privacy of response options of the caregiver. Follow-up assessments similarly will be self-administered on study iPads during the 6- or 12-month follow-up visit, unless the participant chooses to complete the follow-up survey prior to the follow -up visit. Then the participant will complete the survey at their convenience and on their own device (e.g., phone, laptop, iPad, etc.).

Children of caregivers enrolled in the study will have information collected about their weight and height at ED recruitment and in-person follow-up assessments. The children will be receiving emergency medical care in the LCH ED. All research staff who will have interactions with children will receive training from Dr. Macy, a board-certified pediatrician and pediatric emergency physician, related to their interactions with children for the measurement of weight and height as well as the direct observation of the child in the vehicle which they travelled to the ED and at follow-up assessments.

Subject identifiers (i.e., subject ID numbers) will be present on all surveys to link their data to their identifying information. The Qualtrics Research Suite (http://www.qualtrics.com/) will be used to collect and store data from the screening survey and part of the 6 month follow-up survey (The Newest Vital Sign survey). The project-specific Qualtrics site is protected with a researcher-designated log-in name and password. The site houses the surveys and compiles data that staff will then transfer to a secured database on the LCH secure network. There are systems in place that prevent the survey from being taken by the same user more than once. No identifying information is collected from participants; so information remains anonymous and in aggregate form. Additionally, Qualtrics has SAS 70 Certification and meets the rigorous privacy standards imposed on health care records by the Health Insurance Portability and Accountability Act (HIPAA). All Qualtrics accounts are hidden behind passwords and all data is protected with real-time data replication. Qualtrics complies with the U.S. and E.U. Safe Harbor Framework and the U.S. and Swiss Safe Harbor Framework as set forth by the U.S. Department of Commerce regarding the collection, use and retention of personal information from European Union member countries and Switzerland. Qualtrics has certified that it adheres to the Safe Harbor Privacy Principles of notice, choice, onward transfer, security, data integrity, access, and enforcement.

Data obtained in the baseline and follow-up surveys will be stored on a password protected, secure server at the University of Michigan. The University of Michigan Center for Health Communications Research (CHCR) has been subcontracted for this R01 to program the surveys, the study website, and the tailoring of web content using the Michigan Tailoring System (MTS) open-source software. MTS does not have any sort of central server that communicates back and forth with a intervention web "site" – so there is minimal security risk in adding it to any project. MTS functions where it takes code generated by CHCR and then uses a library to interpret how to correctly display that code on the intervention site. Baseline, follow-up and intervention data will be coded according to participant ID number. Additionally, a formal review of the survey system and study web "site" is being completed by the University of Michigan Information Assurance Office (https://safecomputing.umich.edu/about). Once completed, the

information assurance assessment will be shared with the Information Management and Institutional Review Board teams at Lurie Children's.

All data collected through the University of Michigan and stored on University of Michigan password protected secure servers will be accessible to the study team via a dashboard. Data will be downloaded to password protected secure servers at Lurie Children's for analysis. We will develop databases for the storage of de-identified data. Files will be stored and backed-up using password protected secure file servers at LCH. Multiple imputation may be employed to address missing data due to dropout or inability to complete follow-up (anticipated <20%).

We will minimize the burden of the research assessments through the use of skip patterns. In order to minimize pressure to provide the perceived "correct" answer, caregivers will be reminded there are no "right" or "wrong" answers to any of the assessment questions. Caregivers will also be informed that they have the right to skip any question that makes them uncomfortable or that they do not wish to answer. They will be informed of their right to withdraw from the study at any time without penalty. Study staff will be trained to respond to emotional distress and to discuss concerns and issues with the project manager, Dr. Macy, and in her absence, Dr. Carter or Dr. Resnicow.

We will also have a Certificate of Confidentiality (CoC) from The Eunice Kennedy Shriver National Institute of Child Health and Human Development to protect study participants' data regarding illegal behaviors captured within any of the survey from subpoena and to conserve their own right to request release of their data for their own purposes.

Data collection from all surveys will occur electronically according to the information presented above, unless there are technical problems during the study. To account for technical issues, paper copies of surveys will be used to complete any data collection needed with participants. Data collected via paper will be transferred to the secure online systems as soon as possible by a trained research team member and the paper documents will be shredded afterwards.

Subject identifiers (i.e., subject ID numbers) will be present on all surveys. The subject identifiers will be used to link information to each participant as opposed to using unique identifying information. When patients enroll in the part 2, baseline phase of the study, names will be linked to individual ID numbers in this secure online database created by CHCR, which will be kept in a restricted access folder on a secure server at the University of Michigan. Data exported from the University of Michigan servers for future data analysis will be de-identified before saving to a password-protected folder on secure network at LCH.

3) Monthly photo submissions and text messages from caregivers of their child as they normally ride in their vehicle

Caregivers will submit photos of their child as they normally ride in their vehicle once a month to the study team. Text messages will be sent and photographs submitted to the study team through a third party platform called Twilio (Twilio Inc., San Francisco, CA) (see www.twilio.com for information about Twilio and its privacy/security policies). Twilio will transmit messages and store links to the MMS (photograph submission) and SMS (text message) content on Twilio servers. Twilio will save the image files to an AWS Server. The Twilio link to the MMS message files will be deleted with an API command sent each day at midnight Eastern Time. MMS files are deleted from the AWS Server after 30 days but will not be searchable after the MMS file link is deleted at midnight. SMS file records will be deleted with an API command sent at the conclusion of the caregiver's participation in the study (i.e., 1 month after completion of the 12-

month follow-up). This will ensure we have a complete record of communication sent to them by the study team.

The study team will make every effort to keep data safe and secure. The University of Michigan Center for Health Communications Research has been subcontracted for work on this project. A formal review of the study website is being completed by the University of Michigan Information Assurance Office (https://safecomputing.umich.edu/about). Data submitted to Twilio will be transmitted by Twilio to the University of Michigan and saved at the University of Michigan in password-protected folders on a secure network. Only study team members will have access to data.

Data collected for the study through the University of Michigan systems will be downloaded to Lurie Children's and stored in a password protected folder on a password-protected, secure network. All photos will be de-identified – meaning faces will be blurred – when saved to Lurie Children's network. Photos will be deleted from the University of Michigan secure server once they are successfully saved to Lurie Children's network.

We will also have a Certificate of Confidentiality (CoC) from The Eunice Kennedy Shriver National Institute of Child Health and Human Development to protect study participants' data regarding illegal behaviors captured within the digital photographs from subpoena and to conserve their own right to request release of their data for their own purposes. The CoC will protect all digital photographs collected during the study.

4) Child Passenger Safety Score (CPaSS-D) and (CPaSS-R) used by the research assistant for direct and remote observation of the Tiny Cargo check-ins (car seat inspection)

The CPaSS electronic survey tool will be used by research assistants to observe and record assessment scores for the Tiny Cargo check-ins each month that a participant is enrolled in the study. The CPaSS tool will be used either in direct observation with a family or via photos submitted electronically via text message to the study team.

CPaSS observations recorded from review of the digital photographs will be collected in surveys on the Northwestern University portal to the Qualtrics survey platform that contain study-specific codes only. Qualtrics will house the data for the CPaSS assessments and will only use a subject identifier (ID number) to connect the participant's data with their information. No unique identifying information will be collected within this assessment tool. When it is exported from Qualtrics, the data will be saved to a secure folder on the LCH secure network.

5) Audio recordings of the MI counseling sessions (conducted at the baseline visit, and/or Month 7/8 via phone)
Although the focus of this study is not on child abuse, because of the nature of the study, caregivers may disclose information that raises concerns for potential child abuse or neglect during the MI counseling sessions (baseline visit and/or phone session). The consent form will contain a statement regarding the mandatory reporting requirements. Staff training will include review of the study protocol regarding the limits of confidentiality, liaison with ED staff (i.e., clinical care providers) regarding concerns for potential child abuse or neglect, and circumstances in which it may be necessary to report suspected child abuse or neglect to Illinois Department of Children and Family Services (DCFS). Study procedures will also require staff to immediately contact Dr. Macy in cases where these concerns arise.

MI counseling sessions for this study will be conducted/facilitated by the RA with participants and will be audio recorded (with signed permission from participants) in order to ensure the fidelity of the intervention. Staff will be trained to administer the TCBD MI counseling intervention in a specific way and in order to ensure all staff are conducting and coding the data correctly, it is necessary to audio record this portion of the study. Audio recordings of MI counseling sessions will not include the caregiver's name or any information that could be used to identify them. Audio records will be coded only with the research assistant ID, subject ID and study name, and date of the session. Taping will be done using a LCH secure device (study iPad and/or study iPhone), and counseling sessions will be immediately uploaded to a password protected LCH server folder and deleted from the device. All audio recordings will be destroyed from the LCH server as soon as fidelity monitoring is completed.

6) Paper study documents --consent forms, proof of payment and study follow-up information
All paper study documents (e.g., consent forms, payment forms, follow-up related contact forms, etc.) with patient identifying information will be kept stored in locked file cabinets separate from any data collection forms. Each file cabinet will have a unique key and will be kept within a locked office, within a locked suite and within a locked building accessible by LCH badge only.

Subject identifiers (i.e., subject ID numbers) will be present on study-related forms. The subject identifiers will be used to link information to each participant. When patients enroll in the part 2, baseline phase of the study, names will be linked to individual ID numbers in this database, which will be kept in a restricted access folder on a secure server.

Signed consent forms will be kept in a locked file cabinet in a locked research office at the study site. All information collected as part of both research projects will be accessible only to research staff who have completed mandatory training in the protection of human subjects.

Follow-up contact information that the caregiver provides to the study team will be stored separately with a unique study identifier to link information. The dataset with identifiers will be destroyed as soon as possible after the completion of 12-month follow-up. Caregiver identifiers (contact information) will be retained for the purposes of follow-up.

The study team will follow the federal guidelines as the necessary time that all study materials will kept following the conclusion of the study recruitment and analysis period.

<u>Blinding for purposes of data collection:</u>
This is a unique single-blinded study where a portion of the study will include blinding of study team members. The blinding will not include the Principal Investigator or the Study Coordinator. Also, participants will know which group they are enrolled in as the consent form states the study groups as well as the extent of study involvement based on which group you are enrolled in.

The portion of the study that includes blinding are the monthly photo submissions that participants will send us via text message over the course of 12 months.
Study team members that directly enroll participants in the ED will not be able to review any monthly photo submissions from those families. They will only be able to review and score (using the CPaSS tool) photo submissions of families that they did not directly enroll, so they are blinded to which study arm the family is enrolled in.

Study team members that are only assisting with the review and scoring (using the CPaSS tool) of photo submissions each month will be blinded to the study arm the participant is enrolled

within. Because they will not be enrolling participants within the ED and they will not have access to the study dashboard where the photos are submitted, these study team members will remain blinded throughout the course of the study and will only provide scoring based on what they observe within the photos. These study team members will relay the scoring of all photo submissions to the rest of the team to record within the database and the additional study team members will send out text message feedback to the families based on any major misuse/errors that are visible within the photo submissions.

8.3 Records Retention

Study investigators will follow the federal guidelines as to how long all data collection and records should be retained past study conclusion.

9 Study Monitoring, Auditing, and Inspecting

9.1 Risks

Potential Risks:

Physical: None anticipated

Psychological: There is no more than minimal risk that caregivers may experience stress associated with seeking ED care for their child and may find the intervention inconvenient. Participants may become anxious, upset, or uncomfortable as a result of being asked personal questions. There is a theoretical risk that participants may feel pressure to provide the “correct” answer. Participants will be reassured that there are no right or wrong answers to the study questionnaires, that their answers are confidential, and that the data will be made anonymous by removing all potential identifiers. Children may become anxious with the measurement of their weight and height by a research staff member who is unfamiliar to them. Children will not be separated from their caregivers at any time during the study.

Social: Because the research requires the collection of personally identifying data, there is always a risk of breach of confidentiality. This risk is no greater than that which occurs in every medical encounter. We will make every attempt to minimize that risk. Only the principal investigator, Dr. Macy, project manager, and research assistants will have access to personally identifying contact information for the enrolled caregivers, which will be destroyed at the earliest possible time. The photographs collected in the course of this study will be stored separate from the other data (survey questions and measures) in a secure LCH server accessible to the principal investigator, Dr. Macy, and the research assistants responsible for coding the photographs.

Breach of Data: Accidental public release of the survey data or text messages (SMS or MMS) may occur due to unplanned data breaches including hacking or other activities outside of the procedures approved by this study. If a security break happens, their data may be misused or used for unapproved reasons.

Financial: None anticipated

Legal: Caregivers will be made aware that spontaneous disclosure of behaviors concerning for child abuse or neglect (not directly assessed in the study) will be reported to the Illinois Department of Children and Family Services (DCFS). Caregivers also may fear legal repercussions for reporting their child passenger restraint practices if they do not adhere to the state law. We have determined, in consultation with experts from the Child Abuse Team at Lurie

Children's Hospital that, while illegal, it is not considered child abuse or neglect in the State of Illinois to use a restraint other than a legally required restraint, unless the child were injured as a result of the improper restraint.

Identification of safety hazards: There is always the potential for human error and for the research coordinator to miss identifying a major misuse or error in the way the participant's child is using their child restraint system.

Adequacy of Protection against Risks:

Recruitment and Informed Consent

All study procedures, protocols, measures, and informed consent documents will be reviewed and approved by the Lurie Children's Hospital Institutional Review Boards before any activities involving human subjects begin. Waiver of informed consent will be requested for review of the ED Electronic Medical Record (EMR)/Patient Tracking board to determine which families to approach for screening. Adult caregivers of children 6 months to 10 years old, who bring their child to the LCH ED for any non-critical illness or injury, will be approached for eligibility screening. Again, the child age range was selected based on the American Academy of Pediatrics Guidelines for Child Passenger Safety.

Caregivers will provide verbal consent for the ED screening. Eligible caregivers will complete separate written informed consent for 1) participation in the intervention study with baseline/follow-up assessments and 2) the collection of data using digital photographs. Prior to beginning any data collection with a participant, a trained research assistant will present and explain a comprehensive informed consent document, reviewing the study risks and participants' rights. After all questions are answered to the satisfaction of the participant, and after he/she signs the consent document, then data collection will begin. Participants will be given a copy of the consent form(s).

Protections against Risk:

Data collection will be conducted in accordance with Institutional Review Board guidelines for human subjects' protection and patient confidentiality. All research staff assisting with data collection, entry and analysis will receive training in the appropriate data handling and confidentiality procedures. Staff will be made aware that a breach of confidentiality is reason for dismissal.

Physical: None anticipated

Psychological: We will minimize the burden of the research assessments through the use of skip patterns. In order to minimize pressure to provide the perceived "correct" answer, caregivers will be reminded there are no "right" or "wrong" answers to any of the assessment questions, that their answers are confidential, and that the data will be made anonymous by removing all potential identifiers, only a study ID number will be used. Caregivers will also be informed that they have the right to skip any question that makes them uncomfortable or that they do not wish to answer. They will be informed of their right to withdraw from the study at any time without penalty. Study staff will be trained to respond to emotional distress and to discuss concerns and issues with the project manager, Dr. Macy, and in her absence, Dr. Carter or Dr. Resnicow.

Children may become anxious with the measurement of their height by a research staff member who is unfamiliar to them. Children will not be separated from their caregivers at any time during

the study. The child's height will be measured laying down or standing up, depending on what is most comfortable for the child.

Also, caregivers will be approached for screening discretely in the ED waiting room or privately in their child's ED treatment room. We will use a private area, separate from other patients in the ED, for review of the consent document. Caregivers will be informed of the time commitment and activity on their part necessary for the completion of the research project. Data collection in the ED will be completed on a study iPad and can be self-administered unless the caregiver prefers to have the research assistant administer the survey verbally. Follow-up assessments similarly will be administered on study iPads or can be completed remotely via the participant's own personal device and privately, at their convenience.

Social: We will make every attempt to minimize the risk of breach of confidentiality. Only the principal investigator, Dr. Macy, project manager, and research assistants will have access to personally identifying contact information for the enrolled caregivers, which will be destroyed at the earliest possible time. The photographs collected in the course of this study will be stored separate from the other data (survey questions and measures) in a secure LCH server accessible to the principal investigator, Dr. Macy, and the research assistants responsible for coding the photographs.

Also, subject identifiers will not be present on any data collection forms. Follow-up contact information will be stored separately with a unique study identifier to link information. The dataset with identifiers will be destroyed as soon as possible after the completion of 12-month follow-up. Caregiver identifiers (contact information) will be retained for the purposes of follow-up. These identifying data and the digital photographs (with the child's face obscured) will be kept in a secure database on a LCH server accessible from password-protected study computers and archived in a password-protected file on the principal investigators office desktop computer on the LCH network. A separate dataset for analysis will be created, which will contain only unique study identifiers. Audio recordings of MI sessions will be destroyed as soon as fidelity monitoring is completed.

Breach of Data: We have a Certificate of Confidentiality (CoC) from The Eunice Kennedy Shriver National Institute of Child Health and Human Development to protect study participants' data regarding illegal behaviors captured on the digital photographs from subpoena and to conserve their own right to request release of their data for their own purposes. The CoC will protect all digital photographs collected during the study.

Although the focus of this study is not on child abuse, because of the nature of the study, caregivers may disclose information that raises concerns for potential child abuse or neglect. The consent form will contain a statement regarding the mandatory reporting requirements. Staff training will include review of the study protocol regarding the limits of confidentiality, liaison with ED staff (i.e., clinical care providers) regarding concerns for potential child abuse or neglect, and circumstances in which it may be necessary to report suspected child abuse or neglect to Illinois Department of Children and Family Services (DCFS). Study procedures will also require staff to immediately contact Dr. Macy in cases where these concerns.

The study team will make every effort to keep data safe and secure. The University of Michigan Center for Health Communications Research has been subcontracted for work on this project. A formal review of the survey system and study website is being completed by the University of Michigan Information Assurance Office (https://safecomputing.umich.edu/about). Text messages will be sent and photographs submitted to the study team through a third party

platform called Twilio (Twilio Inc., San Francisco, CA) (see www.twilio.com for information about Twilio and its privacy/security policies). Twilio will transmit messages and store links to the MMS (photograph submission) and SMS (text message) content on Twilio servers. Twilio will save the image files to an AWS Server. The Twilio link to the MMS message files will be deleted with an API command sent each day at midnight Eastern Time. MMS files are deleted from the AWS Server after 30 days but will not be searchable after the MMS file link is deleted at midnight. SMS file records will be deleted with an API command sent at the conclusion of the caregiver's participation in the study (i.e., 1 month after completion of the 12-month follow-up). This will ensure we have a complete record of communication sent to them by the study team.

Data submitted to Twilio will be transmitted by Twilio to the University of Michigan and saved at the University of Michigan in password-protected folders on a secure network. Only study team members will have access to data.

Data collected for the study through the University of Michigan systems will be downloaded to Lurie Children's and stored on a password-protected, secure network. All photos will be de-identified – meaning faces will be blurred – when saved to Lurie Children's network. Photos will be deleted from the University of Michigan secure server once they are successfully saved to Lurie Children's network.

All paper study documents (e.g., consent forms, locator forms) will be stored within locked study file cabinets within a locked office and within a locked suite at Lurie Children's Hospital. All identifiable info will be stored within separate locked file cabinets as well.

Financial: None anticipated.

Legal: We have determined, in consultation with experts from the Child Abuse Team at Lurie Children's Hospital that, while illegal, it is not considered child abuse or neglect in the State of Illinois to use a restraint other than a legally required restraint, unless the child were injured as a result of the improper restraint. Our study team will not be approaching the caregivers of any children seeking care in the ED for a motor vehicle related injury.

Identification of safety hazards: The study team will make every effort to identify misuse and errors in the way that participants are using their child restraint system. In order to ensure the study team is educated on the identification of safety hazards, several training sessions have been conducted with the study team and led by certified child passenger safety technicians at LCH. The Study Coordinator and PI of this study have been certified as child passenger safety technicians as well. They will both oversee study procedures and will consistently review the feedback provided to participants during the Tiny Cargo check-ins (both in-person and via photos submitted by the parents). We will plan to conduct booster training sessions with the study team throughout the course of the study to serve as fidelity checks and keep the study team up to date on the child passenger safety information. We will also plan to refer participants directly to the Lurie Children's Buckle Up Program (car seat program) if any participants have questions or concerns that the study team does not feel they can answer or if the participants' questions fall outside of the training provided for this study. All participants enrolled in this study will also receive a handout with some online resources and phone numbers for places they can get their car seat checked for proper installation by a certified child passenger safety technician.

<u>Third party to whom data will be released:</u>

Data collected as part of the surveys, website, and photo submissions will be released in real-time to the Center for Health Communications Research (CHCR) at the University of Michigan. CHCR is a National Cancer Institute Center of Excellence in Cancer Communications Research since 2003, along with its former embodiments, the Health Media Research Laboratory (University of Michigan) and the Health Communications Research Laboratory (University of North Carolina at Chapel Hill), has conducted research in health communications since 1992. The CHCR comprises an interdisciplinary team of behavioral scientists, health educators, instructional designers, media professionals, computer engineers, graphic designers, project managers and students from a wide variety of disciplines (public health, psychology, medicine, biostatistics, computer engineering, information science, art and others). Principal CHCR staff have worked together for over a decade and have established a shared knowledge base and language regarding tailoring technology and intervention development. Each team member is familiar with the theories and intricacies involved in developing heath behavior interventions and conducting research trials. The interactions among the CHCR's professionals from various disciplines foster the generation of innovative ideas, creative solutions to obstacles and unique perspectives.

CHCR has a significant history of developing and implementing interactive individually tailored interventions as a team, developing and examining the effectiveness of tailored print-telephonic-, computer-, mobile- and web-based interventions. These interventions have used an array of media channels and technologies, including web, computer, print, kiosk, cell phone/mobile device, IVR (interactive voice response), video/DVD and social media applications. This research has been carried out in a variety of settings, including health clinics, emergency departments, primary care offices, schools, homes, inner-city community centers, shopping malls, libraries, work sites and senior centers. Target populations have included patients, physicians, health maintenance organization members, adolescents, the elderly, minorities, military personnel and the under-served. Topics have included smoking cessation and prevention, dietary change, physical activity, screening, genetic counseling, risk prevention, treatment decision-making, medication adherence and survivorship.

The CHCR-developed Michigan Tailoring System (http://chcr.umich.edu/mts), is a standards-based, cross-platform, open source application released to the research community in 2008 that enables the efficient creation, testing and delivery of richly tailored communications without the need for constant technical support and assistance from a team of computer programmers. These communications may include individually tailored text and media (photos, graphics, animations, audio, video) and be delivered via a range of channels, including print, computer, web, e-mail, cell phone and social media applications. Because of the scalable, common technical platform, resulting tailored programs are easier to disseminate.

9.2 Benefits

<u>Direct Benefits:</u>

Depending on which group a participant is randomly assigned to, the caregiver may or may not get any direct benefit from taking part in this study. Receipt of the motivational interviewing session and child passenger safety information is likely to increase the caregiver's motivation and competence for the consistent use of the size-appropriate child passenger restraint, which may result in better child occupant protection in the setting of a motor vehicle collision.

Other direct benefits to the participants may include:

- Access to the study's website ("Car Seat Compass") with info on car seat safety.

- A new car seat, if certain conditions are met (e.g., car seat is recalled, expired, or damaged/missing parts, participant's only reported reason for not using a child restraint system is insufficient finances)
- Feedback from a study team member about safer ways to travel with their child. They may get this feedback via text message or verbally from a staff member.
- A private counseling session with a Car Seat Coach (in the ER or via phone). They would discuss how they feel about car seats and how their child usually rides while in the car.

Indirect Benefits:
The proposed research will potentially benefit others by leading to an effective intervention for the promotion of size-appropriate child passenger safety behaviors.

Injury remains the leading cause of death among children after the first year of life. Motor vehicle occupant-related injuries are the top-ranking cause of unintentional injury deaths among children older than 4 years. Car seats, booster seats, and seat belts are proven effective for reducing the risk of injury and death in a motor vehicle collision. However, appropriate child passenger restraint use remains below targets such as those stated in the Health People 2020 Objectives. Finding ways to ensure that every child is using the size-appropriate child passenger restraint on every trip is one of the most critical injury prevention needs today, yet few effective evidence-based precision prevention approaches exist to reach the caregivers who practice suboptimal child passenger safety behaviors. By identifying at-risk families during a child's ED visit, the proposed intervention could remarkably enhance the universal injury prevention efforts already in place in communities and physician offices. As such, the knowledge gained from this study is of vital importance to public health efforts for childhood injury prevention.

9.3 Risk Benefit Assessment

There is no more than minimal risk that caregivers may experience stress associated with seeking ED care for their child and may find the intervention inconvenient. Participants may become anxious, upset, or uncomfortable as a result of being asked personal questions. There is a theoretical risk that participants may feel pressure to provide the "correct" answer. Participants will be reassured that there are no right or wrong answers to the study questionnaires, that their answers are confidential, and that the data will be made anonymous by removing all potential identifiers. The risk of a potential data breach is offset by the potential for new knowledge about promoting child passenger safety best practices. Children may become anxious with the measurement of their weight and height by a research staff member who is unfamiliar to them. Children will not be separated from their caregivers at any time in the course of the study for study related purposes. Children may need to be separated from their caregivers as part of their clinical care in the ER.

9.4 Informed Consent Process / HIPAA Authorization

All caregivers will provide verbal consent for the ED screening. Verbal consent will be obtained using an informational sheet template for waiver of obtaining a signed consent form from Lurie Children's IRB. This sheet provides sufficient information for subjects to make an informed decision about their participation in this study. Caregivers who screen eligible for study enrollment after completing the screening survey will then complete a separate informed consent discussion with trained study staff and this process will include written documentation of consent to the 1) participation in the randomized control trial of the intervention with baseline/follow-up assessments and 2) collection of data using digital photographs.

Prior to beginning any data collection with a participant, a trained research assistant will present and explain a comprehensive informed consent document, reviewing the study risks and participants' rights. After all questions are answered to the satisfaction of the participant, and after he/she signs the consent document, then data collection will begin. Participants will be given a copy of the consent form(s).

All consent related documents (informational sheet and consent form) will be submitted with the protocol for review and approval by the IRB for the study. Subjects will be consented by the study project manager or research assistants in the ED waiting room or the ED treatment rooms. Potential subjects will review the consent form in detail with the person designated to consent (either PI or CRC) and have the ability to take the consent home for further review.

9.4.1 Alterations to Typical Consent Process

9.4.1.1 Waiver of Consent (In some cases for screening/portions of that study that qualify as minimal risk, a waiver of documentation of consent may be permissible IRB SOP)

<u>Waiver or alteration of required elements of consent: According to HHS CFR 45.46.116(d)</u>: An IRB may approve a consent procedure which does not include, or which alters, some or all of the elements of informed consent, or waive the requirements to obtain informed consent. In order to qualify for a waiver or alteration to the informed consent process, please justify each of the following:

- the research involves no more than minimal risk to the subjects;
- the waiver or alteration will not adversely affect the rights and welfare of the subjects;
- the research could not practicably be carried out without the waiver or alteration; and
- whenever appropriate, the subjects will be provided with additional pertinent information after participation.

Waiver of informed consent will be requested for review of the ED Electronic Medical Record (EMR)/ER Patient Tracking board to determine which families to approach for screening. Adult caregivers of children 6 months to 10 years of age, who bring their child to the LCH ED for any non-critical illness or injury, will be approached for eligibility screening. Again, the child age range was selected based on the American Academy of Pediatrics Guidelines for Child Passenger Safety.

10 Study Finances

10.1 Funding Source

This study is financed through a grant from the National Institute of Health (NIH), National Institute of Child Health and Human Development (NICHD).

10.2 Conflict of Interest

All study investigators will follow Lurie Children's Hospital of Chicago's Policy on Conflicts of Interest Related to Research.

10.3 Subject Stipends or Payments

<u>Study-related costs:</u>

The study team is paying for the following:

- Cost of text messages (both SMS and MMS) sent to a participant's phone from the study team while taking part in the study (12 months).
- Cost of new car seats, if provided to families.

The participant will be responsible for the following:

- Depending on their data plan, cost of text messages (both SMS and MMS) sent from their phone to the study team while taking part in the study (12 months).
- The cost of traveling to and from the 6 and 12-month follow-up visits (e.g., gas, public transportation).

The participant and/or their insurance company are responsible for the costs of normal care at LCH. Lurie Children's may be able to provide some financial help to patients. Ask their health team for more information about this program.

<u>Study payments:</u>

The participant will be paid up to $155 in total for taking part in all components of this study. They will only be paid for completed study visits. See the table below for information on the amounts they can earn for each of the tasks that they can finish while in the study.

Study Timeline	Task	Time to do task	Amount	Rate of payment	Type of payment	When payment is given	How payment is given
**Emergency room visit: $25**							
ER visit (enroll in study)	Baseline survey	Within 48 hours	$20	1 time	gift card	ER visit	In-person or via mail
	Tiny Cargo check-in	Within 48 hours	$5	1 time	gift card	ER visit	In-person only or via mail
**Monthly Photo Requests: $50**							
Photo request (8 over 12 months)	Monthly photo request	Within 4 hours	$5/each month	Up to 8 times	gift card	6 or 12 month follow-up visit	In-person or via mail
	4 months of photo requests	4 months in a row	$5/every 4 months	Up to 2 times	gift card	6 or 12 month follow-up visit	In-person or via mail
**6 month follow-up visit: $35**							
6 month follow-up visit	Confirm 6 month visit	Prior to 6 month visit	$5	1 time	gift card	6 month follow-up visit	In-person or via mail
	Survey & Tiny Cargo check-in	Within 1 month	$30 ($15 survey/ $10-15 TC check-in)	1 time	gift card	6 month follow-up visit	In-person or via mail
**12 month follow-up visit: $45**							
12 month follow up visit	Confirm 12 month visit	Prior to 12 month visit	$5	1 time	gift card	12 month follow-up visit	In-person or via mail
	Survey & Tiny Cargo check-in	Within 1 month	$40 ($25 survey/ $10-15 TC	1 time	gift card	12 month follow-up visit	In-person or via mail

Study Timeline	Task	Time to do task	Amount	Rate of payment	Type of payment	When payment is given	How payment is given
			check-in)				

11 Publication Plan

Dr. Macy will ensure the intervention trial is registered at ClinicalTrials.gov, that the information entered into
ClincialTrials.gov is accurate, and that results from the trial will be submitted to ClincialTrials.gov at the completion of the study. The study will be registered at ClinicalTrials.gov prior to the recruitment of subjects, anticipated in October/November 2019. The results of the trial, including participant flow, demographic and baseline characteristics, primary and secondary outcomes, statistical tests, and adverse event information, will be uploaded within 1 year of the collection of 12-month outcomes from the final participant. Informed consent documents will reflect the plan to post the study information and results at ClinicalTrials.gov. Dr. Macy will adhere to Lurie Children’s Hospital policies for clinical trials registration and results reporting.

Project Title: Tiny Cargo, Big Deal! An Adaptive ED-Based eHealth Intervention to Promote Correct and Consistent Size-Appropriate Child Passenger Safety Behaviors and Reduce Disparities

Principal Investigator: Michelle Macy, MD, MS, Attending Physician, Division of Emergency Medicine, Ann & Robert H. Lurie Children’s Hospital of Chicago

Name of Federal Funding Agency: National Institutes of Health, National Institute of Child Health and Human Development (NICHD)

Modifications to study protocol

- Modification 1 (12/10/2019): Change to tango e-gift card system for study incentives

- Modification 2 (12/30/2019): Addition of Spanish families within study population and translation of all study materials
- Modification 3 (4/15/2020): remote recruitment, study personnel change, new recruitment materials, edits to screening survey
- Modification 4 (6/23/2020): study personnel change, change to remote recruitment methods
- Modification 5 (11/24/2020): 4 new remote recruitment methods, exclusion/inclusion criteria, edits to screening survey
- **Modification 6 (3/26/21):** adding new sites for part 1 screening of study (Lurie affiliate EDs & Lurie Immediate Care Centers), increased total N for part 1 screening surveys to 10,000

Background and Study Rationale

This document serves as the study's research protocol and the described study will be conducted in compliance with the provisions set forth in the protocol as well as, Good Clinical Practice standards, associated federal regulations, and all applicable Lurie research requirements.

This study will be conducted in full accordance with all applicable Lurie Children's Hospital IRB Policies and Procedures and all applicable Federal and state laws and regulations.

1 Introduction

Motor vehicle collisions (MVCs) remain the leading cause of unintentional injury deaths among children in the United States (U.S.) and racial/ethnic minority children are disproportionately impacted as suboptimal child passenger safety behaviors are more prevalent in some communities. Existing universal approaches to promote child passenger safety have fallen short of ensuring that all child passengers are correctly using size-appropriate child passenger restraints according to guidelines published by the American Academy of Pediatrics and the National Highway Traffic Safety Administration. Precision prevention programs are urgently needed to improve child passenger safety behaviors among caregivers who have not been responsive to guidelines, laws, and public education campaigns. The proposed research will test the efficacy of Tiny Cargo, Big Deal (TCBD), an emergency department (ED)-based precision prevention intervention grounded in Self-Determination Theory. TCBD integrates personalized counseling based on principles of motivational interviewing (MI) and eHealth components including a tailored educational mobile friendly website "site" and short message service (SMS) communications with the goal of improving child passenger safety. We hypothesize that by providing tailored child passenger safety education and personalized skills for restraint use in a manner that supports autonomous motivation the TCBD intervention will be more efficacious than universal approaches (laws/guidelines) for realizing correct use of size-appropriate child passenger restraints.

1.1 Background and Relevant Literature

The overarching scientific premise of the proposed study is derived from the published literature demonstrating that the emergency department (ED) can be a site of screening to identify suboptimal child passenger safety behaviors[1-3] and can serve as a site of intervention,[4-10] that tailored child passenger safety information can increase parental knowledge,[6,11] and that eHealth holds promise for child passenger safety education.[6,12-16] Additionally, our own pilot work in the ED suggests that child passenger safety behaviors can be improved and disparities reduced through a *brief counseling session* and *tailored educational brochures*. Despite this

collective evidence, suboptimal child passenger safety behaviors are prevalent and a key contributor to injuries sustained in motor vehicle collisions (MVCs).

Motor vehicle collisions (MVCs) remain a significant and costly public health problem in the United States (U.S.). MVCs are *the #1 cause* of unintentional injury-related deaths among 4-12 year olds and a leading cause of unintentional injury-related deaths among younger children.[17,18] **In fact, more U.S. children die from MVCs than die of heart disease, chronic lower respiratory diseases, influenza/pneumonia, or sepsis each year.**[18,19] Child passenger fatality rates have been stagnant around 650 per year from 2010 and 2014.[18,20] In 2015, crash fatality rates in the U.S. rose by the largest percent in 50 years.[21,22] Non-fatal injuries also represent an enormous burden. In 2013, more than 131,000 children received emergency treatment for non-fatal injuries sustained in MVCs,[23] and more than half of these children required hospital admission or transfer to another ED.[23] Childhood survivors of MCVs can experience lifelong sequelae, including mobility impairments, irreversible brain or spinal cord injuries, and post-traumatic stress disorder.[24-26]

Universal strategies[27] to promote child passenger safety provide recommendations for the population as a whole and are disseminated in national guidelines, state laws, and public education campaigns.[28-35] These strategies have reached many caregivers but **current recommendations are not adopted for all child passengers.**[36-38] Precision prevention strategies,[27] targeted to those who have not responded to universal strategies, are urgently needed to reduce unintentional injuries from MVCs.[16,39] Suboptimal child restraint system (CRS) use is lowest for infants, with about 10% prematurely using a forward-facing car seat with a harness or booster seat. Suboptimal CRS use increases considerably after a child's first birthday; roughly 30% of toddlers use a booster seat or seat belt alone and >50% of elementary school age children use a seat belt alone when they would benefit from a more protective restraint.[36-38,40] The greatest risk for injury is among children who travel unrestrained. This is permitted by caregivers in increasing proportions as children progress from toddlers (5%) to teens (15%).[38] Proportions of fatally injured unrestrained children increase from 27% among <4 year olds to 45% for 8-12 year olds.[41]

Prior child passenger safety research has generally encouraged use of car seats or booster seats for children in traditional age categories.[6,9,13,14,42-44] We propose to move beyond one-size-fits-all age-based approaches with an innovative precision prevention intervention that: 1) *targets caregivers* with suboptimal child passenger safety behavhiors; 2) integrates child size, anticipated growth trajectory, and current CRS into *personalized recommendation*s for *correct use* of a *size-appropriate restraint*; 3) *tailors messages* on caregiver race/ethnicity and Self-Determination Theory constructs (autonomy, relatedness, competence); and 4) has a second level of *increased intensity* to address caregivers who do not change their child passenger safety behavior by Month 6 of the study.

This proposal aligns with the NICHD Pediatric Trauma Research Program goal to reduce morbidity and mortality in childhood through injury prevention. The national strategy on highway safety has become focused on the goal of moving "Toward Zero Deaths" on U.S. roadways.[45] In addition, the CDC has declared **child passenger safety is a "Winnable Battle"** based on the large potential impact and known effective strategies to address transportation-related injuries.[46] Healthy People 2020 Objectives for Unintentional Injury Prevention include increasing age-appropriate child restraint system (CRS) use in children with age-group specific targets that are far from optimal due to limited adoption of current recommendations.[47]

The fact that seat belt use reduces the risk of injury is indisputable.[48] However, **vehicle seats and seat belts are designed for adults not children.**[49-51] Children experience dramatic physical and biomechanical changes before reaching the stature (around a height of 57") and maturity necessary to achieve proper fit with an adult seat belt in a vehicle seat.[52-60] To accommodate the range of child growth and development, distinct CRS modes (rear-facing car seats, forward-facing car seats, and booster seats), have been designed to provide greater levels of protection than an adult seat belt alone can provide to a child in a crash.[61] CRS, used correctly, prevent injury and save lives. Rear-facing CRS use until age 2 lowers the odds of serious injury in a frontal crash 5-fold compared with forward facing.[62] Forward-facing car seats reduce risk of serious injury for toddlers by 70% and risk of death by 25% compared with seat belts.[48] Booster seats cut the risk of non-fatal injury among 4 to 7-year-old children in half compared with seat belts alone.[63,64] This evidence provides the foundation for the 2011 updates to child passenger safety recommendations from the American Academy of Pediatrics (AAP),[32,61] which state a child should: 1) remain rear-facing as long as possible, until at least age 2 or reaching the weight or height limits of their rear-facing CRS; 2) then use a forward-facing harness restraint until reaching the weight or height limits of that CRS; 3) then use a booster until proper seat belt fit can be achieved usually between ages 8 and 12; and 4) always ride in a back seat.

Addressing suboptimal child passenger safety in minority populations is crucial to eliminate disparities. Death rates among passengers <13 years are significantly higher for black compared with white children (0.73 vs. 0.57 per 100,000) and fatally injured black children are almost twice as likely to have been unrestrained compared with white children (45% vs. 26%).[41] In national observational data, we found suboptimal child passenger safety behaviors, including premature transitions to a less protective restraint and travelling unrestrained, were more common among minority children.[65] In survey data we obtained from caregivers in two Michigan EDs, racial differences in child passenger safety behaviors persisted after adjusting for caregiver education, income, and sources of child passenger safety information.[1]

Research shows that ED visits are a window of opportunity for behavior change. We have selected the ED as the location to initiate our intervention because ED visits are frequent among U.S. children, totaling more than 25 million annually.[66,67] In 2014, about 20% of children visited a hospital ED at least once.[68] Children who utilize the ED may have limited access to primary care services and many come from poor and minority populations.[69-71] As a result, some families in the ED have less exposure to primary care-based injury prevention programs. Public health challenges, including child passenger safety,[4,9] adult seat belt use,[5,72] smoking cessation,[73-75] substance abuse,[76-78] and violence prevention[79-81] have been addressed in the ED. Conducting our study in a community hospital ED will complement the literature from child passenger safety studies conducted in large children's hospital EDs[6,9,11] and be more generalizable to the types of EDs that provide emergency care to most U.S. children.[82]

2 Study Objectives

The proposed randomized, adaptive trial will test the efficacy of a selective intervention, *Tiny Cargo, Big Deal* (TCBD), to improve child passenger safety among caregivers who are not adherent to current national recommendations. The TCBD intervention, grounded in Self-Determination Theory (SDT) and developed as a part of Dr. Macy's K23 award, consisted of a brief motivational interviewing (MI) session in the ED and a mailed brochure tailored on child age, size, and restraint use. TCBD proved feasible and acceptable with *significant promise for improving child passenger safety behaviors* at 6-month follow-up. We have enriched TCBD based on pilot participant feedback to include a streamlined ED counseling session, new eHealth components, and one booster MI session delivered by phone. To replace the paper

brochures, we have designed a prototype mobile friendly web "site" that has shown positive results in usability testing. The study site will be optimized for mobile devices and provide to the intervention group with continuous access to *personalized, precise* child passenger safety information. We will augment the intervention with tailored short message service (SMS) communications (i.e., text messages) that facilitate bi-directional flow of information after the ED visit.

We hypothesize that a precision prevention intervention (TCBD) that provides tailored child passenger safety education and personalized skills for restraint use in a manner that supports autonomous motivation will be more efficacious than universal approaches (laws/national guidelines) for realizing correct use of a size-appropriate child passenger restraint. The primary outcomes, size-appropriate restraint use and the Child Passenger Safety Score (CPaSS), will be assessed with direct observation at scheduled, in-person appointments and remote observation of digital photographs submitted by caregivers in response to unscheduled text messages.

We will test our hypothesis using a randomized adaptive trial design with the following aims: **Aim 1:** To test the efficacy of the TCBD intervention compared to usual care for improving primary outcomes [i.e., size-appropriate restraint (dichotomous) and CPaSS-D (numeric)] at 6 and 12 months. **Aim 2:** To use latent growth curve models to identify characteristic trajectories for the CPaSS-R observed in digital photographs over the 12 study months and assess for correlates of the trajectory types. This innovative, theoretically grounded research will have long-standing impact on child passenger safety by informing the best methods to increase correct use of size-appropriate child restraint systems among caregivers of diverse backgrounds at the point they interact with the health care system. The work has the potential for wide-scale implementation in the ED and for translation to primary care offices, schools, and community settings with an ultimate goal of eliminating child passenger safety disparities and reducing the number of childhood injuries from MVCs.

2.1 Primary Objectives

The primary objectives of this efficacy trial are to:
1) test the efficacy of the TCBD intervention compared to usual care for improving primary outcomes [i.e., size-appropriate restraint (dichotomous) and the Child Passenger Safety Score (CPaSS) (numeric)] assessed at 12 months
2) use latent growth curve models to identify characteristic trajectories for the CPaSS observed in digital photographs submitted to the study team by caregivers over the 12 study months. Correlates of the trajectory types will be assessed

2.2 Secondary Objectives

Additionally, we will determine moderators (caregiver sex, race, child age) and mediators (Self-Determination Theory constructs of autonomy, relatedness, competence; emotional state at ED recruitment) of the intervention effects at 12-month follow-up.

Finally, we plan a cost analysis of the intervention to inform wider dissemination and implementation in settings outside of the ED. We will measure the total costs of the intervention from participant and health system perspectives by gathering estimates of intervention resource requirements, including personnel costs, implementation time, participant time, and material costs. We will calculate costs of the intervention per child. Net costs (savings) will be calculated by subtracting mean costs per participant in the control condition from mean costs per participant who receive the basic TCBD and the enhanced TCBD interventions. We will also

estimate cost-effectiveness as measured by dollars per caregiver using a size-appropriate CRS and using the CRS correctly.

3 Investigational Plan

Study Phase: Remote Recruitment
Study Group: All participants
Setting: Remote
Timing: Recruitment will occur within 6 months of ED visit, potential participants will complete baseline study components within 5 business days of screening eligible for study and agreeing to participate.

For patients that visited the LCH ED, eligibility for screening will be determined from reports generated from Epic. The invitation to complete the eligibility screening questionnaire will be distributed by email, text message, phone call, postcard, flyer in ED room, ED discharge summary paperwork, and MyChart Research Recruitment messages.

For patients that have not visited the LCH ED and are seeking care at LCH affiliated sites, we will not use EPIC. The only recruitment method used for the part 1 screening survey will be flyers hung in the patient treatment rooms at these sites. Patients will have the opportunity to scan the QR code or enter the survey link onto their phones or personal device to take the survey remotely. Once a family completes the screening survey, the RC will determine eligibility for part 2. All part 2 procedures will occur remotely between the parent and the RC.

For initial recruitment of study participants for the part 1 screening survey, we will be using 5 methods of remote recruitment techniques. The following techniques will be used: :

1) POSTCARDS: The use of postcards mailed to the parents of children eligible for this study and who have not responded to our previous efforts to reach them via email, text message and/or phone. This would be a final attempt to reach them via a mailed postcard.

2) FLYER IN ED ROOMS AT LCH: The use of a one page flyer to be hung up within all patient emergency rooms at LCH as another method to recruit families utilizing the ED in real time. The flyer will have a QR code and screening survey link on it.

3) FLYERS IN PATIENT TREATMENT ROOMS AT LCH AFFILIATED SITES: The use of a one-page flyer to be hung up within patient treatment rooms at Lurie Children's affiliate EDs (e.g., Central DuPage, Northwest Community) and Lurie Children's Immediate Care Centers (Lincoln Park and Northbrook). They flyer will have a QR code and screening survey link on it.

4) ED DISCHARGE SUMMARY INVITATION: The use of adding an invitation to complete our research study within the ED discharge summary notes that every patient/family will get when they leave LCH. Approval has been obtained from the Department of Emergency Medicine to add a few sentences along with a QR code and survey link to the bottom of the ED discharge summary notes for all patients at LCH.

5) MyChart RESEARCH RECRUITMENT: The use of the platform MyChart Research Recruitment as another method of reaching out to the parents of children eligible for our research study and inviting them to participate via messages sent to them in the portal.

Potential participants who screen eligible from the screening survey, will complete the informed consent process for part 2. The consent documents will be emailed to participants and available

via a link to a Qualtrics survey in which the participant can provide their electronic signature. Informed consent discussion will occur between the potential participant and the research coordinator by phone prior to distribution of the baseline survey. Study procedures will otherwise follow the flow outlined below.

Study Phase: Baseline Visit (study enrollment)
Study Group: All participants
Setting: Emergency Department at LCH or remote (via phone/text message/email)
Timing: Study procedures must be complete during ED visit or within 5 business days

- Data collected from medical chart in EPIC: Information that will be recorded from the medical record includes: MRN number, chief complaint, weight, name of child, date of birth, and age. We will also use the medical chart to make sure the child does not meet any of the exclusion criteria as specified in the protocol and study staff are able to approach the family. And Epic reports will be used to identify families who have utilized Lurie Children's ED in the 6-months prior to the current date for remote recruitment.
    - If the family did not visit the LCH ED, then the RC will not use EPIC to record any information. The parent enrolled will provide the study with the necessary information in the screening survey.
- Screening survey: If a child's parent/legal guardian chooses to be screened for eligibility in the full study, they will complete a short survey (~10 min.) on a study iPad or remotely on their own personal device. The survey will ask about their safety behaviors for kids in and around cars. It will ask about how their child usually rides in cars too. They should answer all questions for the child that is getting care at Lurie Children's. All answers are private, meaning they will not be shared with anyone outside of the study team.
- Baseline survey: If the child's parent/legal guarding screens eligible for the full study and consents to participate, they will complete a (baseline) survey on an iPad or remotely on their own device. This will take about 15-20 minutes. The survey will have questions about their use of car seats, and thoughts on child safety in and around vehicles. If they cannot finish the survey in person, they will have 5 business days to do this survey over the phone with a staff member or on their own device.
- After they get done with the survey, they will be randomly assigned (like flipping a coin) to one of two groups. They cannot pick the group that they will be in. The groups are:
    - Group 1 (Control --Usual Care): A study team member will give them an informational handout ---**see section 9 of IRB app "supporting document" for the PDF called, "Study Handout"**
    - Group 2 (TCBD Intervention): A study team member will give them an informational handout --**see section 9 of IRB app "supporting document" for the PDF called, "Study Handout".** They will also have about a 20-minute counseling session (in-person or over the phone) with a study team member. During this session, they will talk about how their child normally rides in their car, their goals for traveling safely with their child, and how they might reach these goals. To protect their privacy, we will do this session in a private place. We will give the parent/legal guardian the option to have other family members/friends with them in the ED be present during the counseling session or step out of the room during the session. Also, to make sure that we've recorded their answers correctly, we ask for their consent to audio tape this session. This recording will only be used for research and the audio tapes will not include info that someone could use to recognize them. They can give consent to audio tape within the informed consent.

We will also show participants randomized to the intervention group how to use the study's website called, the "Car Seat Compass" (about 10 minutes). This website will give them custom

feedback based on their answers within the baseline survey. This website will give them answers to common questions about traveling with children.

Two test cases have been built out on the development website, so that the IRB is able to log in and review content that may be similar to two common occurrences we expect to see within the study. To view these test cases, please follow the instructions listed below:

Test case 1 (study ID 400): Parent (Mother) who has a 12 month old child, currently using a rear-facing car seat, but plans to prematurely transition their child to a forward-facing car seat within the next 6 months. We are recommending that the child stay rear-facing until at least 2 years of age.

6. Using google chrome, go to website: http://macytcbd.miserver.it.umich.edu.
7. Log in with the email: bpollock@luriechildrens.org and the password: TCBDstudy
8. Review content within website based on this parent's situation.
9. The home page illustrates a road map of child restraints and provides the family with a green light message since the family is currently using the recommended car seat (rear-facing car seat) for that child. However, because they are planning to transition their child to a forward-facing car seat in the next 6 months, they will be eligible for the study and receive additional information on this in the "just for me" tab of website.
10. The tab labeled "just for me" surfaces information based on that parent's specific responses to the baseline survey and goes into more detail about the recommended car seat for the child of the caregiver.

Test case 2 (Study ID 300): Parent (Father) who has a 6 year old child, currently using a seat belt to restrain child in vehicle. We are recommending that they child is restrained with a booster seat.

6. Using google chrome, go to website: http://macytcbd.miserver.it.umich.edu.
7. Log in with the email: bethpollock822@gmail.com and the password: TCBDstudy
8. Review content within website based on this parent's situation
9. The home page illustrates a road map of child restraints and for this family, provides a caution (yellow light message) since the family is using a non-recommended child restraint for the child. You will see the booster seat is highlighted in green to illustrate the recommended restraint for this family.
10. The tab labeled "just for me" surfaces information based on that parent's specific responses to the baseline survey and goes into more detail about the recommended car seat for the child of the caregiver.

During the baseline visit or within 5 business days, both groups (control and intervention) will also:

- Fill out a form that'll be used to get in touch with them for future follow-up visits --**see section 9 of IRB app "supporting document" for a document called, "Study Locator Form"**
- Complete a Tiny Cargo check-in. During this check-in, a study staff member will view how their child normally rides within their car. If they cannot do this check-in in-person, they will have 5 business days to send us photos. These photos will show us a few different views of their child as they normally ride in their car.

- ****See section 9 of IRB app, "supporting documents" for a document called, "TCBD text library and error messages" and refer to the sheet labeled, "Response to errors."** This document provides scripted responses that the RA will use when major misuse and/or errors are visible during the Tiny Cargo check-ins. The TCBD intervention messages are referred to as "Scripted MI response" while the control group's responses are referred to as "scripted control response."

Study Phase: Monthly Photo Requests (During Months 1-5 and Months 7-11)
Study Group: All participants
Setting: Remote (text message via caregiver's mobile device)
Timing: Study procedures must be complete within each month (within 4 weeks)

<u>Monthly photo requests</u>**:**

- ****See section 9 of IRB app, "supporting documents" for a document called, "Tips & tricks for taking your monthly photo"** --this document will be provided to all participants enrolled in part 2 of the study. The RA will review this document during the ED baseline visit with the participant.
- No matter which group caregivers are assigned into at the baseline visit, we will contact the study participant again starting in 1 month. A member of our study team will send them a photo request via text message. This text message will ask them to share photos of their child as they normally ride in their car.
- These photo requests will be sent to their best phone number. All photos of their child will be sent back to us by the parent/legal guardian.
- They will get requests to submit photos a total of 8 times while taking part in this study (over the next 12 months). We will be asked for these photos during months 1- 5, and Months 7-11 of the study.
- They will get these photo requests only on the days and during the times that they tell us they are able to get messages from us. To update the days and times that they are free to be contacted, caregivers can reach out to our team at by phone or email or text message
- The participant will have a 4 hour window of time to submit their photo requests each month with an additional 4 hour grace period. If a participant submits their photos during their window of time each month and the photos meet the necessary requirements (meaning the RA is able to visibly see all elements of the child sitting in the car seat, then the photos will be approved and the participant will earn $5 that month. Participants can earn a total of $40 if they complete all 8 photo requests during their specified window each month and the photos are approved. If the participant completes 4 months of photo submissions in a row, then they will hit a "streak" and will earn an additional $5. They can hit a "streak" up to 2 times over the course of the study, so they can earn up to an additional $10.
- Photo message response time (evaluate and response) from study staff will be 24-48 hours. Photo requests will not be sent if the staff are not available to respond during that time window.
- For study data collection purposes, after the research team receives photos back from the study participant, the research team will use the Child Passenger Safety Score (CPaSS) to access for correct use of the child's restraint system. See below for a document called "CPaSS data elements for IRB submission" to view the tool that will be used to access for visible errors/misuse that may be seen by the study team.
- If major misuse or errors are visible within a participant's photo submissions, they will receive a scripted text message from the study team within 24-48 hours after the photo is submitted. Participants in the TCBD intervention group will receive messages in the spirit of Motivational Interviewing style while the control group will receive similar messages without the spirit of MI.

- ****See section 9 of IRB app, "supporting documents" for a document called, "TCBD text library and error messages" and refer to the sheet labeled, "Response to errors."** This document provides scripted text messages that the RA will use when major misuse and/or errors are visible within the photo submissions each month. The TCBD intervention messages are referred to as "MI spirit text messages" while the control group's text messages are referred to as "control text messages."

Study Phase: Study Text Messages (During Months 1-12)
Study Group: All participants
Setting: Remote (text messages via caregiver's mobile device)
Timing: Study procedures will occur each month

Study text messages:
No matter which group caregivers are randomly assigned to, all study subjects will receive monthly text messages from our team while they are taking part in the study (12 months).

- Group 1 (control): They will get about 3-4 text messages each month. Messages will include:
  - Reminders for upcoming follow-up visits
  - Feedback related to the photos they send us of their child riding in their car
  - General study info

- Group 2 (TCBD intervention): They will get about 5-6 custom text messages. Messages will include:
  - Reminders for upcoming follow-up visits
  - Feedback related to the photos they send us of their child riding in their car
  - Tailored text messages --Custom tips and info based on their answers to study surveys
- ****See section 9 of IRB app, "supporting documents" for a document called, "TCBD library and error messages" and refer to the sheet labeled, "general message library."** This document provides a library of custom tips/general supportive and informational text messages that the TCBD intervention group may receive over the course of the study (12 months). Participants will receive these text messages as applicable based on their answers within the surveys, based on the time of year as well as priority of message content.

Study Phase: Follow-ups --Month 6 and Month 12
Study Group: All participants
Setting: In-person or remote (via photo submission/video chat, phone)
Timing: Within 1 month

6- and 12-month follow-up visits:

- All subjects will be asked to complete a 6- and 12-month (follow-up) visit.
- The follow-up visits include:
  - Taking an online follow-up survey (about 20-25 minutes) --the 6 month follow-up survey will also include a health literacy assessment called the Newest Vital Sign. These questions will not be asked during the 12 month survey.
  - Completing an in-person Tiny Cargo check-in (about 10 minutes)
  - At the 6 month follow-up ONLY: Receiving a study handout on the car seat recommendations for children as put out by the National Highway Traffic Safety Administration (NHTSA) **--see section 9 of IRB app for a copy of the handout called, "NHTSA child passenger safety overview"**

- Survey: The follow-up survey can be done in person on an iPad, on the study participant's own device, or over the phone with a study team member. A team member will send them a link to take the survey so that they can do it before the visit. The follow-up survey will have questions that are like those asked on the baseline survey.
- Tiny Cargo Check-in: During the check-in, a study team member will look to see how their child usually rides while in their car. Every effort will be made to meet with the study participant and their child in person for the 6- and 12-month follow-up visit. This meeting can take place at Lurie Children's or at another location that is closer to them. If they are unable to meet with us in person (e.g., they move out of state), they may also send us photos of their child as they usually ride in their car via text message or via a video chat (e.g., Facetime or google hangout).
- ****<u>See section 9 of IRB app, "supporting documents" for a document called, "TCBD text library and error messages" and refer to the sheet labeled, "Response to errors."</u> T**his document provides scripted responses that the RA will use when major misuse and/or errors are visible during the Tiny Cargo check-ins. The TCBD intervention messages are referred to as "Scripted MI response" while the control group's responses are referred to as "scripted control response.
- After the 6-month follow-up assessment, caregivers in the intervention group only who continue suboptimal CRS use will be re-randomized to receive either the basic or enhanced intervention.
- The <u>enhanced TCBD intervention group</u> will include one additional motivational interviewing (MI) session by phone in Month 7/8 and one to two additional tailored text messages in Months 7-12 (total of 7 tailored tips during these months compared to 4 tailored tips for those in the basic TCBD). The extra text messages will be sent to those in the enhanced TCBD group until size-appropriate restraint use is observed. Caregivers receiving enhanced TCBD will be encouraged to obtain a free seat check with a certified child passenger safety technician (CPST) in the community.
- The <u>basic TCBD intervention group</u> will continue on in study without any changes.

Study Phase: Phone Counseling Session --Month 7/8
Study Group: Enhanced TCBD Intervention Group
Setting: In-person or remote
Timing: Within 1 month

<u>Phone counseling session (enhanced TCBD intervention)</u>:

After the study subject completes their 6-month follow-up visit, the study team will determine if the child is using the recommended child restraint system (car seat or booster seat) for their age and size. The intervention group subjects whose children are NOT using the recommended restraint will be randomly assigned (like flipping a coin) to one of two groups. They cannot pick the group they will be in.

The groups are:

- Group 2 (basic TCBD): They will keep taking part in the study with the basic intervention and nothing will change.
- Group 3 (enhanced TCBD): They will keep taking part in the intervention arm of the study but will be asked to talk with a Car Seat Coach (i.e., a behavioral research assistant trained for the study) by phone for about 20 minutes. The phone call will cover some of the challenges and successes they are having with traveling safely with their child. They will also get 1 or 2 additional study text messages each month. These extra text messages will include:
    - Feedback related to the photos they send us of their child riding in their car
    - Feedback related to survey answers

3.1 General Design

The Tiny Cargo, Big Deal! (TCBD) emergency department (ED)-based intervention is designed to promote correct and consistent use of a size-appropriate child passenger restraint system on every trip. We will test the precision prevention intervention with a randomized, adaptive trial design. Caregivers (parents and legal guardians) of children who seek care for non-critical illness and injury at the Ann & Robert H. Lurie Children's Hospital of Chicago Emergency Department will be screened. With this most recent IR modification (3/26/21), we will also add recruitment from Lurie Children's affiliate EDs (e.g., Central DuPage, Northwest Community) and Lurie Children's Immediate Care Centers (Lincoln Park and Northbrook). Eligible caregivers will have a child 6-months to 10-years-old and <55 inches tall who screen positive for suboptimal child passenger safety behavior or plans for premature transition to a less protective restraint in the coming year. Caregivers who agree to participate will be randomized to the basic TCBD intervention (an ED counseling session plus eHealth after ED discharge) or the control condition (usual ED care with discharge instructions relevant to the child's diagnosis and a car seat information sheet). At 6-months we will re-randomize caregivers in the intervention group alone who are not using a size-appropriate CRS to either continue basic TCBD or receive the enhanced TCBD, which includes one motivational interviewing (MI) session by phone in Month 7/8 and four instead of two text messages in Months 7-12.

3.2 Allocation to Interventional Group

After completing the baseline survey, all caregivers will be randomized into one of two groups: 1) TCBD intervention group, or 2) control group. Computerized, block randomization (block sizes from four to eight) will be applied to assign eligible caregivers to the TCBD intervention or control group in a 3:1 ratio. Blocks will be stratified by age groups related to child passenger safety recommendations (<2 years – rear-facing CRS, 2-4 years – forward-facing CRS, 5-10 years – booster seat) and the child passenger safety behavior that led to eligibility for study (not using the recommended restraint currently or planning a premature transition in the coming 6 months).

After the 6-month follow-up assessment, caregivers in the intervention group only who continue suboptimal CRS use will be re-randomized by computer in a 1:1 ratio to receive either the basic or enhanced intervention. The enhanced TCBD will include one additional motivational interviewing (MI) session by phone in Month 7/8 and one to two additional tailored text messages in Months 7-12 (total of 7 tailored tips during these months compared to 4 tailored tips for those in the basic TCBD). The extra text messages will be sent to those in the enhanced TCBD group until size-appropriate restraint use is observed. Caregivers receiving enhanced TCBD will be encouraged to obtain a free seat check with a certified child passenger safety technician (CPST) in the community. The primary outcomes (size-appropriate CRS use and CPaSS) will be assessed in-person at Months 6 and 12. The secondary outcome, the CPaSS trajectory, is a measure of correct and consistent restraint use in response to the intervention and will be assessed by scoring photographs submitted by caregivers (Months 1-5; 7-11).

STUDY GROUPS:

After participants complete the baseline survey, they will be randomized to either the control group (usual ED care with discharge instructions relevant to the child's diagnosis and a study handout) or the TCBD intervention group (an ED counseling session plus eHealth after ED discharge). Please also refer to section 3. D.1. for a complete outline of the study details for each group over the course of the study.

Main differences between intervention and control include the following:

At baseline visit (enrollment in study):

1) Control group -- after taking the baseline survey, they will be given a study handout that summarizes the IL law on child passenger safety and provides a few online resources for parents on child passenger safety.
2) TCBD Intervention group --after taking the baseline survey, they will receive a brief motivational interviewing inspired counseling session with the research coordinator in addition to the same study handout sheet that the control group will receive. During the MI counseling session, participants will talk to the RC about how their child normally rides in their car, their goals for traveling safely with their child, and how they might reach these goals. Additionally, the intervention group will receive direct access to the study's mobile friendly website "site," Car Seat Compass. The site will provide parents with custom feedback based on their answers within the baseline survey regarding how their child normally rides in their vehicle and their general child passenger safety practices with that child. The site will give them answers to common questions about traveling with children as well. The control group will not have access to this site throughout the study. However, the control group will obtain access to the study website after the conclusion of their 12 month follow-up.

Both groups will participant in all of the Tiny Cargo check-ins and the 6- and 12 month follow up visits scheduled over the course of the study. Those elements of the study do not change based on the group someone is enrolled in.

Monthly Photo Requests:
Both groups will be asked to send the study team photos of how their child normally rides in their vehicle and will be paid the same incentive amounts. However, if major misuse or errors are visible within the photos that participants submit, then the text messages sent to participants will vary slightly depending on which group you are enrolled in.

1) control group --they will receive a concise text message for any major misuse/errors that are visible and how to adjust for it. Non-critical errors will not elicit a direct text message response from the study team.
2) TCBD intervention group --they will receive a slightly longer text message that is framed within the spirit of motivational interviewing that addresses the major misuse/error and possible solutions to adjust for it. They will also receive tailored messages for any non-critical misuse/errors.

** See section 9 (Supporting documents) of the IRB application for an excel file that serves as a library of text messages that will be provided to participants when major misuse/errors are visible (in-person or via photos) based on the study group they are enrolled in. This is a sample of the messages that will be used and sent out, but not meant to be a comprehensive library of messages. The RC will use their discretion when reviewing misuse/errors and adjusting messages as needed.

Study Text Messages:
Both groups will receive study text messages from the study team over the course of their enrollment in the study. However, the TCBD intervention group will receive tailored text messages each month on top of the basic reminders for follow-up visits and feedback related to the photos they submit each month.

The tailored text messages that will be sent to the TCBD intervention group will be custom tips and info based on the participants answers to study surveys and the type of child restraint system they are recommended to be using. **Please see section 9 of the application to view a document called, TCBD library and error messages and refer to the sheet labeled, "general message library." The text messages included within this sheet will be used to support the intervention group over the course of the study.

After the 6-month follow-up assessment:

Participants in the intervention group only who continue sub-optimal child restraint system use (not using the recommended seat) will be re-randomized to one of two groups:
2) TCBD basic intervention group
3) TCBD enhanced intervention group

The enhanced TCBD intervention group will include one additional motivational interviewing (MI) session by phone in Month 7/8 and one to two additional tailored text messages in Months 7-12 (total of 7 tailored tips during these months compared to 4 tailored tips for those in the basic TCBD). The extra text messages will be sent to those in the enhanced TCBD group until size-appropriate restraint use is observed. Caregivers receiving enhanced TCBD will be encouraged to obtain a free seat check with a certified child passenger safety technician (CPST) in the community.

The basic TCBD intervention group will continue on in study without any changes.

3.3 Study Measures

Over the course of this study, participants will complete the following surveys: screen, baseline, 6- and 12-month survey. At the 6-month follow-up, the participants will also complete a brief health literacy assessment called the Newest Vital Sign.

The selected measures for the surveys are reliable, valid, and have been pilot tested in the study population. Measures will be assessed at screening, baseline and re-assessed at the 6- and 12-month follow-up visits. Assessments will be self-administered or RC facilitated on a study iPad in the ED and at in-person follow-up assessments (Months 6 and 12). Skip patterns will reduce administration time. Our team has found baseline assessment lasting 20 minutes to be feasible and acceptable to caregivers in the ED setting during pilot testing.

Table 1 illustrates survey measures for this study along with the frequency/timing of assessments.

**Table 1: Measures**	Assessment Month(s)
**Primary Outcome**	
Size Appropriate Restraint Use (dichotomous)	6 and 12
Child Passenger Safety Score direct observation*	6 and 12
**Secondary Outcome**	
Child Passenger Safety Score remote observation	ED through 12
**Exploratory Cost Measures**	
Intervention Time (ED, Phone MI) for RA/Counselor	ED and 2
Intervention Time (ED, Phone MI, texts, web app) for caregiver	ED through 12
Material Costs - iPads, computers, maintenance	ED through 12
Intervention Costs - Photograph review, texts, web app	ED through 12
**MODERATORS/MEDIATORS**	
Caregiver Demographic Characteristics, reason for ED visit	ED
Child Size: Measured weight and length/height	ED, 6, and 12
Self-Determination Theory Constructs	
Treatment Self-Regulation Questionnaire[158-160]	ED, 6, and 12
Confident rulers and Perceived Competence Scale[161]	ED, 6, and 12
Health Care Climate Questionnaire[162]	12 months
Health Literacy and Numeracy	
The Newest Vital Sign[163,164]	6 months
Caregiver Emotional State, Stress, Coping	
State-Trait Anxiety Inventory[165]	ED
Risk Behavior Diagnosis Scale[166]	ED
Parenting stress index	ED
Marlowe-Crowen Social Desirability Scale[168]	6 months
Child Passenger Safety Hassles Scale[169]	ED, 6, and 12
**Footnote added 7/5/2025 for clarity in anticipation of publication of primary outcome: due to COVID-19 pandemic restrictions on in-person study activities, the project was converted to fully remote data collection. We were therefore unable to collect Child Passenger Safety Score data through direct observation.*	

3.4 Study Endpoints

3.4.1 Primary Study Endpoint

The primary outcomes (size-appropriate CRS use and CPaSS) will be assessed in-person at Month 12. The secondary outcome, the CPaSS trajectory, is a measure of correct and consistent restraint use in response to the intervention and will be assessed by scoring photographs submitted by caregivers (Months 1-5; 7-11) and collected by the study team (Months 6 and 12). Photographs will be scored using the CPaSS tool by research team members blinded to the treatment group.

4 Study Population and Duration of Participation

4.1 Duration of Study Participation

Caregivers that agree to enroll in the study will participate for up to 12 months. This includes the screening process, baseline visit, monthly photo requests, and two follow-up visits (6 and 12-months). Study participation by the caregiver/parent will end after the 12-month follow up visit.

4.2 Total Number of Subjects and Sites

To test the Tiny Cargo, Big Deal Intervention, we anticipate the need to screen 10,000 caregivers to recruit 900 caregivers who will be enrolled in the study and randomized to intervention or control. All recruitment will occur in the Emergency Department (ED) at Lurie Children's Hospital of Chicago (LCH) or remotely via phone/email/text message. With this most recent IR modification (3/26/21), we will also add recruitment via flyers in patient treatment

rooms from Lurie Children's affiliate EDs (e.g., Central DuPage, Northwest Community) and Lurie Children's Immediate Care Centers (Lincoln Park and Northbrook).

4.3 Inclusion Criteria

Caregivers (parents or legal guardians) meeting inclusion criteria will be approached to take the screening survey in the ED waiting room or ED treatment rooms during natural breaks in treatment/care or remote recruitment will include email, text message and/or telephone calls to families of children who sought care in the Lurie Children's ED. With this most recent IR modification (3/26/21), we will also add remote recruitment via flyers from Lurie Children's affiliate EDs (e.g., Central DuPage, Northwest Community) and Lurie Children's Immediate Care Centers (Lincoln Park and Northbrook).

For in-person recruitment, research assistants will use the LCH ED electronic tracking board to look for participants with the following inclusion criteria to approach them to take the screening survey and will confirm upon approach that the child and family meet the following inclusion criteria:

- Child (patient) of caregiver is age 6 months to 10 years old at screening
- Child (patient) of caregiver is seeking care in the ED for a noncritical injury or illness or received care in the past 6 months
- Caregiver/parent is the legal guardian of the child seeking care in the ED
- Child of caregiver is less than 55 inches tall

Caregiver speaks English or Spanish (NOTE: Spanish intervention components will be submitted for IRB review after initial IRB approval is granted. The research team will only recruit English-speaking caregivers until IRB approval is granted for the recruitment of Spanish-speaking caregivers)Caregiver is 18 years old or older
Caregiver/parents that complete the screening survey and meet the additional study inclusion criteria will have the opportunity to enroll in part 2 the study. The study inclusion criteria required for enrollment are:

- Caregiver owns a smart phone
- Caregiver rides/drives/travels at least once a week with their child in a car, truck, or van
- Caregiver screens positive for sub-optimal child passenger safety behaviors or who report plans for premature transition to a less protective restraint in the coming 6 months **(see section 9 for additional document, "screen eligibility sheet," that RCs will use to determine eligibility of caregiver for part 2 of the study based on their responses within the screening survey)**
- If enrolled, caregiver is willing to travel to follow-up locations (not applicable as study tasks have gone fully remote)
- If enrolled, caregiver is willing to receive and send text messages each month as part of the study
- Child of caregiver rides unrestrained in the past month
- Child of caregiver rides in the front seat in the past month

The remote recruitment process will include outreach via email, text message, phone, postcard, ED room flyer, and MyChart Research Recruitment to caregivers of children age 6 months to 10 years who were seen in the Lurie Children's ED in the 6 months prior to the current data. Eligibility for contact for screening will be determined based on Epic reports that will include child age, child weight, ZIP code of their home address, and language preference of English or Spanish. Potential participants will be screened to determine if they attest to being

the parent/legal guardian and at least 18 years of age. Caregiver self-report of child's height will be used.

Remote recruitment will also include flyers in patient treatment rooms at LCH affiliate sites – families that may not have visited the LCH ED. Caregivers that are recruited remotely via a the flyers will confirm the above criteria in the screening survey.

4.4 Exclusion Criteria

Caregivers (participants) will not be approached to be screened for this study if they meet any of the exclusion criteria shown below. Special care will be taken to screen out caregivers of deceased children prior to recruitment efforts.

For remote recruitment, research coordinators will use EPIC reports to verify the following criteria and/or will allow the screening survey and conversation with the caregiver to identify for the following exclusion criteria.

For in person recruitment, research coordinators will use LCH ED electronic tracking board and conversation with caregiver to identify for the following exclusion criteria:

- Caregiver does not understand/speak English (*or Spanish once IRB approval granted for recruitment of Spanish-speaking participants*)
- Child of caregiver is seeking care for a critical illness or injury: (ESI=1), treatment being given in ED trauma bay or a negative pressure room, or child requires a sepsis huddle.
- Child of caregiver is seeking treatment for a new long-term diagnosis (e.g., diabetes, cancer) (not applicable while recruiting remotely)
- Child of caregiver has anticipated need for admission
- Child of caregiver is seeking care for child abuse/neglect and/or Child Protective Services is involved in treatment
- Child of caregiver is seeking care that requires intensive psychosocial services (e.g., sexual assault, psychosis/active suicidal/homicidal ideation/attempt)
- Child of caregiver is seeking treatment for a motor vehicle related injury.
- Child of caregiver is too tall for study (height = >54 inches tall)
- Child of caregiver is classified as "break the glass"
- Caregiver has already refused the study twice
- Child of caregiver is already enrolled in another study at LCH (e.g., IMPROVE) (not applicable while recruiting remotely)
- Caregiver completed this study's screen for this child within the past 6 months
- Caregiver is already enrolled in this study for this child or another one of their children
- Caregiver is unable to provide consent (not legal guardian of child seeking care in ED)
- Caregiver is under 18 years of age
- Caregiver lives out of the state orout of the country
- Caregiver is deaf, blind, or visually impaired and unable to provide informed consent

Participants (caregiver/parents) that complete the screening survey and meet the additional study exclusion criteria will also be excluded from enrolling in the study. The study exclusion criteria for enrollment are:

- Child of participant requires a wheelchair or other special needs seating when traveling in vehicles

4.5 Subject Recruitment

Competing studies in ED:
The research study called IMPROVE (IRB #00117338) will be recruiting patients in the ED at LCH and will overlap with our study population in the following patient ages (4-10 years).

If a child comes into the ED at LCH between the ages of 4-10 years of age, and meets criteria for IMPROVE, then our study team will communicate with the Research Coordinators of IMPROVE and allow them to approach the child/family first. If the child becomes enrolled in IMPROVE, then our study team will exclude the child/family from our study and will not approach them. However, if the child is determined to be ineligible for IMPROVE and/or decides not to participate in IMPROVE, then our study team will approach the family to see if they would like to enroll.

If this plan causes any problems with the enrollment numbers for Tiny Cargo, Big Deal, then procedures will be put in place to revise this process as necessary.

** not applicable while recruiting remotely

Recruitment procedures:
Research Assistants will utilize the LCH ED electronic tracking board to continuously monitor incoming patients that meet study inclusion criteria during recruitment shifts. A waiver of informed consent will be requested for review of the ED Electronic Medical Record (EMR)/Patient Tracking board at LCH to determine which families to approach for screening. Adult caregivers of children 6 months to 10 years old, who bring their child to the LCH ED for any non-critical illness or injury, will be approached for eligibility screening.

All participants (caregivers/parents) meeting the basic list of inclusion criteria will be approached by an RA either in the ED waiting room or the child's (patient) ED treatment room to enroll in the part 1 screening survey. Remote recruitment will include email, text message, telephone calls, mailed postcards, ED discharge paperwork, and MyChart Research Recruitment messages to families of children who sought care in the Lurie Children's ED. With this most recent IR modification (3/26/21), we will also add recruitment via flyers from Lurie Children's affiliate EDs (e.g., Central DuPage, Northwest Community) and Lurie Children's Immediate Care Centers (Lincoln Park and Northbrook).

An informational handout sheet will be used by the RA to explain the study and obtain verbal consent to participant before data collection begins. During remote recruitment, subjects will review this informational handout prior to taking the screening survey via a URL link. Research staff will inform the participant of the general nature of the study, their privacy rights, expectations for their participation, the voluntary nature of their participation, and that their participation can be withdrawn at any time during the course of the study. Participants will be assured that refusal to participate will not affect their child's medical care in any way. At all times, research staff will make explicit the voluntary nature of their participation. Confidentiality measures will be explained, including assurances that LCH ED staff and law enforcement will not be informed of involvement in the study and/or responses to survey items (see COC). In addition, limits to confidentiality will be explained (e.g., immediate harm to self/others). Before data collection begins, the RAs will also allow time for the participant to review the informational handout, will ask the participant if they have questions, and will ask for the participant to provide a summary of what was explained to them. Participants will be given a copy of the informational handout.

Consent process:

After obtaining verbal consent, participants will self-administer a computerized screening assessment survey with validated measures assessing for further study eligibility. RAs will stay in the room while the participant takes the survey to be available to assist with patient questions during screening. Study RAs will utilize procedures from prior work to seamlessly integrate the ED-based assessment into the patient’s ED stay to avoid interrupting their care.

For remote recruitment, the participant will record their consent to take the survey at the start of the survey after they have read the informational handout and will voluntarily complete the survey via their own personal device. The will take the survey in the privacy of their own home/location.

After completing the screening survey, the RA will determine if the participant is eligible to enroll in the part 2 baseline phase of the study based on a summary of their responses. Participants that are eligible will be notified of the opportunity to take part in an additional phase of the study and the RA will use a written informed consent document to obtain consent for further study participation. This process will occur in either the ED waiting room (a private space) or within the child's ED treatment room. Or remote consenting of part 2 will occur over the phone where the RA will go over the key points of the consent form. The informed consent includes 1) participation in the intervention study with baseline/follow-up assessments and 2) the collection of data using digital photographs. Prior to beginning any data collection with the participant, a trained research assistant will present and explain the comprehensive informed consent document, reviewing the study risks and participants’ rights. After all questions are answered to the satisfaction of the participant, and after he/she signs the consent document, then data collection will begin. Participants will be given a copy of the consent form(s). The participant will provide signed consent of the parent permission form via Qualtrics for remote consenting.

For remote recruitment, after the subject completes the screening survey, the RC will determine if the participant is eligible to enroll in the study based on a summary of their responses. Participants that are eligible will be notified via phone, email, or text communication and will have the opportunity to enroll in part 2. The RC will follow the exact same procedures as in person recruitment for part 2 except all procedures will occur over the phone.

Waiver of the requirement of obtaining a signed consent form:

We are requesting a waiver of the requirement of obtaining a signed consent form for part 1 of this study only. Part 1 of the study includes a brief (~5-10 minute) screening survey that collects one piece of identifying information (5-digit zip code) and presents no more than minimal risk of harm to participants. This screening survey serves the purpose of determining eligibility for part 2 of this study.

Waiver of informed consent:

We request waiver of informed consent for the retrospective review of visits to the Lurie Children's ED for the purposes of identifying potentially eligible families to screen for eligibility and recruit for participation in the study. The families identified for remote recruitment will be contacted via email, text message and/or phone for initial screening. The remote screening process (Part 1) will include the information sheet distributed via Qualtrics survey or reviewed verbally by the RC over the phone. Any caregiver potentially eligible for Part 2 of the study will complete a phone conversation with a Research Coordinator (RC). Informed consent for participation in part 2 of the study will be conducted verbally over the phone with electronic copies of the consent form delivered to potential participants via email and via a link to a

Qualtrics survey. The Qualtrics survey will allow for the collection of electronic signatures from study participants.

A waiver of informed consent will be requested for review of the LCH ED Electronic Medical Record (EMR)/Patient Tracking board, "EPIC," to determine which families to approach for screening. The ability to view LCH EPIC will be for pre-screening purposes only. The Research Assistants will utilize the LCH ED electronic tracking board to continuously monitor incoming patients that meet study inclusion criteria during recruitment shifts. Adult caregivers of children 6 months to 10 years old, who bring their child to the LCH ED for any non-critical illness or injury, will be approached for eligibility screening.

RA tracking logs will contain information regarding patients' age, gender and chief complaint. This information will help to streamline recruitment so that only eligible patients will be approached and will eliminate approaching non-eligible patients. Minimal clinical data (e.g., sex, race, age, chief complaint) will be collected on ED patients who refuse to participate in the screening survey and on ED patients who the research recruiters fail to approach for the study. Minimal identifiers of potential subjects (first name and birth date) will be kept in order to allow staff time to reconcile recruitment numbers and to avoid re-approaching patients, and this information will be destroyed as soon as possible. The research involves no more than minimal risk and does not adversely affect the rights and welfare of the patients.

This research study requires the use of the child's birth date in order to:
1) include the caregivers/parents of children between the ages of 6 months to 10 years in the study
2) determine if the child is currently using the child restraint system (CRS) that is optimal for their age according to the 2011 child passenger safety recommendations from the American Academy of Pediatrics (AAP). These recommendations provide the foundation for the educational information that is used in this study to provide caregivers/parents on which CRS to use for their child.

Safeguard against potential coercion or appearance of coercion:
The risk of potential coercion will be minimized by following standard procedures for obtaining verbal and/or written informed consent from participants. Study personnel will fully explain the study procedures, risks, benefits, and alternatives to participants. The remuneration amount was designed to balance adequate compensation for time without being too large of an amount to be coercive. Participants will also be reminded that study participation is voluntary and that refusing to participate or withdrawing from the study at any time will not result in any negative consequences on their child's care at LCH.

Participants will be allocated ample time to review the verbal informational handout and/or the written informed consent prior to data collection beginning and will be provided time to ask the Research Assistant questions as well. After all questions are answered to the satisfaction of the participant, and after he/she provides verbal consent and/or signs the consent document, then data collection will begin. Participants will be given a copy of the informational handout and/or the written informed consent form.

4.6 Vulnerable Populations:

Caregivers involved in the study may be pregnant. We will not inquire about pregnancy status. The participation of pregnant women involves no more than minimal risk to the mother, with no foreseeable direct risks to the fetus. Children of caregivers enrolled in the study will be subjects to the extent that they will have information collected about their weight and height at ED

recruitment and follow-up. In addition, images of the children will be captured in photographs of their child passenger safety behaviors, including seating location and passenger restraint system use. Identifying features of the child (e.g., face) will be obscured in the images before they are saved for analysis. The information captured in photographs is needed to determine if the child is using the size-appropriate CRS and to identify errors in CRS use. Other special vulnerable populations (including fetuses, prisoners, and institutionalized individuals) will not be subjects of the proposed research.

5 Study Procedures

5.1 Study Intervention or Observational Phase

5.1.1 Visit 1 (baseline visit)

Screening: Caregivers of children who are eligible to approach will complete a self-administered computerized screening survey on a study iPad or remotely via their own personal device to determine eligibility for the full study.

Caregivers who complete the screening survey and meet study eligibility requirements will have the option to enroll in the full study. Eligible caregivers will participate in the informed consent process before determining if they would like to enroll.

Eligible caregivers that enroll in the study will complete the following elements while in the ED or will have the option to finish any of these elements within 5 business days of enrollment remotely via phone.

- A self-administer a computerized baseline survey with validated items collecting information on their child passenger safety practices with their child, barriers/challenges they may experience in using the recommended child restraint, and motivations, etc.
- Tiny Cargo check-in (a car seat check) with the RA to directly observe and record how their child normally rides in a vehicle. We will use the CPaSS tool to collect data during the Tiny Cargo check-in. Caregiver can submit photos during remote recruitment.
- Randomization to the basic TCBD intervention (an ED counseling session plus eHealth after ED discharge) or the control condition (usual ED care with discharge instructions relevant to the child's diagnosis and an information sheet about car safety).
- Caregivers within the TCBD intervention group --Brief motivational interviewing inspired counseling session (~20 minutes).

5.1.2 Visit 2 –Monthly photo requests (over 12 months of study enrollment)

Over the course of the study, all study participants will receive 8 photo requests in months 1, 2, 3, 5, 7, 8, 10, and 11 via text message from the study that asks for them to send us back digital photographs of how their child usually travels in their vehicle. Participants can complete a total of 8 photo requests over the course of the study and will be paid for completing each of them. The CPaSS measure will be used to assess all digital photographs and incorporates elements of seating position, mode selection, CRS fit, and CRS use/misuse drawn from AAP Guidelines[32] and standardized error severity scores for child restraints.[135]

5.1.3 Visit 3 -- (6 month follow-up visit)

A follow-up assessment will be administered at 6- months after the ED visit and will include both a self-administered computerized survey assessment (~15-20 minutes) and a Tiny Cargo check-in (a car seat check) administered by the RA (~10 minutes). The caregiver will have the

option to complete the 6-month follow up survey in person at the visit or complete the survey on their own device (phone, iPad, laptop, etc.) prior to the follow-up visit. Allowing participants to complete the follow up assessment prior to the visit, will provide convenience to our participants and shorten the amount of time needed for the in person visit. Additionally, if participants move out of state, country or are unable to get to any of the study follow up site locations, then participants will have the option of completing the Tiny Cargo (TC) check-in (a car seat check) by submitting digital photographs to the study staff via text message or completing the check-in via video chat (e.g., Northwestern zoom, Microsoft teams, skype, duo, google hangout).Caregivers that complete the TC check-in via photo submission or video chat will receive $5 less in study incentives. The CPaSS tool will be used to collect data during the TC check-ins.

During the 6-month follow-up, we will also plan to administer a brief health literacy assessment, called the Newest Vital Sign. This valid and reliable tool will help us identify participants at risk for low health literacy in the study. This survey assessment will be self-administered and will last about 3 minutes.

5.1.4 End of Study Visit – (12 month follow-up visit)

The last follow-up assessment will be administered at 12- months after the ED visit and will include both a self-administered computerized survey assessment (~15-20 minutes) and a TC check-in (a car seat check) administered by the RC (~10 minutes). The caregiver will have the option to complete the 12-month follow up survey in person at the visit or complete the survey on their own device (phone, iPad, laptop, etc.) prior to the follow-up visit. Allowing participants to complete the follow up assessment prior to the visit, will provide convenience to our participants and shorten the amount of time needed for the in person visit. Additionally, if participants move out of state, country or are unable to get to any of the study follow up site locations, then participants will have the option of completing the TC check-in (a car seat check) by submitting digital photographs to the study staff via text message or completing the check-in via video chat (e.g., Northwestern zoom, Microsoft teams, skype, duo, google hangout). Caregivers that complete the TC check-in via photo submission or video chat will receive $5 less in study incentives. The CPaSS tool will be used to collect data during the TC check-ins.

<u>Follow-up protocol –retention of subjects</u>

Retention will be maximized with established procedures from our prior work (80-90% compliance). The following protocol will be implemented to improve compliance with follow-up: 1) during the baseline assessment, the participant's name, address, e-mail account, phone number, Facebook and other social media accounts, and telephone numbers of two contact persons will be obtained; 2) reminder letters, telephone calls, text messaging, e-mail communication, and/or Facebook/other social media private messaging will be used for participants who agree to be contacted by such methods; 3) follow-up interviews will be scheduled at the participant's convenience including evenings and weekends; and 4) participants will receive an extra $5.00 for confirming their follow up appointment or calling to reschedule it.

Additionally, we have set up two study e-mail accounts (TCBDstudy@luriechildrens.org and TCBDstudy@gmail.com) to be used for subjects to contact study staff or for study staff to send appointment reminders to subjects. Through the study email account, we will also set up a Facebook and Instagram limited profile page. The Facebook and Instagram profile page lists only the study name and affiliation with LCH. Facebook or Instagram will NOT be used as a recruitment tool. The account is set up only as a source for private messaging between study staff and enrolled subjects who agree to be contacted via such methods. The message shows

up in the person's private inbox, similar to an e-mail message. There is no ability to "friend" the study or post on the wall. Only subjects with active Facebook and Instagram accounts can be messaged. Privacy settings are checked on a monthly basis. When providing contact information, the subject will indicate if study staff can contact them through email, Facebook or Instagram.

5.2 Subject Withdrawal

Participants (caregivers/parents) can stop taking part in this study at any time. Their decision will not affect their regular care or their child's care at LCH. If they wish to stop taking part in this study, we ask that participants call the study team at (312) 339-0888 or email us at TCBDstudy@luriechildrens.org. A study team member can speak to them about any concerns they may have. The study will then confirm their withdrawal from the study with a letter in the mail.

5.3 Early Termination Visits

Participants can stop taking part in this study at any time. Their decision will not affect their regular care or their child's care. If they wish to stop taking part in this study, we ask that participants call the study team at (312) 339-0888 or email us at TCBDstudy@luriechildrens.org. A study team member can speak to them about any concerns they may have. The study will then confirm their withdrawal from the study with a letter in the mail.

6 Statistical Plan

6.1 Sample Size and Power Determination

Briefly, our calculations are based on two-sample comparisons between groups (e.g., control and TCBD) using results from our pilot study that assessed percent size-appropriate child passenger safety behaviors at 6-month follow-up (referred to as the "response rate" below). For analyses involving multiple time points, these calculations represent a conservative lower bound on the power that would only be achieved if the effective sample equals 1 observation per person (i.e. perfect within-individual correlation). For the calculations below we assume: a) 17% response rate among controls; b) 25% response rate among TCBD caregivers at 6 Month (with 75% of TCBD caregivers re-randomized); and c) only 80% of the target sample (n=204 controls and n=516 TCBD in the initial dichotomy) will be available for analysis at follow-up. Components (a) and (b) are based on pilot data where the control group received generic educational materials and outcomes were assessed at 6-months. Component (c) is a conservative correction for attrition. For Aim 2, we estimated power while accounting for uncertainty in the group sizes by simulating
response and re-randomization patterns according to the above probabilities and testing for significant between-group differences; this process was repeated 10,000 times and the power was estimated as the percent of replications in which a between-group difference was identified. Binary Outcome: For the binary response case, if the observed response rate is at least 29.4% among those receiving enhanced TCBD, it can be reliably (>80% power) distinguished from a control response rate of 17%. Analogously, a response rate of at least 27.6% among basic intervention would be reliably (>80% power) distinguished from control. Converted to Cohen's h, both differences reflect small-to-medium effect sizes (h = 0.30 and h = 0.26, respectively). Numeric Outcome (CPaSS): If the standardized mean difference (i.e. Cohen's D) is >0.25 (a small-to-medium effect size), we can reliably distinguish between control and basic TCBD conditions. Similarly, if D ≥ 0.28, a small-to-medium effect size, we will have sufficient power (80%) to distinguish the control and enhanced TCBD conditions. Aim 2: Monte Carlo studies of linear latent trajectory models indicate that a medium level of heterogeneity among the slopes and/or intercepts of the trajectories can be reliably recovered (power > 80%) with a total sample

size of n=200, with power exceeding 95% when the level of heterogeneity is high;190 given that our sample size soundly exceeds 200, we expect sufficient ability to determine the underlying characteristic trajectory types. Nevertheless, we further justify our sample size with a brief simulation study. Assuming a final sample size of 720, conservatively assuming individuals are only measured at an average of 25% of the 12 monthly assessments, we simulate data from a population with four equally likely trajectory types: consistently low, consistently high, linearly decreasing, and starting high then dropping off. Because we have no basis for estimating variability in the score, we assumed mean/variance equivalence, consistent with the Poisson distribution. Due to the time required to fit such models, we only completed 200 simulation replications, where we were able to correctly conclude (based on BIC) that there were four trajectory types in 100% of replications, and the mean trajectories in those four groups were characterized correctly.

Exploratory Aim: Aligning with the NIH guidance on health economics research, we plan a cost analysis of the intervention to inform wider dissemination and implementation in settings outside of the ED. Unmeasured time costs for staff or for participants can be a key barrier to the implementation and long-term viability of a new intervention since preventive and screening recommendations can compete for implementation time from staff. We will address this potential barrier by measuring all relevant costs associated with the study interventions through primary data collection. We will measure the total costs of the intervention from participant and health system perspectives by gathering estimates of intervention resource requirements, including personnel costs, implementation time, participant time, and material costs. Resource costs will include intervention development, training, and implementation. All costs will be adjusted to US 2017 dollars using the Gross Domestic Product Price Index. Control costs will be adjusted to reflect the proportion of time study participants spend on the app. We will calculate costs of the intervention per child. Net costs (savings) will be calculated by subtracting mean costs per participant in the control condition from mean costs per participant who receive the basic TCBD and the enhanced TCBD interventions. Given typical skewness, we will report means, medians, standard errors, minimum, maximum, and quantiles to provide information on cost measures. Tests will be employed to assess distributions of cost measures and identify appropriate methods for generating confidence intervals, such as non-parametric bootstrapping. We will also estimate cost-effectiveness as measured by dollars per caregiver using a size-appropriate CRS and using the CRS correctly. Economic data will provide key information about the relative costs and benefits associated with the intervention, inform scalability, and help prioritize future implementation strategies in EDs and other settings that serve young children (e.g., doctor's offices, schools).

6.2 Statistical Methods

All subjects, once randomized, will be included in an intention-to-treat analysis and outcomes will be collected even if the caregiver does not engage in the randomly assigned intervention. We will evaluate randomization by testing for covariate (e.g. demographic characteristics) balance between initial treatment groups, and between the re-randomized treatment groups created after the 6-month follow-up. If significant differences are observed, we will adjust for these variables or conduct stratified analyses. All statistical hypothesis tests and power calculations will use a two-sided = 0.05.

Aim 1: To test the efficacy of the TCBD intervention compared to usual care for improving primary outcomes [i.e., size-appropriate restraint (dichotomous) and CPaSS (numeric)] at 12 months. We hypothesize greater proportions of children will be using a size-appropriate restraint and higher CPaSS among caregivers in the intervention groups (basic and enhanced) compared with the control group (usual care) at the 12-month assessment. Statistical Analysis:

Our primary analytic tool for estimating treatment effects will be generalized linear models (GLMs), and for analyses involving joint analysis of multiple follow-up data points, we will use generalized linear mixed models (GLMMs), which allows direct modeling of the correlations between data points (e.g. repeated measurements on individuals), and the inclusion of individuals with incomplete data (e.g. missing time points). The GLM(M) framework also allows simple controlling for confounders (if necessary) through their inclusion in the model as covariates. For the analysis of binary outcomes, we will use logistic GLM(M)s and for numeric outcomes (CPaSS-D) we will initially specify linear (Gaussian) GLM(M)s; this choice will be scrutinized by analyzing model diagnostics. The primary inferential target will be the coefficient for the treatment group indicator, making this effectively equivalent to ANCOVA. Adjustment for the baseline measurement is important for guarding against regression to the mean in RCT studies, which will be our approach when analyzing only a single follow-up (e.g. 12 months). However, when analyzing multiple follow-ups within the same model, using this baseline adjustment across the board (i.e. at every time point) can result in overestimation of the treatment effect; baseline adjustment for only the first follow-up corrects this overestimation while also guarding against regression to the mean, which will be our approach. We will also conduct exploratory tests of the interaction between time and treatment group, to see whether there is strong evidence that the treatment effect diminishes over time, which will inform future studies.

SubAim: We will determine moderators (caregiver sex, race, child age) of the intervention effects and mediators (Self-Determination Theory constructs of autonomy, relatedness, competence; emotional state at ED recruitment). We hypothesize that disparities in CRS use can be reduced through a tailored intervention that increases autonomous motivation, competence, and relatedness for correct CRS use. Statistical Analysis: Moderation: Within the GLM(M) modeling framework described above, we will evaluate moderation by including interactions between treatment group and sex-, race-, and age-based groupings. We will test whether sex as a biologic variable modifies the intervention effect, but will be underpowered to detect differences because children are infrequently with a male caregiver when seeking emergency care (10-15% in our pilot data). Mediation: We will use Imai's counterfactual mediation method, a very general framework that takes models for the outcome and mediator as inputs, to estimate mediation effects. Traditional mediation analysis can be susceptible to bias due to model misspecification and design limitations and limitations in the classes of statistical models, that can be accommodated (typically linear regressions for both the mediator and outcome) but the Imai framework can accommodate a wide variety of distributions for both the mediator and the outcome; our initial specification will be a logistic model for the binary variables and a linear (Gaussian) model for the numeric variables. We will use a quasi-Bayesian approach to draw from conditional distribution of the mediator given the "treatment" and other covariates, and that of the outcome given the exposure, mediator, and other covariates, to construct hypothesis tests for the proportion mediated; this approach is robustly implemented in the R package mediation.

Aim 2: To use latent growth curve models to identify characteristic trajectories for the CPaSS observed in digital photographs over the 12 study months and assess for correlates of the trajectory types. We hypothesize that caregivers will have different CPaSS trajectories in response to the basic intervention, the enhanced intervention, and the control condition. We expect to identify at least four patterns of response. Statistical Analysis: We will determine 12-month CPaSS trajectory types using adaptations of finite mixture models for the case where the units are trajectories. We will define trajectory shapes through methods combining semiparametric regression modeling (e.g., generalized additive models), with latent class regression, using the R package flexmix. Following convention, we will use the Bayesian

Information Criteria (BIC) to determine the appropriate number of latent classes, while also making practical considerations to avoid overfitting, such as not allowing overly small groups to be created, as in previous applied work. Using the posterior probability of class membership, we place individuals into trajectory groups and treat that grouping as the outcome in a subsequent multinomial regression model to determine correlates of class membership, including treatment group, caregiver race/ethnicity, and child age group. If substantial uncertainty in class membership is found, outcomes in the multinomial model will be weighted accordingly.

7 Safety and Adverse Events

Dr. Macy will be responsible for reporting Adverse Events and Other Reportable Information and Occurrences to the Lurie Children's Hospital IRB and the Project Officer for this grant as required and as applicable to the study protocol. The timing of reporting of an adverse event by Dr. Macy will be dependent on the severity of the event and whether the adverse event was expected (included in the informed consent).

7.1.1 Data and Safety Monitoring Plan

The Principal Investigator, Dr. Macy will be responsible for the data safety and quality in this low risk trial of a behavioral intervention to promote correct and consistent size-appropriate child passenger safety behaviors. It should be noted that all research project involving human participants at Ann & Robert H. Lurie Children's Hospital of Chicago, including the proposed one, require approvals from the Lurie Children's Hospital Institutional Review Board. In addition, because of the potential for sensitive data collection using digital photographs, a Certificate of Confidentiality will be obtained for this study from The Eunice Kennedy Shriver National Institute of Child Health and Human Development.

Dr. Macy will ensure that all relevant IRB policies, procedures and stipulations are followed. She will be responsible for ensuring that other investigators and project staff adhere to IRB policies including (1) All participants will understand, agree to and sign a written consent forms before participating; (2) strict adherence to a participant's right to withdraw or refuse to answer questions will be maintained; (3) the questionnaires will be confidential and no names will be associated with the survey data; (4) consent forms and identifying information will be kept separate from the participants' study data; (5) all identifying information in digital form (e.g., follow-up tracking data) will be encrypted and saved on password-protected devices and stored in a locked filing cabinet. All identifying information on paper (e.g., consent documents) will be stored in a locked filing cabinet. Computers using de-identified data for analysis will be password protected; and (6) participants will be informed in the consent form with information to contact the PI, the project manager, and the IRB office with any questions and/or concerns. Dr. Macy will be responsible for submitting any amendments to the IRB before making changes to the study protocol.

Dr. Macy will be responsible for providing training to all research staff conducting assessments regarding procedures for managing anticipated issues. Specifically, this training will include information regarding warning signs of distress that occur as a result of the surveys and the means for addressing such issues and minimizing distress. Staff will receive training from Dr. Macy in management procedures if a caregiver reveals child abuse/neglect or concerns about safety. Staff training will include review of the study protocol regarding the limits of confidentiality, liaison with ED staff (i.e., clinical care providers) regarding concerns, and circumstances in which it may be necessary to report suspected child abuse or neglect to the Illinois Department of Children and Family Services (DCFS). Study procedures will also require staff to immediately contact Dr. Macy in cases where these concerns arise. Dr. Resnicow will be responsible for training in Motivational Interviewing (MI) techniques. The project manager will

facilitate monitoring for fidelity to the intervention throughout the course of the study by reviewing and scoring audio recordings from MI sessions. A consultant with mastery of MI techniques and teaching of MI will provide feedback and additional training as needed.

The digital photographs submitted by caregivers will be reviewed by a trained research assistant within 24-48 hours of the submission. The research assistant will score the photographs with the Child Passenger Safety Score and determine which feedback messages will be returned to the caregivers in the intervention group. For the development of the CPaSS Tool, in collaboration with Dr. Reed and child passenger safety technicians, Dr. Macy has developed a list of child passenger safety behaviors that constitute serious misuse (for example, having a car seat sitting on the vehicle seat but not attached in any way). To offer greater protection in a crash to all children involved in the study whose caregivers have not obtained a size-appropriate child restraint system at the 6- or 12-month follow-up assessment, we have budgeted to provide size-appropriate restraints at no cost to the participants at the conclusion of the study. To receive a CRS from the study, we will require caregivers to have the restraint installed with the guidance of a certified child passenger safety technician. This will ensure the restraint is installed in accordance with manufacturer specified guidelines.

Dr. Macy will be responsible for reporting Adverse Events and Other Reportable Information and Occurrences to the Lurie Children's Hospital IRB and the Project Officer for this grant as required. The timing of reporting of an adverse event by Dr. Macy will be dependent on the severity of the event and whether the adverse event was expected (included in the informed consent).

7.1.1.1 Data Safety Monitoring Board

Dr. Macy will work with 2 certified child passenger safety technicians to form a data safety monitoring board. If serious misuse is identified, caregivers will receive pre-determined corrective text messages regardless of the treatment condition. If the research assistant has any questions about the potential for a serious misuse, the photograph will be brought to the attention of the data safety monitoring board and reviewed for determination of serious misuse. Communication to caregivers about serious misuse will occur within 2 days of receipt of the photographs by the study team. Illegal behavior depicted in photographs will be protected within the legal rights provided by the Certificate of Confidentiality.

8 Study Administration, Data Handling and Record Keeping

8.1 Confidentiality

Information about study subjects will be kept confidential and managed according to the requirements of the Health Insurance Portability and Accountability Act of 1996 (HIPAA). Those regulations require a signed subject authorization informing the subject of the following:

- What protected health information (PHI) will be collected from subjects in this study
- Who will have access to that information and why
- Who will use or disclose that information
- The rights of a research subject to revoke their authorization for use of their PHI.

All research staff assisting with data collection, entry and analysis will receive training in the appropriate data handling and confidentiality procedures. Staff will be made aware that a breach of confidentiality is reason for dismissal.

8.2 Data Collection and Management

Data will be collected only after written informed consent has been obtained from the participant.

Six primary sources of data exist for this study:

1) Screening process completed by the research coordinator
The screening process will use information that can identify the child of the caregiver participating. The identifiable information that will be collected and stored in the screening/recruitment log and enrollment log is the child's name, medical record number, date of birth, and date of being screened for study. The investigators need to collect this information because it will help us to avoid approaching families who have already participated in the study or decided not to participate. These tracking logs will be stored on an encrypted hospital device and will not be shared with persons not on the study team unless a Lurie HIPAA release form has been signed.

2) Surveys (screen, baseline, 6- and 12-month follow-ups) completed by the participant
Caregivers will be approached for screening and baseline survey discretely in the ED waiting room or privately in their child's ED treatment room. We will use a private area, separate from other patients in the ED, for review of the part 1 informational handout and the part 2 informed consent document. Caregivers will be informed of the time commitment and activity on their part necessary for the completion of the research project. Data collection in the ED will be completed privately and confidentially on a study iPad and can be self-administered unless the caregiver prefers to have the research assistant administer the survey verbally. If the survey is administered verbally, the necessary measures will be taken to ensure privacy of response options of the caregiver. Follow-up assessments similarly will be self-administered on study iPads during the 6- or 12-month follow-up visit, unless the participant chooses to complete the follow-up survey prior to the follow -up visit. Then the participant will complete the survey at their convenience and on their own device (e.g., phone, laptop, iPad, etc.).

Children of caregivers enrolled in the study will have information collected about their weight and height at ED recruitment and in-person follow-up assessments. The children will be receiving emergency medical care in the LCH ED. All research staff who will have interactions with children will receive training from Dr. Macy, a board-certified pediatrician and pediatric emergency physician, related to their interactions with children for the measurement of weight and height as well as the direct observation of the child in the vehicle which they travelled to the ED and at follow-up assessments.

Subject identifiers (i.e., subject ID numbers) will be present on all surveys to link their data to their identifying information. The Qualtrics Research Suite (http://www.qualtrics.com/) will be used to collect and store data from the screening survey and part of the 6 month follow-up survey (The Newest Vital Sign survey). The project-specific Qualtrics site is protected with a researcher-designated log-in name and password. The site houses the surveys and compiles data that staff will then transfer to a secured database on the LCH secure network. There are systems in place that prevent the survey from being taken by the same user more than once. No identifying information is collected from participants; so information remains anonymous and in aggregate form. Additionally, Qualtrics has SAS 70 Certification and meets the rigorous privacy standards imposed on health care records by the Health Insurance Portability and Accountability Act (HIPAA). All Qualtrics accounts are hidden behind passwords and all data is protected with real-time data replication. Qualtrics complies with the U.S. and E.U. Safe Harbor Framework and the U.S. and Swiss Safe Harbor Framework as set forth by the U.S.

Department of Commerce regarding the collection, use and retention of personal information from European Union member countries and Switzerland. Qualtrics has certified that it adheres to the Safe Harbor Privacy Principles of notice, choice, onward transfer, security, data integrity, access, and enforcement.

Data obtained in the baseline and follow-up surveys will be stored on a password protected, secure server at the University of Michigan. The University of Michigan Center for Health Communications Research (CHCR) has been subcontracted for this R01 to program the surveys, the study website, and the tailoring of web content using the Michigan Tailoring System (MTS) open-source software. MTS does not have any sort of central server that communicates back and forth with a intervention web "site" – so there is minimal security risk in adding it to any project. MTS functions where it takes code generated by CHCR and then uses a library to interpret how to correctly display that code on the intervention site. Baseline, follow-up and intervention data will be coded according to participant ID number. Additionally, a formal review of the survey system and study web "site" is being completed by the University of Michigan Information Assurance Office (https://safecomputing.umich.edu/about). Once completed, the information assurance assessment will be shared with the Information Management and Institutional Review Board teams at Lurie Children’s.

All data collected through the University of Michigan and stored on University of Michigan password protected secure servers will be accessible to the study team via a dashboard. Data will be downloaded to password protected secure servers at Lurie Children’s for analysis. We will develop databases for the storage of de-identified data. Files will be stored and backed-up using password protected secure file servers at LCH. Multiple imputation may be employed to address missing data due to dropout or inability to complete follow-up (anticipated <20%).

We will minimize the burden of the research assessments through the use of skip patterns. In order to minimize pressure to provide the perceived “correct” answer, caregivers will be reminded there are no “right” or “wrong” answers to any of the assessment questions. Caregivers will also be informed that they have the right to skip any question that makes them uncomfortable or that they do not wish to answer. They will be informed of their right to withdraw from the study at any time without penalty. Study staff will be trained to respond to emotional distress and to discuss concerns and issues with the project manager, Dr. Macy, and in her absence, Dr. Carter or Dr. Resnicow.

We will also have a Certificate of Confidentiality (CoC) from The Eunice Kennedy Shriver National Institute of Child Health and Human Development to protect study participants’ data regarding illegal behaviors captured within any of the survey from subpoena and to conserve their own right to request release of their data for their own purposes.

Data collection from all surveys will occur electronically according to the information presented above, unless there are technical problems during the study. To account for technical issues, paper copies of surveys will be used to complete any data collection needed with participants. Data collected via paper will be transferred to the secure online systems as soon as possible by a trained research team member and the paper documents will be shredded afterwards.

Subject identifiers (i.e., subject ID numbers) will be present on all surveys. The subject identifiers will be used to link information to each participant as opposed to using unique identifying information. When patients enroll in the part 2, baseline phase of the study, names will be linked to individual ID numbers in this secure online database created by CHCR, which will be kept in a restricted access folder on a secure server at the University of Michigan. Data

exported from the University of Michigan servers for future data analysis will be de-identified before saving to a password-protected folder on secure network at LCH.

3) Monthly photo submissions and text messages from caregivers of their child as they normally ride in their vehicle

Caregivers will submit photos of their child as they normally ride in their vehicle once a month to the study team. Text messages will be sent and photographs submitted to the study team through a third party platform called Twilio (Twilio Inc., San Francisco, CA) (see www.twilio.com for information about Twilio and its privacy/security policies). Twilio will transmit messages and store links to the MMS (photograph submission) and SMS (text message) content on Twilio servers. Twilio will save the image files to an AWS Server. The Twilio link to the MMS message files will be deleted with an API command sent each day at midnight Eastern Time. MMS files are deleted from the AWS Server after 30 days but will not be searchable after the MMS file link is deleted at midnight. SMS file records will be deleted with an API command sent at the conclusion of the caregiver's participation in the study (i.e., 1 month after completion of the 12-month follow-up). This will ensure we have a complete record of communication sent to them by the study team.

The study team will make every effort to keep data safe and secure. The University of Michigan Center for Health Communications Research has been subcontracted for work on this project. A formal review of the study website is being completed by the University of Michigan Information Assurance Office (https://safecomputing.umich.edu/about). Data submitted to Twilio will be transmitted by Twilio to the University of Michigan and saved at the University of Michigan in password-protected folders on a secure network. Only study team members will have access to data.

Data collected for the study through the University of Michigan systems will be downloaded to Lurie Children's and stored in a password protected folder on a password-protected, secure network. All photos will be de-identified – meaning faces will be blurred – when saved to Lurie Children's network. Photos will be deleted from the University of Michigan secure server once they are successfully saved to Lurie Children's network.

Tango Card*- The gift card distribution will be handled using Tango Card™, an electronic gift card distribution software. Tango Card™ will send an email to participants each time a gift card is earned for this study. They will be able to redeem the gift card at a major retailer of their choice. Tango Card™ does not sell or distribute customer email addresses to any third party and Tango Card™ treats all personal data as completely confidential.*

We will also have a Certificate of Confidentiality (CoC) from The Eunice Kennedy Shriver National Institute of Child Health and Human Development to protect study participants' data regarding illegal behaviors captured within the digital photographs from subpoena and to conserve their own right to request release of their data for their own purposes. The CoC will protect all digital photographs collected during the study.

4) Child Passenger Safety Score (CPaSS-D) and (CPaSS-R) used by the research assistant for direct and remote observation of the Tiny Cargo check-ins (car seat inspection)

The CPaSS electronic survey tool will be used by research assistants to observe and record assessment scores for the Tiny Cargo check-ins each month that a participant is enrolled in the

study. The CPaSS tool will be used either in direct observation with a family or via photos submitted electronically via text message to the study team.

CPaSS observations recorded from review of the digital photographs will be collected in surveys on the Northwestern University portal to the Qualtrics survey platform that contain study-specific codes only. Qualtrics will house the data for the CPaSS assessments and will only use a subject identifier (ID number) to connect the participant's data with their information. No unique identifying information will be collected within this assessment tool. When it is exported from Qualtrics, the data will be saved to a secure folder on the LCH secure network.

5) Audio recordings of the MI counseling sessions (conducted at the baseline visit, and/or Month 7/8 via phone)
Although the focus of this study is not on child abuse, because of the nature of the study, caregivers may disclose information that raises concerns for potential child abuse or neglect during the MI counseling sessions (baseline visit and/or phone session). The consent form will contain a statement regarding the mandatory reporting requirements. Staff training will include review of the study protocol regarding the limits of confidentiality, liaison with ED staff (i.e., clinical care providers) regarding concerns for potential child abuse or neglect, and circumstances in which it may be necessary to report suspected child abuse or neglect to Illinois Department of Children and Family Services (DCFS). Study procedures will also require staff to immediately contact Dr. Macy in cases where these concerns arise.

MI counseling sessions for this study will be conducted/facilitated by the RA with participants and will be audio recorded (with signed permission from participants) in order to ensure the fidelity of the intervention. Staff will be trained to administer the TCBD MI counseling intervention in a specific way and in order to ensure all staff are conducting and coding the data correctly, it is necessary to audio record this portion of the study. Audio recordings of MI counseling sessions will not include the caregiver's name or any information that could be used to identify them. Audio records will be coded only with the research assistant ID, subject ID and study name, and date of the session. Taping will be done using a LCH secure device (study iPad and/or study iPhone), and counseling sessions will be immediately uploaded to a password protected LCH server folder and deleted from the device.   All audio recordings will be destroyed from the LCH server as soon as fidelity monitoring is completed.

6) Paper study documents --consent forms, proof of payment and study follow-up information
All paper study documents (e.g., consent forms, payment forms, follow-up related contact forms, etc.) with patient identifying information will be kept stored in locked file cabinets separate from any data collection forms. Each file cabinet will have a unique key and will be kept within a locked office, within a locked suite and within a locked building accessible by LCH badge only.

Subject identifiers (i.e., subject ID numbers) will be present on study-related forms. The subject identifiers will be used to link information to each participant. When patients enroll in the part 2, baseline phase of the study, names will be linked to individual ID numbers in this database, which will be kept in a restricted access folder on a secure server.

Signed consent forms will be kept in a locked file cabinet in a locked research office at the study site. All information collected as part of both research projects will be accessible only to research staff who have completed mandatory training in the protection of human subjects.

Follow-up contact information that the caregiver provides to the study team will be stored separately with a unique study identifier to link information. The dataset with identifiers will be

destroyed as soon as possible after the completion of 12-month follow-up. Caregiver identifiers (contact information) will be retained for the purposes of follow-up.

The study team will follow the federal guidelines as the necessary time that all study materials will kept following the conclusion of the study recruitment and analysis period.

Blinding for purposes of data collection:
This is a unique single-blinded study where a portion of the study will include blinding of study team members. The blinding will not include the Principal Investigator or the Study Coordinator. Also, participants will know which group they are enrolled in as the consent form states the study groups as well as the extent of study involvement based on which group you are enrolled in.

The portion of the study that includes blinding are the monthly photo submissions that participants will send us via text message over the course of 12 months.
Study team members that directly enroll participants in the ED will not be able to review any monthly photo submissions from those families. They will only be able to review and score (using the CPaSS tool) photo submissions of families that they did not directly enroll, so they are blinded to which study arm the family is enrolled in.

Study team members that are only assisting with the review and scoring (using the CPaSS tool) of photo submissions each month will be blinded to the study arm the participant is enrolled within. Because they will not be enrolling participants within the ED and they will not have access to the study dashboard where the photos are submitted, these study team members will remain blinded throughout the course of the study and will only provide scoring based on what they observe within the photos. These study team members will relay the scoring of all photo submissions to the rest of the team to record within the database and the additional study team members will send out text message feedback to the families based on any major misuse/errors that are visible within the photo submissions.

8.3 Records Retention

Study investigators will follow the federal guidelines as to how long all data collection and records should be retained past study conclusion.

9 Study Monitoring, Auditing, and Inspecting

9.1 Risks

Potential Risks:
Physical: None anticipated

Psychological: There is no more than minimal risk that caregivers may experience stress associated with seeking ED care for their child and may find the intervention inconvenient. Participants may become anxious, upset, or uncomfortable as a result of being asked personal questions. There is a theoretical risk that participants may feel pressure to provide the “correct” answer. Participants will be reassured that there are no right or wrong answers to the study questionnaires, that their answers are confidential, and that the data will be made anonymous by removing all potential identifiers. Children may become anxious with the measurement of their weight and height by a research staff member who is unfamiliar to them. Children will not be separated from their caregivers at any time during the study.

Social: Because the research requires the collection of personally identifying data, there is always a risk of breach of confidentiality. This risk is no greater than that which occurs in every medical encounter. We will make every attempt to minimize that risk. Only the principal investigator, Dr. Macy, project manager, and research assistants will have access to personally identifying contact information for the enrolled caregivers, which will be destroyed at the earliest possible time. The photographs collected in the course of this study will be stored separate from the other data (survey questions and measures) in a secure LCH server accessible to the principal investigator, Dr. Macy, and the research assistants responsible for coding the photographs.

Breach of Data: Accidental public release of the survey data or text messages (SMS or MMS) may occur due to unplanned data breaches including hacking or other activities outside of the procedures approved by this study. If a security break happens, their data may be misused or used for unapproved reasons.

Financial: None anticipated

Legal: Caregivers will be made aware that spontaneous disclosure of behaviors concerning for child abuse or neglect (not directly assessed in the study) will be reported to the Illinois Department of Children and Family Services (DCFS). Caregivers also may fear legal repercussions for reporting their child passenger restraint practices if they do not adhere to the state law. We have determined, in consultation with experts from the Child Abuse Team at Lurie Children's Hospital that, while illegal, it is not considered child abuse or neglect in the State of Illinois to use a restraint other than a legally required restraint, unless the child were injured as a result of the improper restraint.

Identification of safety hazards: There is always the potential for human error and for the research coordinator to miss identifying a major misuse or error in the way the participant's child is using their child restraint system.

Adequacy of Protection against Risks:

Recruitment and Informed Consent

All study procedures, protocols, measures, and informed consent documents will be reviewed and approved by the Lurie Children's Hospital Institutional Review Boards before any activities involving human subjects begin. Waiver of informed consent will be requested for review of the ED Electronic Medical Record (EMR)/Patient Tracking board to determine which families to approach for screening. Adult caregivers of children 6 months to 10 years old, who bring their child to the LCH ED for any non-critical illness or injury, will be approached for eligibility screening. Again, the child age range was selected based on the American Academy of Pediatrics Guidelines for Child Passenger Safety.

Caregivers will provide verbal consent for the ED screening. Eligible caregivers will complete separate written informed consent for 1) participation in the intervention study with baseline/follow-up assessments and 2) the collection of data using digital photographs. Prior to beginning any data collection with a participant, a trained research assistant will present and explain a comprehensive informed consent document, reviewing the study risks and participants' rights. After all questions are answered to the satisfaction of the participant, and after he/she signs the consent document, then data collection will begin. Participants will be given a copy of the consent form(s).

Protections against Risk:

Data collection will be conducted in accordance with Institutional Review Board guidelines for human subjects' protection and patient confidentiality. All research staff assisting with data collection, entry and analysis will receive training in the appropriate data handling and confidentiality procedures. Staff will be made aware that a breach of confidentiality is reason for dismissal.

Physical: None anticipated

Psychological: We will minimize the burden of the research assessments through the use of skip patterns. In order to minimize pressure to provide the perceived “correct” answer, caregivers will be reminded there are no “right” or “wrong” answers to any of the assessment questions, that their answers are confidential, and that the data will be made anonymous by removing all potential identifiers, only a study ID number will be used. Caregivers will also be informed that they have the right to skip any question that makes them uncomfortable or that they do not wish to answer. They will be informed of their right to withdraw from the study at any time without penalty. Study staff will be trained to respond to emotional distress and to discuss concerns and issues with the project manager, Dr. Macy, and in her absence, Dr. Carter or Dr. Resnicow.

Children may become anxious with the measurement of their height by a research staff member who is unfamiliar to them. Children will not be separated from their caregivers at any time during the study. The child's height will be measured laying down or standing up, depending on what is most comfortable for the child.

Also, caregivers will be approached for screening discretely in the ED waiting room or privately in their child's ED treatment room. We will use a private area, separate from other patients in the ED, for review of the consent document. Caregivers will be informed of the time commitment and activity on their part necessary for the completion of the research project. Data collection in the ED will be completed on a study iPad and can be self-administered unless the caregiver prefers to have the research assistant administer the survey verbally. Follow-up assessments similarly will be administered on study iPads or can be completed remotely via the participant's own personal device and privately, at their convenience.

Social: We will make every attempt to minimize the risk of breach of confidentiality. Only the principal investigator, Dr. Macy, project manager, and research assistants will have access to personally identifying contact information for the enrolled caregivers, which will be destroyed at the earliest possible time. The photographs collected in the course of this study will be stored separate from the other data (survey questions and measures) in a secure LCH server accessible to the principal investigator, Dr. Macy, and the research assistants responsible for coding the photographs.

Also, subject identifiers will not be present on any data collection forms. Follow-up contact information will be stored separately with a unique study identifier to link information. The dataset with identifiers will be destroyed as soon as possible after the completion of 12-month follow-up. Caregiver identifiers (contact information) will be retained for the purposes of follow-up. These identifying data and the digital photographs (with the child's face obscured) will be kept in a secure database on a LCH server accessible from password-protected study computers and archived in a password-protected file on the principal investigators office desktop computer on the LCH network. A separate dataset for analysis will be created, which will contain only unique study identifiers. Audio recordings of MI sessions will be destroyed as soon as fidelity monitoring is completed.

Breach of Data: We have a Certificate of Confidentiality (CoC) from The Eunice Kennedy Shriver National Institute of Child Health and Human Development to protect study participants' data regarding illegal behaviors captured on the digital photographs from subpoena and to conserve their own right to request release of their data for their own purposes. The CoC will protect all digital photographs collected during the study.

Although the focus of this study is not on child abuse, because of the nature of the study, caregivers may disclose information that raises concerns for potential child abuse or neglect. The consent form will contain a statement regarding the mandatory reporting requirements. Staff training will include review of the study protocol regarding the limits of confidentiality, liaison with ED staff (i.e., clinical care providers) regarding concerns for potential child abuse or neglect, and circumstances in which it may be necessary to report suspected child abuse or neglect to Illinois Department of Children and Family Services (DCFS). Study procedures will also require staff to immediately contact Dr. Macy in cases where these concerns.

The study team will make every effort to keep data safe and secure. The University of Michigan Center for Health Communications Research has been subcontracted for work on this project. A formal review of the survey system and study website is being completed by the University of Michigan Information Assurance Office (https://safecomputing.umich.edu/about). Text messages will be sent and photographs submitted to the study team through a third party platform called Twilio (Twilio Inc., San Francisco, CA) (see www.twilio.com for information about Twilio and its privacy/security policies). Twilio will transmit messages and store links to the MMS (photograph submission) and SMS (text message) content on Twilio servers. Twilio will save the image files to an AWS Server. The Twilio link to the MMS message files will be deleted with an API command sent each day at midnight Eastern Time. MMS files are deleted from the AWS Server after 30 days but will not be searchable after the MMS file link is deleted at midnight. SMS file records will be deleted with an API command sent at the conclusion of the caregiver's participation in the study (i.e., 1 month after completion of the 12-month follow-up). This will ensure we have a complete record of communication sent to them by the study team.

Data submitted to Twilio will be transmitted by Twilio to the University of Michigan and saved at the University of Michigan in password-protected folders on a secure network. Only study team members will have access to data.

Data collected for the study through the University of Michigan systems will be downloaded to Lurie Children's and stored on a password-protected, secure network. All photos will be de-identified – meaning faces will be blurred – when saved to Lurie Children's network. Photos will be deleted from the University of Michigan secure server once they are successfully saved to Lurie Children's network.

***Tango Card**- The gift card distribution will be handled using Tango Card™, an electronic gift card distribution software. Tango Card™ will send an email to participants each time a gift card is earned for this study. They will be able to redeem the gift card at a major retailer of their choice. Tango Card™ does not sell or distribute customer email addresses to any third party and Tango Card™ treats all personal data as completely confidential.*

All paper study documents (e.g., consent forms, locator forms) will be stored within locked study file cabinets within a locked office and within a locked suite at Lurie Children's Hospital. All identifiable info will be stored within separate locked file cabinets as well.

Financial: None anticipated.

Legal: We have determined, in consultation with experts from the Child Abuse Team at Lurie Children's Hospital that, while illegal, it is not considered child abuse or neglect in the State of Illinois to use a restraint other than a legally required restraint, unless the child were injured as a result of the improper restraint. Our study team will not be approaching the caregivers of any children seeking care in the ED for a motor vehicle related injury.

Identification of safety hazards: The study team will make every effort to identify misuse and errors in the way that participants are using their child restraint system. In order to ensure the study team is educated on the identification of safety hazards, several training sessions have been conducted with the study team and led by certified child passenger safety technicians at LCH. The Study Coordinator and PI of this study have been certified as child passenger safety technicians as well. They will both oversee study procedures and will consistently review the feedback provided to participants during the Tiny Cargo check-ins (both in-person and via photos submitted by the parents). We will plan to conduct booster training sessions with the study team throughout the course of the study to serve as fidelity checks and keep the study team up to date on the child passenger safety information. We will also plan to refer participants directly to the Lurie Children's Buckle Up Program (car seat program) if any participants have questions or concerns that the study team does not feel they can answer or if the participants' questions fall outside of the training provided for this study. All participants enrolled in this study will also receive a handout with some online resources and phone numbers for places they can get their car seat checked for proper installation by a certified child passenger safety technician.

<u>Third party to whom data will be released:</u>
Data collected as part of the surveys, website, and photo submissions will be released in real-time to the Center for Health Communications Research (CHCR) at the University of Michigan. CHCR is a National Cancer Institute Center of Excellence in Cancer Communications Research since 2003, along with its former embodiments, the Health Media Research Laboratory (University of Michigan) and the Health Communications Research Laboratory (University of North Carolina at Chapel Hill), has conducted research in health communications since 1992. The CHCR comprises an interdisciplinary team of behavioral scientists, health educators, instructional designers, media professionals, computer engineers, graphic designers, project managers and students from a wide variety of disciplines (public health, psychology, medicine, biostatistics, computer engineering, information science, art and others). Principal CHCR staff have worked together for over a decade and have established a shared knowledge base and language regarding tailoring technology and intervention development. Each team member is familiar with the theories and intricacies involved in developing heath behavior interventions and conducting research trials. The interactions among the CHCR's professionals from various disciplines foster the generation of innovative ideas, creative solutions to obstacles and unique perspectives.

CHCR has a significant history of developing and implementing interactive individually tailored interventions as a team, developing and examining the effectiveness of tailored print-telephonic-, computer-, mobile- and web-based interventions. These interventions have used an array of media channels and technologies, including web, computer, print, kiosk, cell phone/mobile device, IVR (interactive voice response), video/DVD and social media applications. This research has been carried out in a variety of settings, including health clinics, emergency departments, primary care offices, schools, homes, inner-city community centers, shopping malls, libraries, work sites and senior centers. Target populations have included patients, physicians, health maintenance organization members, adolescents, the elderly,

minorities, military personnel and the under-served. Topics have included smoking cessation and prevention, dietary change, physical activity, screening, genetic counseling, risk prevention, treatment decision-making, medication adherence and survivorship.

The CHCR-developed Michigan Tailoring System (http://chcr.umich.edu/mts), is a standards-based, cross-platform, open source application released to the research community in 2008 that enables the efficient creation, testing and delivery of richly tailored communications without the need for constant technical support and assistance from a team of computer programmers. These communications may include individually tailored text and media (photos, graphics, animations, audio, video) and be delivered via a range of channels, including print, computer, web, e-mail, cell phone and social media applications. Because of the scalable, common technical platform, resulting tailored programs are easier to disseminate.

Details on how study participant's confidentiality (personal information) will be protected while using the platform(s) for remote study visits:

As LCH has transitioned from using Skype for Business over to the Avaya System, our research team has also made this transition to align with LCH policies and procedures. Like Skype Dialer, Avaya will allow individuals to call research participants from their own cell phone and have their Lurie Children's phone number presented to the recipient of the call.The use of Avaya EC500 Dialer will be used by our research team to:

1) invite parents to be part of the research study
2) complete study visits and study related tasks
3) update parent's information as needed over the course of study
4) provide reminders for upcoming appointments and study related tasks

The following details will be used to protect the confidentiality of the subject while using Avaya Dialer:
1) The parent's identity will be confirmed prior to the transfer of any information over the phone.
2) The Research Coordinator will provide their name and credentials at the start of the call.
3) The parent will be encouraged to move to a quite area where they can be alone during the conversation. They have the option of who overhears their call.
4) The Research Coordinator will move to a location/work space where they are alone and no one else can overhear them or the conversation.
5) The parent has the option of providing information to the Research Coordinator based on their level of comfort.
6) First names will be used during conversations as opposed to full names to protect their identity if anyone overhears the call.
7) Subjects are giving a unique study ID if they enroll in the study.
8) Any information collected during the call is electronically saved to the LCH secure server which is password protected.

While using NU Zoom, the following precautions will be used to protect confidentiality:
1) We will always use a password for a zoom meeting
2) We will always use the waiting room, rather than allowing "join before host" (this prevents unwelcome zoom-crashing)
3) We will always ensure that the interviews are conducted in a private, quiet location that cannot be overheard (applies to both the interviewer and the participant)

4) We will not leave recordings on the Zoom cloud – always save immediately to the shared drive, which is more secure, and gets backed up daily, and then delete them from the Zoom cloud
5) We will have a second study team member confirm the recordings have been deleted from the Zoom cloud.

While using Microsoft Teams (MT) the following will be used to protect confidentiality:
1) Meeting options: With meeting options, you can decide who from outside of your organization can join your meetings directly, and who should wait in the lobby for someone to let them in. PSTN callers will be joining via lobby. Meeting organizers can also remove participants during the meeting.
2) Roles in a meeting: A meeting organizer can define roles in a Teams meeting that designate “presenters” and “attendees,” and control which meeting participants are allowed to present content in the meeting.
3) Attendee consent for recording: All recordings of meetings are accompanied by a notice to attendees that a recording is taking place. The notice also links to the privacy notice for online participants, and the meeting organizer controls which attendees have the ability to record.
4) Meetings recording access: Meeting recording access is limited to those people who are on the call, or invited to the meeting, unless the meeting organizer authorizes others to access the recording. Recordings are uploaded to Microsoft Stream and may be shared and downloaded according to permissions enabled by account administrators.

Details on how study participant’s privacy (undisturbed or observed) will be protected while using the platform(s) for remote study visits:

The following details will be used to protected the study participant's privacy while using Avaya System:
1) The parent will be encouraged to move to a quite area where they can be alone during the conversation. They have the option of who overhears their call.
2) The Research Coordinator will move to a location/work space where they are alone and no one else can overhear them or the conversation.
3) The parent has the option of providing information to the Research Coordinator based on their level of comfort.
4) First names will be used during conversations as opposed to full names to protect their identity if anyone overhears the call.

While using NU Zoom, the following will be used to protect privacy:
1) We will always use a password for a zoom meeting
2) We will always use the waiting room, rather than allowing “join before host” (this prevents unwelcome zoom-crashing)
3) We will always ensure that the interviews are conducted in a private, quiet location that cannot be overheard (applies to both the interviewer and the participant)
4) We will not leave recordings on the Zoom cloud – always save immediately to the shared drive, which is more secure, and gets backed up daily, and then delete them from the Zoom cloud
5) We will have a second study team member confirm the recordings have been deleted from the Zoom cloud.

While using Microsoft Teams, the following will be used to protect privacy:
1) MT never uses the data to serve you ads.
2) MT does not track participant attention or multitasking in Teams meetings.

3) The data is deleted after the termination or expiration of your subscription.
4) MT takes strong measures to ensure access to your data is restricted and carefully define requirements for responding to government requests for data.
5) You can access your own customer data at any time and for any reason.
6) MT offers regular transparency reports on the Transparency Hub, detailing how they have responded to third-party requests for data.
7) MT have taken steps to ensure that there are no back doors and no direct or unfettered government access to your data.

9.2 Benefits

<u>Direct Benefits:</u>
Depending on which group a participant is randomly assigned to, the caregiver may or may not get any direct benefit from taking part in this study. Receipt of the motivational interviewing session and child passenger safety information is likely to increase the caregiver's motivation and competence for the consistent use of the size-appropriate child passenger restraint, which may result in better child occupant protection in the setting of a motor vehicle collision.

Other direct benefits to the participants may include:

- Access to the study's website ("Car Seat Compass") with info on car seat safety.
- A new car seat, if certain conditions are met (e.g., car seat is recalled, expired, or damaged/missing parts, participant's only reported reason for not using a child restraint system is insufficient finances)
- Feedback from a study team member about safer ways to travel with their child. They may get this feedback via text message or verbally from a staff member.
- A private counseling session with a Car Seat Coach (in the ER or via phone). They would discuss how they feel about car seats and how their child usually rides while in the car.

<u>Indirect Benefits:</u>
The proposed research will potentially benefit others by leading to an effective intervention for the promotion of size-appropriate child passenger safety behaviors.

Injury remains the leading cause of death among children after the first year of life. Motor vehicle occupant-related injuries are the top-ranking cause of unintentional injury deaths among children older than 4 years. Car seats, booster seats, and seat belts are proven effective for reducing the risk of injury and death in a motor vehicle collision. However, appropriate child passenger restraint use remains below targets such as those stated in the Health People 2020 Objectives. Finding ways to ensure that every child is using the size-appropriate child passenger restraint on every trip is one of the most critical injury prevention needs today, yet few effective evidence-based precision prevention approaches exist to reach the caregivers who practice suboptimal child passenger safety behaviors. By identifying at-risk families during a child's ED visit, the proposed intervention could remarkably enhance the universal injury prevention efforts already in place in communities and physician offices. As such, the knowledge gained from this study is of vital importance to public health efforts for childhood injury prevention.

9.3 Risk Benefit Assessment

There is no more than minimal risk that caregivers may experience stress associated with seeking ED care for their child and may find the intervention inconvenient. Participants may become anxious, upset, or uncomfortable as a result of being asked personal questions. There

is a theoretical risk that participants may feel pressure to provide the “correct” answer. Participants will be reassured that there are no right or wrong answers to the study questionnaires, that their answers are confidential, and that the data will be made anonymous by removing all potential identifiers. The risk of a potential data breach is offset by the potential for new knowledge about promoting child passenger safety best practices. Children may become anxious with the measurement of their weight and height by a research staff member who is unfamiliar to them. Children will not be separated from their caregivers at any time in the course of the study for study related purposes. Children may need to be separated from their caregivers as part of their clinical care in the ER.

9.4 Informed Consent Process / HIPAA Authorization

All caregivers will provide verbal consent for the completion of the screening survey.. Verbal consent will be obtained using an informational sheet template for waiver of obtaining a signed consent form from Lurie Children’s IRB. This sheet provides sufficient information for subjects to make an informed decision about their participation in this study. Caregivers who screen eligible for study enrollment after completing the screening survey will then complete a separate informed consent discussion with trained study staff and this process will include written documentation of consent to the 1) participation in the randomized control trial of the intervention with baseline/follow-up assessments and 2) collection of data using digital photographs.

Prior to beginning any data collection with a participant, a trained research assistant will present and explain a comprehensive informed consent document, reviewing the study risks and participants’ rights. After all questions are answered to the satisfaction of the participant, and after he/she signs the consent document, then data collection will begin. Participants will be given a copy of the consent form(s).

All consent related documents (informational sheet and consent form) will be submitted with the protocol for review and approval by the IRB for the study. Subjects will be consented by the study project manager or research assistants in the ED waiting room or the ED treatment rooms. For remote recruitment of the study, consenting will occur over the phone. Potential subjects will review the consent form in detail with the person designated to consent (either PI or CRC) and have the ability to take the consent home for further review.

9.4.1 Alterations to Typical Consent Process

9.4.1.1 Waiver of Consent (In some cases for screening/portions of that study that qualify as minimal risk, a waiver of documentation of consent may be permissible IRB SOP)

Waiver or alteration of required elements of consent: According to HHS CFR 45.46.116(d): An IRB may approve a consent procedure which does not include, or which alters, some or all of the elements of informed consent, or waive the requirements to obtain informed consent. In order to qualify for a waiver or alteration to the informed consent process, please justify each of the following:

- the research involves no more than minimal risk to the subjects;
- the waiver or alteration will not adversely affect the rights and welfare of the subjects;
- the research could not practicably be carried out without the waiver or alteration; and

- whenever appropriate, the subjects will be provided with additional pertinent information after participation.

Full waiver of HIPAA authorization:

We request a full waiver of HIPAA authorization for the purposes of identifying potentially eligible families for remote recruitment into the study. A full waiver is required to identify visits to the ED in the 6-months prior to the current date.

Partial waiver of HIPAA authorization:

A partial waiver of HIPAA authorization for this study would allow the research study team to access and view the medical chart of children coming into the ED prior to approaching them for the study.

A partial waiver of HIPAA authorization is fundamental to the study's protocol because the research study team is able to streamline who is approached for the research study and best optimize staff time/resources. It also serves the function of protecting certain families from being approached for the study if they meet any of the study's exclusion criteria (e.g., coming into the ED for a critical injury or illness, etc.), if they have already been enrolled in the study or taken the screening survey in the past 6 months, or have declined to participate in the study.

Waiver of the requirement of obtaining a signed consent form:

We are requesting a waiver of the requirement of obtaining a signed consent form for part 1 of this study only. Part 1 of the study includes a brief (~5-10 minute) screening survey that collects one piece of identifying information (5-digit zip code) and presents no more than minimal risk of harm to participants. This screening survey serves the purpose of determining eligibility for part 2 of this study.

Waiver of informed consent:

We request waiver of informed consent for the retrospective review of visits to the Lurie Children's ED for the purposes of identifying potentially eligible families to screen for eligibility and recruit for participation in the study. The families identified for remote recruitment will be contacted via email, text message and/or phone for initial screening. The remote screening process (Part 1) will include the information sheet distributed via Qualtrics survey or reviewed verbally by the RC over the phone. Any caregiver potentially eligible for Part 2 of the study will complete a phone conversation with a Research Coordinator (RC). Informed consent for participation in part 2 of the study will be conducted verbally over the phone with electronic copies of the consent form delivered to potential participants via email and via a link to a Qualtrics survey. The Qualtrics survey will allow for the collection of electronic signatures from study participants.

Waiver of informed consent will be requested for review of the ED Electronic Medical Record (EMR)/ER Patient Tracking board to determine which families to approach for screening. Adult caregivers of children 6 months to 10 years of age, who bring their child to the LCH ED for any non-critical illness or injury, will be approached for eligibility screening. Again, the child age range was selected based on the American Academy of Pediatrics Guidelines for Child Passenger Safety.

10 Study Finances

10.1 Funding Source

This study is financed through a grant from the National Institute of Health (NIH), National Institute of Child Health and Human Development (NICHD).

10.2 Conflict of Interest

All study investigators will follow Lurie Children's Hospital of Chicago's Policy on Conflicts of Interest Related to Research.

10.3 Subject Stipends or Payments

Study-related costs:
The study team is paying for the following:

- Cost of text messages (both SMS and MMS) sent to a participant's phone from the study team while taking part in the study (12 months).
- Cost of new car seats, if provided to families.

The participant will be responsible for the following:

- Depending on their data plan, cost of text messages (both SMS and MMS) sent from their phone to the study team while taking part in the study (12 months).
- The cost of traveling to and from the 6 and 12-month follow-up visits (e.g., gas, public transportation).

The participant and/or their insurance company are responsible for the costs of normal care at LCH. Lurie Children's may be able to provide some financial help to patients. Ask their health team for more information about this program.

Study payments:
The participant will be paid up to $155 in total for taking part in all components of this study. They will only be paid for completed study visits. See the table below for information on the amounts they can earn for each of the tasks that they can finish while in the study.

Study Timeline	Task	Time to do task	Amount	Rate of payment	Type of payment	When payment is given	How payment is given
**Emergency room visit: $25**							
Baseline visit	Baseline survey	Within 5 business days	$20	1 time	gift card	After completion	via email
	Tiny Cargo check-in	Within 5 business days	$5	1 time	gift card	After completion	via email
**Monthly Photo Requests: $50**							
Photo request (8 over 12 months)	Monthly photo request	Within 4 hours	$5/each month	Up to 8 times	gift card	After photo set has been reviewed for quality and completeness	via email
	4 months of	4 months in	$5/every 4	Up to 2	gift card	After 4th photo	via email

**Study Timeline**	**Task**	**Time to do task**	**Amount**	**Rate of payment**	**Type of payment**	**When payment is given**	**How payment is given**
	photo requests	a row	months	times		set in a “streak” has been reviewed for quality and completeness	
**6 month follow-up visit: $35**							
6 month follow-up visit	Confirm 6 month visit	Prior to 6 month visit	$5	1 time	gift card	After 6 month follow-up visit	via email
	Survey & Tiny Cargo check-in	Within 1 month	$30 ($15 survey/ $10-15 TC check-in)	1 time	gift card	After 6 month follow-up visit	via email
**12 month follow-up visit: $45**							
12 month follow up visit	Confirm 12 month visit	Prior to 12 month visit	$5	1 time	gift card	After 12 month follow-up visit	via email
	Survey & Tiny Cargo check-in	Within 1 month	$40 ($25 survey/ $10-15 TC check-in)	1 time	gift card	After 12 month follow-up visit	via email

11 Publication Plan

Dr. Macy will ensure the intervention trial is registered at ClinicalTrials.gov, that the information entered into
ClincialTrials.gov is accurate, and that results from the trial will be submitted to ClincialTrials.gov at the completion of the study. The study will be registered at ClinicalTrials.gov prior to the recruitment of subjects, anticipated in October/November 2019. The results of the trial, including participant flow, demographic and baseline characteristics, primary and secondary outcomes, statistical tests, and adverse event information, will be uploaded within 1 year of the collection of 12-month outcomes from the final participant. Informed consent documents will reflect the plan to post the study information and results at ClinicalTrials.gov. Dr. Macy will adhere to Lurie Children’s Hospital policies for clinical trials registration and results reporting.

12 References

1. Macy ML, Cunningham RM, Resnicow K, Freed GL. Disparities in age-appropriate child passenger restraint use among children aged 1 to 12 years. *Pediatrics.* 2014;133(2):262-271.
2. Kiley K, Damian F, MacClaren W, et al. A computerized child passenger safety screening program in the emergency department. *Pediatric emergency care.* 2014;30(9):631-635.
3. Cease AT, King WD, Monroe KW. Analysis of child passenger safety restraint use at a pediatric emergency department. *Pediatric emergency care.* 2011;27(2):102-105.
4. Shields WC, McDonald EM, McKenzie L, Wang MC, Walker AR, Gielen AC. Using the pediatric emergency department to deliver tailored safety messages: results of a randomized controlled trial. *Pediatric emergency care.* 2013;29(5):628-634.
5. Johnston BD, Rivara FP, Droesch RM, Dunn C, Copass MK. Behavior change counseling in the emergency department to reduce injury risk: a randomized, controlled trial. *Pediatrics.* 2002;110(2 Pt 1):267-274.
6. Gielen AC, McKenzie LB, McDonald EM, et al. Using a computer kiosk to promote child safety: results of a randomized, controlled trial in an urban pediatric emergency department. *Pediatrics.* 2007;120(2):330-339.
7. Posner JC, Hawkins LA, Garcia-Espana F, Durbin DR. A randomized, clinical trial of a home safety intervention based in an emergency department setting. *Pediatrics.* 2004;113(6):1603-1608.
8. Melzer-Lange MD, Zonfrillo MR, Gittelman MA. Injury prevention: opportunities in the emergency department. *Pediatr Clin North Am.* 2013;60(5):1241-1253.
9. Gittelman MA, Pomerantz WJ, Laurence S. An emergency department intervention to increase booster seat use for lower socioeconomic families. *Acad Emerg Med.* 2006;13(4):396-400.
10. Zonfrillo MR, Mello MJ, Palmisciano LM. Usefulness of computerized pediatric motor vehicle safety discharge instructions. *Acad Emerg Med.* 2003;10(10):1131-1133.
11. Shenoi R, Saz EU, Jones JL, Ma L, Yusuf S. An emergency department intervention to improve knowledge of child passenger safety. *Pediatric emergency care.* 2010;26(12):881-887.
12. Schwebel DC, Tillman MA, Crew M, Muller M, Johnston A. Using interactive virtual presence to support accurate installation of child restraints: Efficacy and parental perceptions. *J Safety Res.* 2017;62:235-243.
13. Omaki E, Shields WC, McDonald E, et al. Evaluating a smartphone application to improve child passenger safety and fire safety knowledge and behaviour. *Inj Prev.* 2017;23(1):58.
14. Schwebel DC, Johnston A, Rouse J. Teaching infant car seat installation via interactive visual presence: An experimental trial. *Traffic Inj Prev.* 2017;18(2):188-192.
15. Gielen AC, McDonald EM, Omaki E, Shields W, Case J, Aitken M. A smartphone app to communicate child passenger safety: an application of theory to practice. *Health Educ Res.* 2015;30(5):683-692.
16. Fleisher L, Erkoboni D, Halkyard K, et al. Are mHealth Interventions to Improve Child Restraint System Installation of Value? A Mixed Methods Study of Parents. *Int J Environ Res Public Health.* 2017;14(10).
17. Centers for Disease Control and Prevention - Ten Leading Causes of Death and Injury, Highlighting Unintentional Injury Deaths, United States - 2014. http://www.cdc.gov/injury/images/lc-charts/leading_causes_of_injury_deaths_unintentional_injury_2014_1040w740h.gif. Accessed August 29, 2016.

18. National Center for Statistics and Analysis. Traffic Safety Facts. 2013 Data: Children. Report No. DOT HS 812 154. Washington, DC: National Highway Traffic Safety Administration; 2015.
19. Centers for Disease Control and Prevention - Ten Leading Causes of Death by Age Group, United States, 2013. http://www.cdc.gov/injury/wisqars/pdf/leading_causes_of_death_by_age_group_2013-a.pdf. Accessed August 29, 2016.
20. Insurance Institute for Highway Safety Highway Loss Data Institute Child safety Trends. 2016; http://www.iihs.org/iihs/topics/t/child-safety/fatalityfacts/child-safety. Accessed 10/12/2017.
21. National Center for Statistics and Analysis. Early Estimate of Motor Vehicle Traffice Fatalities for the First Nine Months (Jan-Sep) of 2015. (Crash*Stats Brief Statistical Summary. Report No. DOT HS 812 240). Washington, DC: National Highway Traffic Safety Administration; 2016.
22. *National Safety Council - Motor Vehicle Fatality Estimates.* Statistics Department, National Safety Council;2016.
23. Nonfatal Injury Reports, 2001 - 2014, Centers for Disease Control and Prevention, Web-based Injury Statistics Query and Reporting System (WISQARS). http://webappa.cdc.gov/sasweb/ncipc/nfirates2001.html. Accessed July 21, 2016, 2016.
24. Bergen G, Peterson C, Ederer D, et al. Vital signs: health burden and medical costs of nonfatal injuries to motor vehicle occupants - United States, 2012. *MMWR Morb Mortal Wkly Rep.* 2014;63(40):894-900.
25. Florence C, Haegerich T, Simon T, Zhou C, Luo F. Estimated Lifetime Medical and Work-Loss Costs of Emergency Department-Treated Nonfatal Injuries--United States, 2013. *MMWR Morb Mortal Wkly Rep.* 2015;64(38):1078-1082.
26. Kassam-Adams N, Bakker A, Marsac ML, Fein JA, Winston FK. Traumatic Stress, Depression, and Recovery: Child and Parent Responses After Emergency Medical Care for Unintentional Injury. *Pediatric emergency care.* 2015;31(11):737-742.
27. Winston FK, Puzino K, Romer D. Precision prevention: time to move beyond universal interventions. *Inj Prev.* 2016;22(2):87-91.
28. Du W, Finch CF, Hayen A, Bilston L, Brown J, Hatfield J. Relative benefits of population-level interventions targeting restraint-use in child car passengers. *Pediatrics.* 2010;125(2):304-312.
29. Bae JY, Anderson E, Silver D, Macinko J. Child passenger safety laws in the United States, 1978-2010: policy diffusion in the absence of strong federal intervention. *Soc Sci Med.* 2014;100:30-37.
30. Brown J, Keay L, Hunter K, Bilston LE, Simpson JM, Ivers R. Increase in best practice child car restraint use for children aged 2-5 years in low socioeconomic areas after introduction of mandatory child restraint laws. *Aust N Z J Public Health.* 2013;37(3):272-277.
31. Jones LE, Ziebarth NR. U.S. Child Safety Seat Laws: Are they Effective, and Who Complies? *J Policy Anal Manage.* 2017;36(3):584-607.
32. Durbin DR. Child Passenger Safety - Policy Statement. *Pediatrics.* 2011;127(4):788-793.
33. IIHS. Insurance Institute for Highway Safety - Child Safety: State laws. 2017; http://www.iihs.org/iihs/topics/laws/safetybeltuse?topicName=child-safety. Accessed 3/1/2017.
34. Chang BA, Ebel BE, Rivara FP. Child passenger safety: potential impact of the Washington State booster seat law on childcare centers. *Inj Prev.* 2002;8(4):284-288.
35. Bryant-Stephens T, Garcia-Espana JF, Winston FK. Boosting restraint norms: a community-delivered campaign to promote booster seat use. *Traffic Inj Prev.* 2013;14(6):578-583.

36. Pickrell TM, Ye T. *The 2009 National Survey of the Use of Booster Seats.* Washington, DC: National Center for Statistics and Analysis, National Highway Traffic Safety Administration; September 2010.
37. Pickrell TM, Choi E-H. *The 2013 National Survey of the Use of Booster Seats.* Washington, DC: National Highway Traffic Safety Administration; June 2014.
38. Li HR, Pickrell TM, KC S. *The 2015 National Survey of the Use of Booster Seats.* Washington, DC: National Highway Traffic Safety Administration; June 2016.
39. Dodington J, Violano P, Baum CR, Bechtel K. Drugs, guns and cars: how far we have come to improve safety in the United States; yet we still have far to go. *Pediatr Res.* 2017;81(1-2):227-232.
40. Pickrell TM, Ye T. *The 2011 National Survey of the Use of Booster Seats.* Washington, DC: National Center for Statistics and Analysis, National Highway Traffic Safety Administration; September 2013.
41. Sauber-Schatz EK, West BA, Bergen G. Vital signs: restraint use and motor vehicle occupant death rates among children aged 0-12 years - United States, 2002-2011. *MMWR Morb Mortal Wkly Rep.* 2014;63(5):113-118.
42. Aitken ME, Miller BK, Anderson BL, et al. Promoting use of booster seats in rural areas through community sports programs. *J Rural Health.* 2013;29 Suppl 1:s70-78.
43. Winston FK, Erkoboni D, Xie D. Identifying interventions that promote belt-positioning booster seat use for parents with low educational attainment. *J Trauma.* 2007;63(3 Suppl):S29-38.
44. Aitken ME, Mullins SH, Lancaster VE, Miller BK. "Cubs Click It For Safety": a school-based intervention for Tween passenger safety. *J Trauma.* 2007;63(3 Suppl):S39-43.
45. Toward Zero Deaths - National Strategy on Highway Safety. 2016; http://www.towardzerodeaths.org/. Accessed August 29, 2016.
46. Centers for Disease Control and Prevention - Winnable Battles - Motor Vehicle Injuries. 2016; https://www.cdc.gov/winnablebattles/motorvehicleinjury/index.html. Accessed July 28, 2016.
47. Healthy People 2020 Topics & Objectives: Injury and Violence Prevention: IVP-16: Increase age-appropriate vehcile restraint system use in children aged 1 to 3 years, 4 to 7 years, and 8 to 12 years. 2010; http://www.healthypeople.gov/2020/topicsobjectives2020/objectiveslist.aspx?topicid=24. Accessed 7/21/2016.
48. National Center for Statistics and Analysis. Traffic Safety Facts. 2009 Data: Children. Report No. DOT HS 811 387. Washington, DC: National Highway Traffic Safety Administration; 2010.
49. Morse AM, Aitken ME, Mullins SH, et al. Child seat belt guidelines: Examining the 4 feet 9 inches rule as the standard. *J Trauma Acute Care Surg.* 2017;83(2):305-309.
50. Huang S, Reed MP. *Comparison of Child Body Dimensions with Rear Seat Geometry.* Society of Automotive Engineers Technical Paper Series;2006.
51. Bilston LE, Sagar N. Geometry of rear seats and child restraints compared to child anthropometry. *Stapp Car Crash J.* 2007;51:275-298.
52. Reed MP, Ebert-Hamilton SM, Manary MA, Klinich KD, Schneider LW. Improved positioning procedures for 6YO and 10YO ATDs based on child occupant postures. *Stapp Car Crash J.* 2006;50:337-388.
53. Reed MP, Ebert SM, Sherwood CP, Klinich KD, Manary MA. Evaluation of the static belt fit provided by belt-positioning booster seats. *Accid Anal Prev.* 2009;41(3):598-607.
54. Hu J, Klinich KD, Reed MP, Kokkolaras M, Rupp JD. Development and validation of a modified Hybrid-III six-year-old dummy model for simulating submarining in motor-vehicle crashes. *Med Eng Phys.* 2012;34(5):541-551.

55. Hu J, Wu J, Reed MP, Klinich KD, Cao L. Rear seat restraint system optimization for older children in frontal crashes. *Traffic Inj Prev.* 2013;14(6):614-622.
56. Macy ML, Freed GL, Reed MP. Child passenger restraints in relation to other second-row passengers: an analysis of the 2007-2009 National Survey of the Use of Booster Seats. *Traffic Inj Prev.* 2013;14(2):209-214.
57. Klinich KD, Manary MA, Flannagan CA, et al. Effects of child restraint system features on installation errors. *Appl Ergon.* 2014;45(2):270-277.
58. Hu J, Manary MA, Klinich KD, Reed MP. Evaluation of ISO CRS Envelopes Relative to U.S. Vehicles and Child Restraint Systems. *Traffic Inj Prev.* 2015;16(8):781-785.
59. Reed MP, Klinich KD. Predicting vehicle belt fit for children ages 6-12. *Traffic Inj Prev.* 2016;17(1):58-64.
60. Macy ML, Reed MP, Freed GL. Driver report of improper seat belt position among 4- to 9-year-old children. *Acad Pediatr.* 2011;11(6):487-492.
61. Durbin DR. Child Passenger Safety - Technical Report. *Pediatrics.* 2011;127(4):e1050-1066.
62. Henary B, Sherwood CP, Crandall JR, et al. Car safety seats for children: rear facing for best protection. *Inj Prev.* 2007;13(6):398-402.
63. Durbin DR, Elliott MR, Winston FK. Belt-positioning booster seats and reduction in risk of injury among children in vehicle crashes. *Jama.* 2003;289(21):2835-2840.
64. Arbogast KB, Jermakian JS, Kallan MJ, Durbin DR. Effectiveness of belt positioning booster seats: an updated assessment. *Pediatrics.* 2009;124(5):1281-1286.
65. Macy ML, Freed GL. Child Passenger Safety Practices in the U.S.: Dispartities in Light of Updated Recommendations. *American Journal of Preventive Medicine.* 2012;43(3).
66. Wier LM, Yu H, Owens PL, Washington R. Overview of Children in the Emergency Department, 2010: Statistical Brief #157. 2006.
67. Tang N, Stein J, Hsia RY, Maselli JH, Gonzales R. Trends and characteristics of US emergency department visits, 1997-2007. *Jama.* 2010;304(6):664-670.
68. *National Center for Health Statistics. Health, United States, 2015: With Special Feature on Racial and Ethnic Health Disparities. Hyattsville, Maryland. .* 2016.
69. Merrill C, Owens P, Stocks C. Pediatric Emergency Department Visits in Community Hospitals from Selected States, 2005. *AHRQ HCUP Statistical Brief.* 2008;52.
70. Macy ML, Zonfrillo MR, Cook LJ, et al. Patient- and Community-Level Sociodemographic Characteristics Associated with Emergency Department Visits for Childhood Injury. *The Journal of pediatrics.* 2015;167(3):711-718 e711-714.
71. Jaeger MW, Ambadwar PB, King AJ, Onukwube JI, Robbins JM. Emergency Care of Children with Ambulatory Care Sensitive Conditions in the United States. *J Emerg Med.* 2015;49(5):729-739.
72. Fernandez WG, Mitchell PM, Jamanka AS, et al. Brief motivational intervention to increase self-reported safety belt use among emergency department patients. *Acad Emerg Med.* 2008;15(5):419-425.
73. Buchbinder M, Wilbur R, Zuskov D, McLean S, Sleath B. Teachable moments and missed opportunities for smoking cessation counseling in a hospital emergency department: a mixed-methods study of patient-provider communication. *BMC Health Serv Res.* 2014;14:651.
74. Ersel M, Kitapcioglu G, Solak ZA, Yuruktumen A, Karahalli E, Cevrim O. Are emergency department visits really a teachable moment? Smoking cessation promotion in emergency department. *Eur J Emerg Med.* 2010;17(2):73-79.
75. Vokes NI, Bailey JM, Rhodes KV. "Should I give you my smoking lecture now or later?" Characterizing emergency physician smoking discussions and cessation counseling. *Ann Emerg Med.* 2006;48(4):406-414, 414 e401-407.

76. Williams S, Brown A, Patton R, Crawford MJ, Touquet R. The half-life of the 'teachable moment' for alcohol misusing patients in the emergency department. *Drug Alcohol Depend.* 2005;77(2):205-208.
77. Blow FC, Ilgen MA, Walton MA, et al. Severity of baseline alcohol use as a moderator of brief interventions in the emergency department. *Alcohol Alcohol.* 2009;44(5):486-490.
78. Bohnert AS, Bonar EE, Cunningham R, et al. A pilot randomized clinical trial of an intervention to reduce overdose risk behaviors among emergency department patients at risk for prescription opioid overdose. *Drug Alcohol Depend.* 2016;163:40-47.
79. Cunningham R, Knox L, Fein J, et al. Before and after the trauma bay: the prevention of violent injury among youth. *Ann Emerg Med.* 2009;53(4):490-500.
80. Cunningham RM, Resko SM, Harrison SR, et al. Screening adolescents in the emergency department for weapon carriage. *Acad Emerg Med.* 2010;17(2):168-176.
81. Cunningham RM, Walton MA, Roahen Harrison S, et al. Past-year intentional and unintentional injury among teens treated in an inner-city emergency department. *J Emerg Med.* 2011;41(4):418-426.
82. Bourgeois FT, Shannon MW. Emergency care for children in pediatric and general emergency departments. *Pediatric emergency care.* 2007;23(2):94-102.
83. Macy ML, Carter PM, Bingham CR, Cunningham RM, Freed GL. Potential distractions and unsafe driving behaviors among drivers of 1- to 12-year-old children. *Acad Pediatr.* 2014;14(3):279-286.
84. Macy ML, Clark SJ, Freed GL, et al. Carpooling and booster seats: a national survey of parents. *Pediatrics.* 2012;129(2):290-298.
85. Macy ML, Butchart AT, Singer DC, Gebremariam A, Clark SJ, Davis MM. Looking Back on Rear-Facing Car Seats: Surveying US Parents in 2011 and 2013. *Acad Pediatr.* 2015;15(5):526-533.
86. Kroeker AM, Teddy AJ, Macy ML. Car seat inspection among children older than 3 years: Using data to drive practice in child passenger safety. *J Trauma Acute Care Surg.* 2015;79(3 Suppl 1):S48-54.
87. Macy M, Clark S, Freed G. Emergency Physician Perspectives on Child Passenger Safety. Annual Meeting of The Society for Academic Emergency Medicine; 2011; Boston.
88. Macy ML, Clark SJ, Cunningham RM, Freed GL. Availability of child passenger safety resources to emergency physicians practicing in emergency departments within pediatric, adult, and nontrauma centers: a national survey. *Pediatric emergency care.* 2013;29(3):324-330.
89. Resnicow K, Jackson A, Blissett D, et al. Results of the healthy body healthy spirit trial. *Health Psychol.* 2005;24(4):339-348.
90. Resnicow K, Wallace DC, Jackson A, et al. Dietary change through African American churches: baseline results and program description of the eat for life trial. *J Cancer Educ.* 2000;15(3):156-163.
91. Resnicow K, Yaroch AL, Davis A, et al. GO GIRLS!: Development of a Community-Based Nutrition and Physical Activity Program for Overweight African-American Adolescent Females. *Journal of nutrition education.* 1999;31(5):287-289.
92. Holstad M, DiIorio C, Kelley M, Resnicow K, Sharma S. Group Motivational Interviewing to Promote Adherence to Antiretroviral Medications and Risk Reduction Behaviors in HIV Infected Women. *AIDS and behavior.* 2011;15(5):885-896.
93. Resnicow K, DiIorio C, Soet JE, Ernst D, Borrelli B, Hecht J. Motivational interviewing in health promotion: it sounds like something is changing. *Health Psychol.* 2002;21(5):444-451.

94. Resnicow K, Jackson A, Wang T, et al. A motivational interviewing intervention to increase fruit and vegetable intake through Black churches: results of the Eat for Life trial. *Am J Public Health.* 2001;91(10):1686-1693.

95. Resnicow K, Yaroch AL, Davis A, et al. GO GIRLS!: results from a nutrition and physical activity program for low-income, overweight African American adolescent females. *Health Educ Behav.* 2000;27(5):616-631.

96. Campbell MK, Hudson MA, Resnicow K, Blakeney N, Paxton A, Baskin M. Church-based health promotion interventions: evidence and lessons learned. *Annu Rev Public Health.* 2007;28:213-234.

97. Fuemmeler BF, Masse LC, Yaroch AL, et al. Psychosocial mediation of fruit and vegetable consumption in the body and soul effectiveness trial. *Health Psychol.* 2006;25(4):474-483.

98. Resnicow K, Campbell M, Carr C, et al. Body and soul. A dietary intervention conducted through African-American churches. *Am J Prev Med.* 2004;27(2):97-105.

99. Hawkins RP, Kreuter M, Resnicow K, Fishbein M, Dijkstra A. Understanding tailoring in communicating about health. *Health Educ Res.* 2008;23(3):454-466.

100. Resnicow K, Davis RE, Zhang G, et al. Tailoring a fruit and vegetable intervention on novel motivational constructs: results of a randomized study. *Ann Behav Med.* 2008;35(2):159-169.

101. An LC, Perry CL, Lein EB, et al. Strategies for increasing adherence to an online smoking cessation intervention for college students. *Nicotine Tob Res.* 2006;8 Suppl 1:S7-12.

102. An LC, Zhu SH, Nelson DB, et al. Benefits of telephone care over primary care for smoking cessation: a randomized trial. *Archives of internal medicine.* 2006;166(5):536-542.

103. An LC, Klatt C, Perry CL, et al. The RealU online cessation intervention for college smokers: a randomized controlled trial. *Prev Med.* 2008;47(2):194-199.

104. An LC, Schillo BA, Saul JE, et al. Utilization of smoking cessation informational, interactive, and online community resources as predictors of abstinence: cohort study. *J Med Internet Res.* 2008;10(5):e55.

105. Zulman DM, Schafenacker A, Barr KL, et al. Adapting an in-person patient-caregiver communication intervention to a tailored web-based format. *Psychooncology.* 2012;21(3):336-341.

106. Gatwood J, Balkrishnan R, Erickson SR, An LC, Piette JD, Farris KB. Addressing medication nonadherence by mobile phone: development and delivery of tailored messages. *Res Social Adm Pharm.* 2014;10(6):809-823.

107. An LC, Wallner L, Kirch MA. Online Social Engagement by Cancer Patients: A Clinic-Based Patient Survey. *JMIR cancer.* 2016;2(2):e10.

108. Gatwood J, Balkrishnan R, Erickson SR, An LC, Piette JD, Farris KB. The impact of tailored text messages on health beliefs and medication adherence in adults with diabetes: A randomized pilot study. *Res Social Adm Pharm.* 2016;12(1):130-140.

109. Maher M, Kaziunas E, Ackerman M, et al. User-Centered Design Groups to Engage Patients and Caregivers with a Personalized Health Information Technology Tool. *Biol Blood Marrow Transplant.* 2016;22(2):349-358.

110. McClure JB, Anderson ML, Bradley K, An LC, Catz SL. Evaluating an Adaptive and Interactive mHealth Smoking Cessation and Medication Adherence Program: A Randomized Pilot Feasibility Study. *JMIR mHealth and uHealth.* 2016;4(3):e94.

111. Macy ML, Davis MM, Clark SJ, Stanley RM. Parental health literacy and asthma education delivery during a visit to a community-based pediatric emergency department: a pilot study. *Pediatric emergency care.* 2011;27(6):469-474.

112. Walton MA, Chermack ST, Shope JT, et al. Effects of a brief intervention for reducing violence and alcohol misuse among adolescents: a randomized controlled trial. *Jama.* 2010;304(5):527-535.
113. Carter PM, Walton MA, Zimmerman MA, Chermack ST, Roche JS, Cunningham RM. Efficacy of a Universal Brief Intervention for Violence Among Urban Emergency Department Youth. *Acad Emerg Med.* 2016.
114. Carter PM, Walton MA, Roehler DR, et al. Firearm violence among high-risk emergency department youth after an assault injury. *Pediatrics.* 2015;135(5):805-815.
115. Cunningham RM, Carter PM, Ranney M, et al. Violent reinjury and mortality among youth seeking emergency department care for assault-related injury: a 2-year prospective cohort study. *JAMA Pediatr.* 2015;169(1):63-70.
116. Cunningham RM, Chermack ST, Ehrlich PF, et al. Alcohol Interventions Among Underage Drinkers in the ED: A Randomized Controlled Trial. *Pediatrics.* 2015;136(4):e783-793.
117. Ranney ML, Goldstick J, Eisman A, Carter PM, Walton M, Cunningham RM. Effects of a brief ED-based alcohol and violence intervention on depressive symptoms. *Gen Hosp Psychiatry.* 2017;46:44-48.
118. Manary MA, Reed MP, Klinich KD, Ritchie NL, Schneider LW. The effects of tethering rear -facing child restraint systems on ATD responses. *Annu Proc Assoc Adv Automot Med.* 2006;50:397-410.
119. Reed MA, Naftel RP, Carter S, MacLennan PA, McGwin G, Jr., Rue LW, 3rd. Motor vehicle restraint system use and risk of spine injury. *Traffic Inj Prev.* 2006;7(3):256-263.
120. Rupp JD, Flannagan CA, Leslie AJ, Hoff CN, Reed MP, Cunningham RM. Effects of BMI on the risk and frequency of AIS 3+ injuries in motor-vehicle crashes. *Obesity (Silver Spring).* 2013;21(1):E88-97.
121. Carter PM, Flannagan CA, Reed MP, Cunningham RM, Rupp JD. Comparing the effects of age, BMI and gender on severe injury (AIS 3+) in motor-vehicle crashes. *Accid Anal Prev.* 2014;72:146-160.
122. Park BD, Ebert S, Reed MP. A Parametric Model of Child Body Shape in Seated Postures. *Traffic Inj Prev.* 2016:0.
123. Zaseck LW, Orton NR, Gruber R, et al. The influence of personal protection equipment, occupant body size, and restraint system on the frontal impact responses of Hybrid III ATDs in tactical vehicles. *Traffic Inj Prev.* 2017:0.
124. Prosser LA, Payne K, Rusinak D, Shi P, Uyeki T, Messonnier M. Valuing health across the lifespan: health state preferences for seasonal influenza illnesses in patients of different ages. *Value Health.* 2011;14(1):135-143.
125. Prosser LA, Kong CY, Rusinak D, Waisbren SL. Projected costs, risks, and benefits of expanded newborn screening for MCADD. *Pediatrics.* 2010;125(2):e286-294.
126. O'Brien MA, Prosser LA, Paradise JL, et al. New vaccines against otitis media: projected benefits and cost-effectiveness. *Pediatrics.* 2009;123(6):1452-1463.
127. Prosser LA, O'Brien MA, Molinari NA, et al. Non-traditional settings for influenza vaccination of adults: costs and cost effectiveness. *Pharmacoeconomics.* 2008;26(2):163-178.
128. Prosser LA, Bridges CB, Uyeki TM, et al. Health benefits, risks, and cost-effectiveness of influenza vaccination of children. *Emerg Infect Dis.* 2006;12(10):1548-1558.
129. Prosser LA, Bridges CB, Uyeki TM, et al. Values for preventing influenza-related morbidity and vaccine adverse events in children. *Health Qual Life Outcomes.* 2005;3:18.
130. Prosser LA, Ray GT, O'Brien M, Kleinman K, Santoli J, Lieu TA. Preferences and willingness to pay for health states prevented by pneumococcal conjugate vaccine. *Pediatrics.* 2004;113(2):283-290.

131. Johnston BD, Bennett E, Quan L, Gonzalez-Walker D, Crispin B, Ebel B. Factors influencing booster seat use in a multiethnic community: lessons for program implementation. *Health Promot Pract.* 2009;10(3):411-418.

132. Vansteenkiste M, Williams GC, Resnicow K. Toward systematic integration between self-determination theory and motivational interviewing as examples of top-down and bottom-up intervention development: autonomy or volition as a fundamental theoretical principle. *Int J Behav Nutr Phys Act.* 2012;9:23.

133. Gittelman MA, Pomerantz WJ, McClanahan N, Damon A, Ho M. A computerized kiosk to teach injury prevention: is it as effective as human interaction? *J Trauma Acute Care Surg.* 2014;77(3 Suppl 1):S2-7.

134. Brixey S, Ravindran K, Guse CE. Legislating child restraint usage -Its effect on self-reported child restraint use rates in a central city. *J Safety Res.* 2010;41(1):47-52.

135. Rudin-Brown CM, Kramer C, Langerak R, Scipione A, Kelsey S. Standardized error severity score (ESS) ratings to quantify risk associated with child restraint system (CRS) and booster seat misuse. *Traffic Inj Prev.* 2017;18(8):870-876.

136. Rich B, Moodie EE, Stephens DA. Simulating sequential multiple assignment randomized trials to generate optimal personalized warfarin dosing strategies. *Clinical trials (London, England).* 2014;11(4):435-444.

137. Lei H, Nahum-Shani I, Lynch K, Oslin D, Murphy SA. A "SMART" design for building individualized treatment sequences. *Annu Rev Clin Psychol.* 2012;8:21-48.

138. Chronis-Tuscano A, Wang CH, Strickland J, Almirall D, Stein MA. Personalized Treatment of Mothers With ADHD and Their Young At-Risk Children: A SMART Pilot. *J Clin Child Adolesc Psychol.* 2016;45(4):510-521.

139. Connell AM, Dishion TJ. Reducing depression among at-risk early adolescents: three-year effects of a family-centered intervention embedded within schools. *J Fam Psychol.* 2008;22(4):574-585.

140. Hartlieb KB, Jacques-Tiura AJ, Naar-King S, Ellis DA, Jen KL, Marshall S. Recruitment strategies and the retention of obese urban racial/ethnic minority adolescents in clinical trials: the FIT families project, Michigan, 2010-2014. *Prev Chronic Dis.* 2015;12:E22.

141. Po'e EK, Heerman WJ, Mistry RS, Barkin SL. Growing Right Onto Wellness (GROW): a family-centered, community-based obesity prevention randomized controlled trial for preschool child-parent pairs. *Contemp Clin Trials.* 2013;36(2):436-449.

142. Vansteenkiste M, Sheldon KM. There's nothing more practical than a good theory: Integrating motivational interviewing and self-determination theory. *British Journal of Clinical Psychology.* 2006;45:63-82.

143. Markland D, Ryan RM, Tobin VJ, Rollnick S. Motivational interviewing and self-determination theory. *J Soc Clin Psychol.* 2005;24(6):811-831.

144. Resnicow K, McMaster F, Rollnick S. Action reflections: a client-centered technique to bridge the WHY-HOW transition in Motivational Interviewing. *Behav Cogn Psychother.* 2012;40(4):474-480.

145. Schwartz RP, Hamre R, Dietz WH, et al. Office-based motivational interviewing to prevent childhood obesity: a feasibility study. *Archives of pediatrics & adolescent medicine.* 2007;161(5):495-501.

146. Resnicow K, Davis RE, Rollnick S. Motivational Interviewing for Pediatric Obesity: Conceptual Issues and Evidence Review. In: Birch L, Dietz WH, eds. *Eating Behaviors of the Young Child: Prenatal and Postnatal Influences for Healthy Eating*. Elk Grove Village: American Academy of Pediatrics; 2008.

147. Resnicow K, Baskin M, Rahotep S, Periasamy S, Rollnick S. Motivational Interviewing in Health Promotion & Behavioral Medicine Settings. In: Cox W, Klinger E, eds. *Handbook of Motivational Counseling: Motivating People for Change*. Sussex, UK: John Wiley and Sons; 2003:457-479.

148. Moyers TB, Rowell LN, Manuel JK, Ernst D, Houck JM. The Motivational Interviewing Treatment Integrity Code (MITI 4): Rationale, Preliminary Reliability and Validity. *J Subst Abuse Treat.* 2016;65:36-42.
149. Dijkstra A. Working mechanisms of computer-tailored health education: evidence from smoking cessation. *Health Educ Res.* 2005;20(5):527-539.
150. Fisher TL, Burnet DL, Huang ES, Chin MH, Cagney KA. Cultural leverage: interventions using culture to narrow racial disparities in health care. *Med Care Res Rev.* 2007;64(5 Suppl):243S-282S.
151. AAP. Car Safety Seats: Product Listing for 2014, American Academy of Pediatrics, Healthy Children. 2014; http://www.healthychildren.org/English/safety-prevention/on-the-go/pages/Car-Safety-Seats-Product-Listing.aspx. Accessed November 9, 2014.
152. United States Census Bureau *American Community Survey*. ; http://www.census.gov/programs-surveys/acs/data.html. Accessed 5/12/2015.
153. Cunningham R, Walton MA, Weber JE, et al. One-year medical outcomes and emergency department recidivism after emergency department observation for cocaine-associated chest pain. *Ann Emerg Med.* 2009;53(3):310-320.
154. BootsMiller BJ, Ribisl KM, Mowbray CT, Davidson WS, Walton MA, Herman SE. Methods of ensuring high follow-up rates: lessons from a longitudinal study of dual diagnosed participants. *Subst Use Misuse.* 1998;33(13):2665-2685.
155. Cunningham RM, Chermack ST, Zimmerman MA, et al. Brief motivational interviewing intervention for peer violence and alcohol use in teens: one-year follow-up. *Pediatrics.* 2012;129(6):1083-1090.
156. Cunningham RM, Walton MA, Goldstein A, et al. Three-month follow-up of brief computerized and therapist interventions for alcohol and violence among teens. *Acad Emerg Med.* 2009;16(11):1193-1207.
157. Outman R, Cunningham RM, Walton MA, Booth BM. Enhancing follow-up rates in longitudinal research: Alcohol and cocaine users in an inner-city ER study. *Alcoholism-Clinical and Experimental Research.* 2007;31(6):172a-172a.
158. Ryan RM, Connell JP. Perceived locus of causality and internalization: examining reasons for acting in two domains. *J Pers Soc Psychol.* 1989;57(5):749-761.
159. Williams GC, Grow VM, Freedman ZR, Ryan RM, Deci EL. Motivational predictors of weight loss and weight-loss maintenance. *J Pers Soc Psychol.* 1996;70(1):115-126.
160. Williams GC, Rodin GC, Ryan RM, Grolnick WS, Deci EL. Autonomous regulation and long-term medication adherence in adult outpatients. *Health Psychol.* 1998;17(3):269-276.
161. Williams GC, Freedman ZR, Deci EL. Supporting autonomy to motivate patients with diabetes for glucose control. *Diabetes Care.* 1998;21(10):1644-1651.
162. Williams GC, Cox EM, Kouides R, Deci EL. Presenting the facts about smoking to adolescents: effects of an autonomy-supportive style. *Archives of pediatrics & adolescent medicine.* 1999;153(9):959-964.
163. Osborn CY, Weiss BD, Davis TC, et al. Measuring adult literacy in health care: Performance of the newest vital Sign. *American Journal of Health Behavior.* 2007;31:S36-S46.
164. Morrison AK, Schapira MM, Hoffmann RG, Brousseau DC. Measuring Health Literacy in Caregivers of Children: A Comparison of the Newest Vital Sign and S-TOFHLA. *Clinical Pediatrics.* 2014;53(13):1264-1270.
165. Spielberger CD. *Manual for the State-Trait Anxiety Inventory.* Palo Alto, CA: Consulting Psychologists Press; 1983.
166. Witte K, Cameron KA, McKeon JK, Berkowitz JM. Predicting risk behaviors: development and validation of a diagnostic scale. *J Health Commun.* 1996;1(4):317-341.

167. Carver CS. You want to measure coping but your protocol's too long: consider the brief COPE. *Int J Behav Med.* 1997;4(1):92-100.
168. Crowne DP, Marlowe D. A new scale of social desirability independent of psychopathology. *J Consult Psychol.* 1960;24:349-354.
169. Agran PF, Anderson C, Winn DG. Development of a child safety seat Hassles Scale in a largely low-income Latino population. *Pediatrics.* 2006;118(1):e85-91.
170. Gelman A, Hill J. *Data Analysis Using Regression and Multilevel/Hierarchical Models.* Cambridge Univesrity Press; 2007.
171. Vickers AJ, Altman DG. Statistics notes: Analysing controlled trials with baseline and follow up measurements. *BMJ (Clinical research ed.* 2001;323(7321):1123-1124.
172. Twisk JW, de Vente W. The analysis of randomised controlled trial data with more than one follow-up measurement. A comparison between different approaches. *Eur J Epidemiol.* 2008;23(10):655-660.
173. Imai K, Keele L, Tingley D. A General Approach to Causal Mediation Analysis. *Psychol Methods.* 2010;15(4):309-334.
174. Baron RM, Kenny DA. The Moderator Mediator Variable Distinction in Social Psychological-Research - Conceptual, Strategic, and Statistical Considerations. *J Pers Soc Psychol.* 1986;51(6):1173-1182.
175. Richiardi L, Bellocco R, Zugna D. Mediation analysis in epidemiology: methods, interpretation and bias. *IntJEpidemiol.* 2013;42(5):1511-1519.
176. Tingley D, Yamamoto T, Hirose K, Keele L, Imai K. mediation: R Package for Causal Mediation Analysis. *J Stat Softw.* 2014;59(5).
177. Muthen B, Shedden K. Finite mixture modeling with mixture outcomes using the EM algorithm. *Biometrics.* 1999;55(2):463-469.
178. Nagin DS, Tremblay RE. Analyzing developmental trajectories of distinct but related behaviors: a group-based method. *Psychol Methods.* 2001;6(1):18-34.
179. Shedden K, Zucker RA. Regularized finite mixture models for probability trajectories. *Psychometrika.* 2008;73(4):625-646.
180. Verbeke G, Lesaffre E. A linear mixed-effects model with heterogeneity in the random-effects population. . *J Am Stat Assoc.* 1996;91(433):217-221.
181. Nagin D. *Group-based modeling of development.* Harvard University Press; 2005.
182. Nagin DS. Analyzing developmental trajectories: a semiparametric, group-based approach. *Psychol Methods.* 1999;4(2):139.
183. Wood S. *Generalized additive models: an introduction with R.* CRC Press; 2006.
184. Grun B, Leisch F. FlexMix version 2: finite mixtures with concomitant variables and varying and constant parameters. 2008.
185. Nyland KL, Asparouhav T, Muthen BO. Deciding on the number of classes in latent class analysis and growth mixture modeling: A Monte Carlo simulation study. *Structural Equation Modeling.* 2007;14(4):535-569.
186. Walton MA, Epstein-Ngo Q, Carter PM, et al. Marijuana use trajectories among drug-using youth presenting to an urban emergency department: Violence and social influences. *Drug Alcohol Depend.* 2017;173:117-125.
187. Anderson RE, Bonar EE, Walton MA, et al. A Latent Profile Analysis of Aggression and Victimization Across Relationship Types Among Veterans Who Use Substances. *J Stud Alcohol Drugs.* 2017;78(4):597-607.
188. Agrawal A, Lynskey MT, Madden PA, Bucholz KK, Heath AC. A latent class analysis of illicit drug abuse/dependence: results from the National Epidemiological Survey on Alcohol and Related Conditions. *Addiction.* 2007;102(1):94-104.
189. Cohen J. *Statistical Power Analysis for the Behavioral Sciences.* 2nd ed1988.
190. Fan X. Power of latent growth modeling for detecting group differences in linear growth trajectory parameters. *Structural Equation Modeling.* 2003;10(3):380-400.

191. Clarifying NIH Priorities for Health Economics Research. In: NIH, ed2015.

192. Drummond MF, Schulpher MJ, Claxton K, Stoddart GL, Torrance GW. *Methods for the economic evaluation of health care programmes.*: Oxford University Press; 2015.

193. Neumann PJ, Sanders GD, Russell LB, Siegel JE, Ganiats TG. *Cost-effectiveness in health and medicine.* Oxford University Press; 2016.

194. Glick HA, Doshi JA, Sonnad SS, Polsky D. *Economic evaluation in clinical trials.* Oxford University Press; 2014.

195. Yarnall KS, Pollak KI, Ostbye T, Krause KM, Michener JL. Primary care: is there enough time for prevention? *Am J Public Health.* 2003;93(4):635-641.

196. Bush JW, Chen MM, Patrick DL, Tserg RL. Health status index in cost-effectiveness: Analysis of PKU program. *Health Study Indexes*. Chicago: Hospital Research and Educational Trust; 1973:172-209.

197. Drummond MF, Drummond MFMfteeohcp. *Methods for the economic evaluation of health care programmes.* 3rd ed. Oxford ; New York: Oxford University Press; 2005.

198. Cunningham RM, Vaidya RS, Walton M, Maio RF. Training emergency medicine nurses and physicians in youth violence prevention. *Am J Prev Med.* 2005;29(5 Suppl 2):220-225.

199. Goldstein AL, Walton MA, Cunningham RM, Trowbridge MJ, Maio RF. Violence and substance use as risk factors for depressive symptoms among adolescents in an urban emergency department. *J Adolesc Health.* 2007;40(3):276-279.

200. Walton MA, Goldstein AL, Chermack ST, et al. Brief alcohol intervention in the emergency department: moderators of effectiveness. *J Stud Alcohol Drugs.* 2008;69(4):550-560.

201. Booth BM, Weber JE, Walton MA, et al. Characteristics of cocaine users presenting to an emergency department chest pain observation unit. *Acad Emerg Med.* 2005;12(4):329-337.

202. Resko SM, Walton MA, Chermack ST, Blow FC, Cunningham RM. Therapist competence and treatment adherence for a brief intervention addressing alcohol and violence among adolescents. *J Subst Abuse Treat.* 2012;42(4):429-437.
