# Supplementary material for: Multimodal Intervention and Child Passenger Safety Guideline Adherence in Young Children: A Sequential, Multiple-Assignment, Randomized Clinical Trial
Source: JAMA Netw Open. 2025 Sep 29;8(9):e2533912. doi: 10.1001/jamanetworkopen.2025.33912 (PMC12481228; doi:10.1001/jamanetworkopen.2025.33912)
Supplement: Supplement 2. — eMethods. eTable 1. Child Passenger Restraint System Age and Size Criteria for Dichotomous Outcome Determination eTable 2. Counts of Child Passenger Restraint Systems by Lower Weight Limit eTable 3. Counts of Child Passenger Restraint Systems by Upper Weight Limit eTable 4. Counts of Child Passenger Restraint Systems by Lower Height Limit eTable 5. Counts of Child Passenger Restraint Systems by Upper Height Limit eTable 6. Phase I: Unadjusted and Adjusted Odds of Child Passenger Safety Guideline Adherence at 6-Month Follow-Up eTable 7. Phase II: Unadjusted and Adjusted Odds of Child Passenger Safety Guideline Adherence at 12-Month Follow-Up eTable 8. Maintenance Effects: Unadjusted and Adjusted Odds of Child Passenger Safety Guideline Adherence at 12-Month Follow-Up eTable 9. Unadjusted and Adjusted Odds of Age- and Size-Appropriate Child Restraint System Use eTable 10. Guideline Adherence Outcome Including Participants Who Withdrew or Were Lost to Follow-Up Considering All Not Guideline Adherent [file jamanetwopen-e2533912-s002.pdf]

Supplemental Online Content

Macy ML, Pollock B, Kendi S, et al. Multimodal intervention and child passenger safety guideline adherence in young children: a sequential, multiple assignment, randomized trial. *JAMA Netw. Open*. 2025;8(9):e2533912. doi:10.1001/jamanetworkopen.2025.33912

eMethods.

eTable 1. Child Passenger Restraint System Age and Size Criteria for Dichotomous Outcome Determination

eTable 2. Counts of Child Passenger Restraint Systems by Lower Weight Limit

eTable 3. Counts of Child Passenger Restraint Systems by Upper Weight Limit

eTable 4. Counts of Child Passenger Restraint Systems by Lower Height Limit

eTable 5. Counts of Child Passenger Restraint Systems by Upper Height Limit

eTable 6. Phase I: Unadjusted and Adjusted Odds of Child Passenger Safety Guideline Adherence at 6-Month Follow-Up

eTable 7. Phase II: Unadjusted and Adjusted Odds of Child Passenger Safety Guideline Adherence at 12-Month Follow-Up

eTable 8. Maintenance Effects: Unadjusted and Adjusted Odds of Child Passenger Safety Guideline Adherence at 12-Month Follow-Up

eTable 9. Unadjusted and Adjusted Odds of Age- and Size-Appropriate Child Restraint System Use

eTable 10. Guideline Adherence Outcome Including Participants Who Withdrew or Were Lost to Follow-Up Considering All Not Guideline Adherent

This supplemental material has been provided by the authors to give readers additional information about their work.

© 2025 Macy ML et al. *JAMA Network Open*.

eMethods

Categorization of Child Restraint System Age and Size-Appropriateness
The study team utilized a matrix that presented age and weight relative to the recommended child restraint system (CRS) for each age x weight combination to determine targets for recruitment efforts. During recruitment calls the research coordinator confirmed survey responses, including the caregivers plans for CRS transition. We recruited children who were at least 6 months old because most younger infants (>95%) use a rear-facing CRS according to national scale observational studies such as the National Survey of the Use of Booster Seats. We limited the upper age and size of our recruitment to those younger than 11 years and shorter than 55 inches at enrollment. We anticipated this group would benefit from the use of a booster seat throughout the 12-month study, expecting typical growth of 2 inches per year for a 10-year-old.

In our intervention materials, we placed emphasis on the importance of proper seat belt fit over a specific height threshold for transitioning from a booster seat to an adult seat belt. The study website provided families with details of the 5-step test used by child passenger safety technicians and advocates and corrective feedback in text messages addressed lap and shoulder belt position as well as slouching. The dichotomous outcome of guideline adherence was determined using typical CRS on the U.S. market according to the list published by the American Academy of Pediatrics (AAP) in 2024. The age and size criteria used for the outcome assessment are presented in eTable 1.

The Current Context of Size-Limits for Child Restraint Systems on the US Market in 2025
Manufacturers continue to innovate on CRS design and there are now CRS models available to families that have higher weight and height limits than the thresholds used for our outcome assessment. For example, according to the AAP's *Car Seats: Product Listing for 2025* published on their healthychildren.org website, 28 of 97 rear-facing CRS now accommodate children up to 50 pounds, so children over 40 pounds but less than 50 pounds who are rear-facing in such a CRS would not be appropriately restrained and experiencing a delayed transition if we had such detail on their CRS for assessment in the study. Despite these rear-facing CRS with higher weight limits, 40 pounds remains the most common upper weight limit, documented for 63 of 97 rear-facing CRS on the AAP's listing. We did not obtain the specific CRS weight and height limits for each study participant and note this in our limitations. Families were recruited for this study between 2020 and 2022. Because of cost and convenience, many families use CRS for multiple years and younger siblings frequently use "hand me down" CRS. Older CRS models are less likely to have the higher weight and height limits observed for models that have entered the market in 2025. In eTable 2, eTable 3, eTable 4 and eTable 5, we provide readers with context for the number of 2025 CRS by their size parameters, highlighting the minimum and maximum weight and height thresholds used for our study.

Supplemental statistical methods
We limited covariates in our logistic regression models to randomization strata, caregiver race and ethnicity, and caregiver gender as per our original analysis plan. We ran three unadjusted logistic regression models, three adjusted for randomization strata, and three adjusted for randomization strata, caregiver race and ethnicity, and gender.

By testing three outcomes, we assessed each component of our Sequential, Multiple Assignment, Randomized Trial (SMART) design:

Outcome 1: 6-month outcomes. For this outcome, we compared groups from the initial Phase I randomization dichotomy [(basic TCBD/ABCB intervention versus Enhanced Usual Care (EUC)].

Outcome 2: 12-month maintenance effects. For this outcome we compared groups from the initial Phase I randomization dichotomy (basic TCBD/ABCB intervention versus EUC) who were not eligible for rerandomization. This includes basic TCBD/ABCB intervention group participants who were determined to be guideline adherent at 6 months (who continued to receive mHealth during Phase II) and the entire EUC group, regardless of child passenger safety behaviors at 6 months.

Outcome 3: 12-month SMART design outcomes. For the SMART design outcome, we compared the participants rerandomized at 6 months to Phase II high-intensity intervention (second remote motivational interviewing session and extra text messages) versus low-intensity intervention (continued mHealth alone). These participants were eligible for rerandomization because their 6-month child passenger safety behaviors were not guideline adherent, inclusive of continued plans for a premature transition, using the front seat, or traveling unrestrained.

© 2025 Macy ML et al. *JAMA Network Open*.

We included the baseline randomization strata as a covariate in all adjusted models. In doing so, we accounted for the baseline child passenger safety behaviors in the sample, child age groups, and child weight. We accounted for 6-month child passenger safety behaviors in our 12-month outcome models as these behaviors determined eligibility for rerandomization. We tested for interactions between randomization strata and intervention groups in each model and the interaction terms were not significant in any model. Results were not changed when including an adjustment for children who crossed a weight or height threshold for premature transition to appropriate CRS.

There were no missing data for the caregiver gender and caregiver race and ethnicity variables. We discuss our handling of missing weight and height data in the manuscript. We excluded cases lost to follow-up from our main analyses. We present unadjusted odds ratios for the intervention groups and adjusted odds ratios with 95% confidence intervals and p-values for all variables included in these models in eTable 6, eTable 7, and eTable 8. The odds ratios from models adjusted for randomization strata have been reported to Clincialtrials.org.

We present results of analyses using predictor and covariates outlined above where the dichotomous outcome includes only age- and size-appropriate CRS use (for the child's usual CRS, without consideration for seating location or unrestrained behavior) in eTable 9. We performed a post-hoc sensitivity analysis considering all participants who withdrew or were lost to follow-up as not guideline adherent at 6 months. Similarly, we considered participants who withdrew or were lost to follow-up between 6 and 12-months as not guideline adherent in sensitivity analyses of 12-month outcome assessments. These results are presented in eTable 10.

We assessed for differences between the participants within the rerandomization groups and across all four 6-month arms using chi-square tests for categorical variables and t-tests for caregiver age. Caregiver race and ethnicity was the only variable that was significantly different across all four 6-month arms and was an *a priori* variable selected for inclusion in the models. When comparing participants in the rerandomization groups, we found no statistically significant differences. The parent age was significantly different between high-intensity and low-intensity groups, p-value of 0.025. Including parent age as a covariate in the model of the SMART design outcome comparing high- and low-intensity groups did not change the findings.

© 2025 Macy ML et al. *JAMA Network Open*.

eTable 1: Child Passenger Restraint System Age and Size Criteria for Dichotomous Outcome Determination

**Child Passenger Restraint System**	**Classification**	**Age**	**Weight**	**Condition**	**Height**
		Years	Pounds		Inches
**Infant Carrier Rear-Facing Only**	Appropriate	Any	<35	AND	<32
	Delayed Transition	Any	≥35	OR	≥32
**Rear-Facing Convertible/3:1**	Appropriate	Any	<40	AND	<40
	Delayed Transition	Any	≥40	OR	≥40
**Forward-Facing with Harness**	Premature Transition	Any	<22	OR	<28
		<3	<40	AND	<40
	Appropriate	3 +	≥22 & <65	AND	≥28 & <49
	Delayed Transition	Any	≥65	OR	≥49
**High Back Booster**	Premature Transition	Any	<40	OR	<38
		<5	<65	AND	<49
	Appropriate	5 +	≥40 & <100	AND	≥38 &<57
	Delayed Transition	Any	≥100	OR	≥57
**Backless Booster**	Premature Transition	Any	<40	OR	<43
		<5	<65	AND	<49
	Appropriate	5 +	≥40 & <120	AND	≥43 & <57
	Delayed Transition	Any	≥120	OR	≥57
**Seat Belt**	Premature Transition	Any	<100	AND	<57
	Appropriate	Any	≥120	OR	≥57

eTable 2: Counts of Child Passenger Restraint Systems by Lower Weight Limit

**Minimum Weight**	Forward Facing (N=119)	High Back Booster (N=136)	Backless Booster (N=48)
20 pounds	1		1
22 pounds	***83***		
23 pounds	1		
25 pounds	7		
26.5 pounds	8		
30 pounds	19		
40 pounds		***136***	***46***
50 pounds			1

Footnote: Bold indicates threshold used to determine appropriateness. Infant carrier and rear-facing CRS lower limits were not relevant to this study because the youngest eligible children were 6 months old. Total CRS numbers differ from table to table due to variation in the specifications provided on the website.

© 2025 Macy ML et al. *JAMA Network Open*.

eTable 3: Counts of Child Passenger Restraint Systems by Upper Weight Limit

**Maximum Weight**	Infant Carrier (N=81)	Rear Facing (N=97)	Forward Facing (N=108)	High Back Booster (N=114)	Backless Booster (N=49)
20 pounds	1				
22 pounds	3				
30 pounds	38	1			
35 pounds	***39***	3			
40 pounds		***63***	9		
45 pounds		2			
50 pounds		28	2		
65 pounds			***97***		
100 pounds				***71***	13
110 pounds				7	10
120 pounds				36	***26***

Footnote: Bold italics indicates threshold used to determine appropriateness. Total CRS numbers differ from table to table due to variation in the specifications provided on the website.

eTable 4: Counts of Child Passenger Restraint Systems by Lower Height Limit

**Minimum Height**	**Forward Facing (N=186)**	**High Back Booster (N=106)**	**Backless Booster (N=27)**
27 inches	1		
28 inches	***10***		
29 inches	10		
30 inches	2		
32 inches	5		
34 inches	2		
35 inches	4		
38 inches		***17***	
39 inches		4	
40 inches		4	
43 inches		***53***	***23***
44 inches		28	10
45 inches			1
46 inches			2

Footnote: Bold italics indicates threshold used to determine appropriateness. Infant carrier and rear-facing CRS lower limits were not relevant to this study because the youngest eligible children were 6 months old. Total CRS numbers differ from table to table due to variation in the specifications provided on the website.

© 2025 Macy ML et al. *JAMA Network Open*.

eTable 5: Counts of Child Passenger Restraint Systems by Upper Height Limit

**Maximum Height**	Infant Carrier (N=83)	Rear Facing (N=71)	Forward Facing (N=39)	High Back Booster (N=114)	Backless Booster (N=48)
26 inches	1				
29 inches	1				
30 inches	14				
32 inches	***65***				
34 inches	1				
35 inches	1				
37 inches		2			
40 inches		***34***	1		
43 inches		14	5		
44 inches		4			
47 inches		3			
48 inches		6			
49 inches		8	***28***		
50 inches			4		
52 inches				20	
54 inches			1		
57 inches				***71***	***37***
58 inches				1	
60 inches				4	4
62 inches					1
63 inches				17	6
64 inches				1	

Footnote: Bold italics indicates threshold used to determine appropriateness. Total CRS numbers differ from table to table due to variation in the specifications provided on the website. Upper height limits can be provided in terms of the position of the child's head relative to the top of the CRS and therefore do not have specified standing height listed.

© 2025 Macy ML et al. *JAMA Network Open*.

eTable 6: Phase I: Unadjusted and Adjusted Odds of Child Passenger Safety Guideline Adherence at 6-Month Follow-up

Predictor Variables and Covariates	Unadjusted Odds of Guideline Adherence (95% CI)	Adjusted [a] Odds of Guideline Adherence (95% CI)	Adjusted [b] Odds of Guideline Adherence (95% CI)
**Intervention Group**			
Enhanced Usual Care	Referent	Referent	Referent
Basic TCBD/ABCB Intervention, Motivational Interviewing session plus mHealth	1.88 (1.19, 2.95) [c]	1.98 (1.20, 3.28) [d]	2.00 (1.19, 3.35) [e]
**Randomization Strata**			
Rear-Facing Recommended Not Guideline Adherent		Referent	Referent
Rear-Facing Recommended Plans Premature Transition		4.83 (2.21, 10.59) [f]	4.58 (2.05, 10.19) [f]
Forward-Facing Recommended Not Guideline Adherent		8.80 (4.42, 17.53) [f]	8.75 (4.32, 17.73) [f]
Forward-Facing Recommended Plans Premature Transition		22.38 (8.89, 56.32) [f]	20.82 (8.15, 53.14) [f]
Booster Seat Recommended Not Guideline Adherent		2.02 (0.87, 4.71)	2.04 (0.86, 4.84)
Booster Seat Recommended Plans Premature Transition		8.90 (2.61, 30.31) [f]	9.13 (2.63, 31.62) [f]
**Caregiver Race and Ethnicity**			
Black, not Hispanic/Latine			0.689 (0.32, 1.49)
Hispanic/Latine, any race			Referent
White, not Hispanic/Latine			1.17 (1.01, 2.88) [g]
Other/multiple races, not Hispanic/Latine			1.20 (0.52, 2.87)
**Caregiver Gender**			
Male			Referent
Female			0.86 (0.33, 2.23)

[a] Adjusted for randomization strata
[b] Adjusted for randomization strata, caregiver race and ethnicity, caregiver gender
[c] p=0.006, [d] p=0.008, [e] p=0.009, [f] p<0.001, [g] p<0.046

© 2025 Macy ML et al. *JAMA Network Open*.

eTable 7: Phase II: Unadjusted and Adjusted Odds of Child Passenger Safety Guideline Adherence at 12-Month Follow-up

Predictor Variables and Covariates	Unadjusted Odds of Guideline Adherence (95% CI)	Adjusted [a] Odds of Guideline Adherence (95% CI)	Adjusted [b] Odds of Guideline Adherence (95% CI)
**Phase II SMART Arms**			
Low-Intensity Intervention, continued mHealth	Referent	Referent	Referent
High-Intensity Intervention, 2nd Motivational Interviewing session, extra mHealth	0.89 (0.49, 1.63)	0.87 (0.45, 1.66)	0.83 (0.43, 1.62)
**Randomization Strata**			
Rear-Facing Recommended Not Guideline Adherent		Referent	Referent
Rear-Facing Recommended Plans Premature Transition		0.82 (0.27, 2.50)	0.78 (0.25, 2.42)
Forward-Facing Recommended Not Guideline Adherent		3.87 (1.77, 8.47) [c]	3.74 (1.65, 8.51) [d]
Forward-Facing Recommended Plans Premature Transition		4.40 (1.01, 19.13) [e]	4.32 (0.95, 19.72)
Booster Seat Recommended Not Guideline Adherent		0.62 (0.21, 1.84)	0.64 (0.21, 1.99)
Booster Seat Recommended Plans Premature Transition		0.63 (0.06, 6.49)	0.59 (0.05, 6.35)
**Caregiver Race and Ethnicity**			
Black, not Hispanic/Latine			1.52 (0.57, 4.10)
Hispanic/Latine, any race			Referent
White, not Hispanic/Latine			2.11 (0.99, 4.55)
Other/multiple races, not Hispanic/Latine			1.22 (0.33, 4.60
**Caregiver Gender**			
Male			Referent
Female			1.22 (0.31, 4.73

[a] Adjusted for randomization strata
[b] Adjusted for randomization strata, caregiver race and ethnicity, caregiver gender
[c] p=0.001, [d] p=0.002, [e] p=0.048

© 2025 Macy ML et al. *JAMA Network Open*.

eTable 8: Maintenance Effects: Unadjusted and Adjusted Odds of Child Passenger Safety Guideline Adherence at 12-Month Follow-up

Predictor Variables and Covariates	Unadjusted Odds of Guideline Adherence (95% CI)	Adjusted [a] Odds of Guideline Adherence (95% CI)	Adjusted [b] Odds of Guideline Adherence (95% CI)
**Maintenance Effects**			
Enhanced Usual Care	Referent	Referent	Referent
Basic TCBD/ABCB Intervention, Adherent at 6 months, continued mHealth	5.18 (2.70, 9.94) [c]	6.86 (3.17, 14.83) [c]	7.57 (3.35, 17.09) [c]
**Randomization Strata**			
Rear-Facing Recommended Not Guideline Adherent		Referent	Referent
Rear-Facing Recommended Plans Premature Transition		0.36 (0.12, 1.09)	0.39 (0.13, 1.22)
Forward-Facing Recommended Not Guideline Adherent		1.28 (0.51, 3.19)	1.11 (0.44, 2.82)
Forward-Facing Recommended Plans Premature Transition		2.82 (0.82, 9.68)	2.81 (0.78, 10.09)
Booster Seat Recommended Not Guideline Adherent		0.27 (0.86, 0.83) [d]	0.25 (0.08, 0.80) [e]
Booster Seat Recommended Plans Premature Transition		0.72 (0.13, 3.85)	0.59 (0.11, 3.26)
**Caregiver Race and Ethnicity**			
Black, not Hispanic/Latine			0.25 (0.71, 0.90) [f]
Hispanic/Latine, any race			Referent
White, not Hispanic/Latine			0.82 (0.36, 1.84)
Other/multiple races, not Hispanic/Latine			0.68 (0.22, 2.16)
**Caregiver Gender**			
Male			Referent
Female			0.87 (0.21, 3.61)

[a] Adjusted for randomization strata
[b] Adjusted for randomization strata, caregiver race and ethnicity, caregiver gender
[c] p<0.001, [d] p=0.022, [e] p=0.019, [f] p=0.033

© 2025 Macy ML et al. *JAMA Network Open*.

eTable 9: Unadjusted and Adjusted Odds of Age- and Size-Appropriate Child Restraint System Use

Follow-up Timeframe Intervention Group	Unadjusted Odds of Guideline Adherence (95% CI)	Adjusted [a] Odds of Guideline Adherence (95% CI)
**6-Month Outcome**		
Enhanced Usual Care	Referent	Referent
Basic TCBD/ABCB Intervention, Motivational Interviewing session plus mHealth	1.62 (1.05, 2.50) [b]	1.88 (1.11, 3.18) [c]
**12-Month SMART Outcomes Rerandomized in Phase II**		
Low-Intensity Intervention, continued mHealth	Referent	Referent
High-Intensity Intervention, 2nd Motivational Interviewing session, extra mHealth	1.07 (0.58, 1.95)	1.03 (0.52, 2.04)
**12-Month Maintenance Effects Groups Not Rerandomized**		
Enhanced Usual Care	Referent	Referent
Basic TCBD/ABCB Intervention, Adherent at 6 months, continued mHealth	4.77 (2.32, 9.81) [d]	9.29 (3.70, 23.32) [d]

[a] Adjusted for randomization strata, caregiver race and ethnicity, caregiver gender
[b] $p=0.029$, [c] $p=0.018$, [d] $p<0.001$

eTable 10: Guideline Adherence Outcome Including Participants Who Withdrew or Were Lost to Follow-up Considering All Not Guideline Adherent

Follow-up Timeframe Intervention Group	Unadjusted Odds of Guideline Adherence (95% CI)	Adjusted [a] Odds of Guideline Adherence (95% CI)
**6-Month Outcome**		
Enhanced Usual Care	Referent	Referent
Basic TCBD/ABCB Intervention, Motivational Interviewing session plus mHealth	1.50 (0.97, 2.32)	1.53 (0.94, 2.47)
**12-Month SMART Outcomes Rerandomized in Phase II**		
Low-Intensity Intervention, continued mHealth	Referent	Referent
High-Intensity Intervention, 2nd Motivational Interviewing session, extra mHealth	0.94 (0.52, 1.68)	0.88 (0.47, 1.64)
**12-Month Maintenance Effects Groups Not Rerandomized**		
Enhanced Usual Care	Referent	Referent
Basic TCBD/ABCB Intervention, Adherent at 6 months, continued mHealth	3.50 (1.96, 6.27) [b]	4.18 (2.11, 8.29) [b]

[a] Adjusted for randomization strata, caregiver race and ethnicity, caregiver gender
[b] $p<0.001$

© 2025 Macy ML et al. *JAMA Network Open*.
