# Supplementary material for: Multimodal Intervention and Child Passenger Safety Guideline Adherence in Young Children: A Sequential, Multiple-Assignment, Randomized Clinical Trial
Source: JAMA Netw Open. 2025 Sep 29;8(9):e2533912. doi: 10.1001/jamanetworkopen.2025.33912 (PMC12481228; doi:10.1001/jamanetworkopen.2025.33912)
Supplement: Supplement 3. — Data Sharing Statement [file jamanetwopen-e2533912-s003.pdf]

Data Sharing Statement

Macy. Multimodal Intervention and Child Passenger Safety Guideline Adherence in Young Children. *JAMA Netw Open*. Published September 29, 2025.
doi:10.1001/jamanetworkopen.2025.33912

Data
Additional Information: Clinicaltrials.gov Identifier: NCT04238247
Data available: Yes
Data types: Deidentified participant data, Data dictionary
How to access data: mmacy@luriechildrens.org
When available: With publication

Supporting Documents
Document types: Statistical/analytic code, Informed consent form
How to access documents: mmacy@luriechildrens.org
When available: With publication

Additional Information
Who can access the data: anyone requesting the data
Types of analyses: for any purpose
Mechanisms of data availability: with a signed data access agreement
